# Supplementary material for: Human cytomegalovirus antagonizes SMC5/6 driven genome silencing via UL35 instituted proteasomal degradation of SLF2
Source: PLoS Pathog. 2026 Oct 12;22(10):e1014709. doi: 10.1371/journal.ppat.1014709 (PMC13690197; doi:10.1371/journal.ppat.1014709)
Supplement: Supplementary file 1 [file ppat.1014709.s007.pdf]

Figure 1B was generated with these images
Detected with: FUSION FX7 imaging system (Vilber Lourmat, Eberhardzell, German)

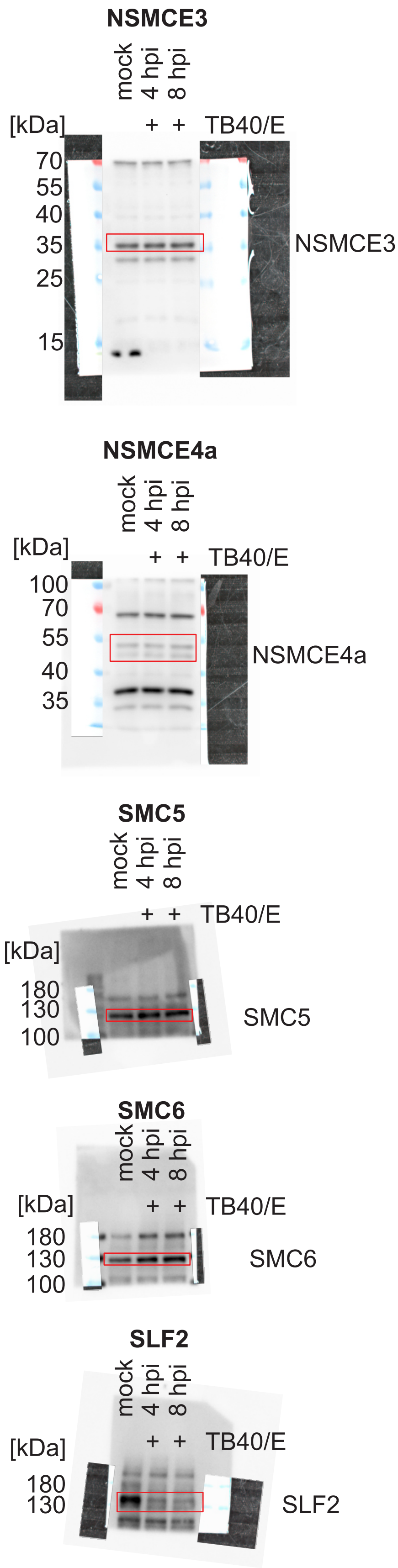

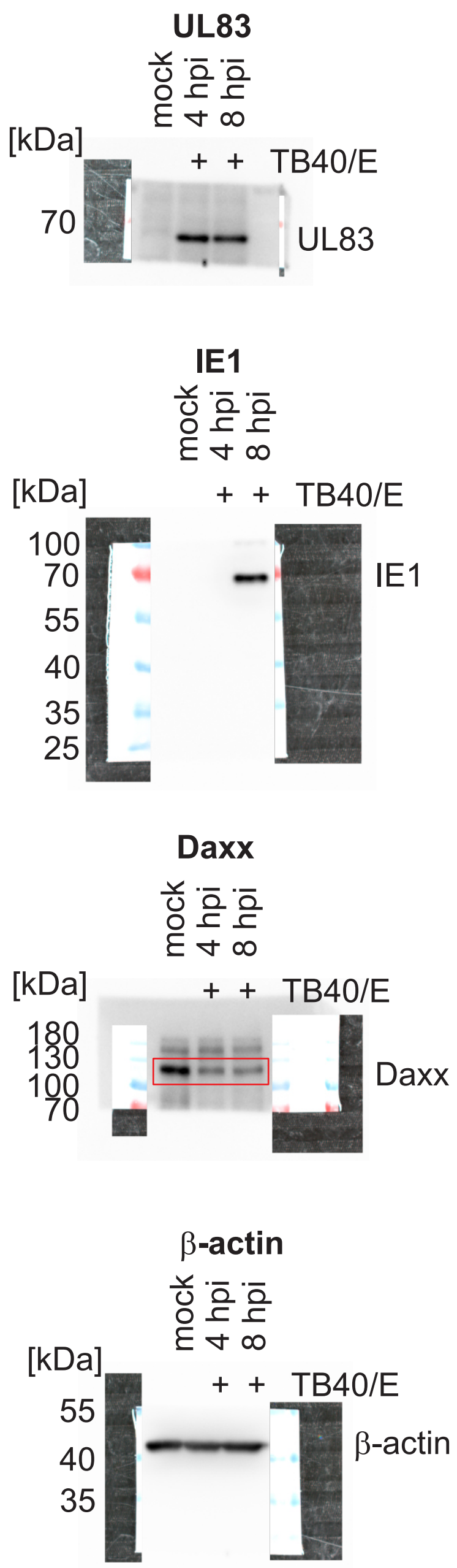

Figure 1D was generated with these images
Detected with: FUSION FX7 imaging system (Vilber Lourmat, Eberhardzell, German)

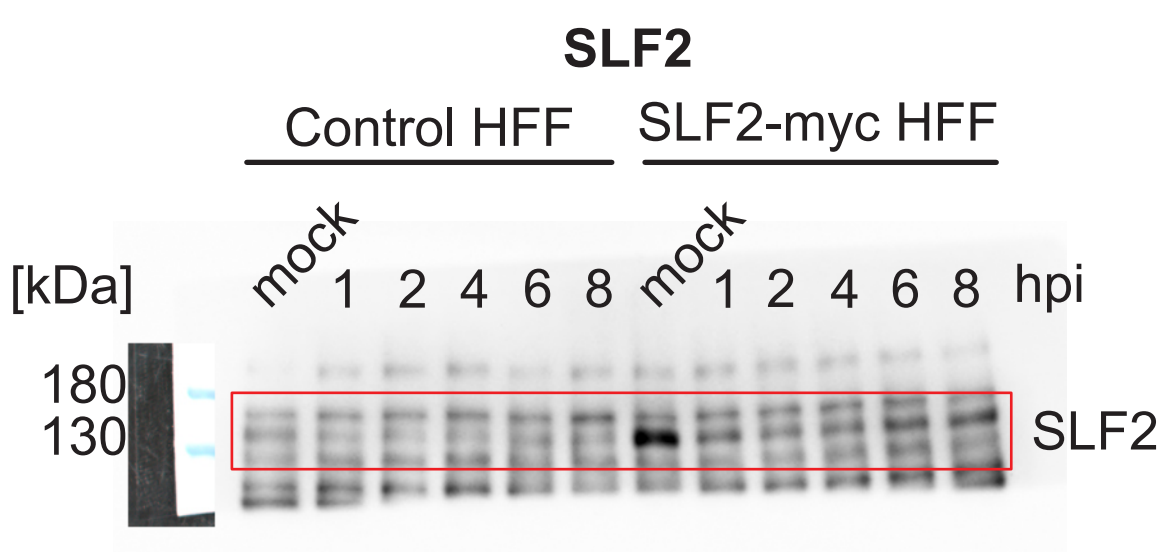

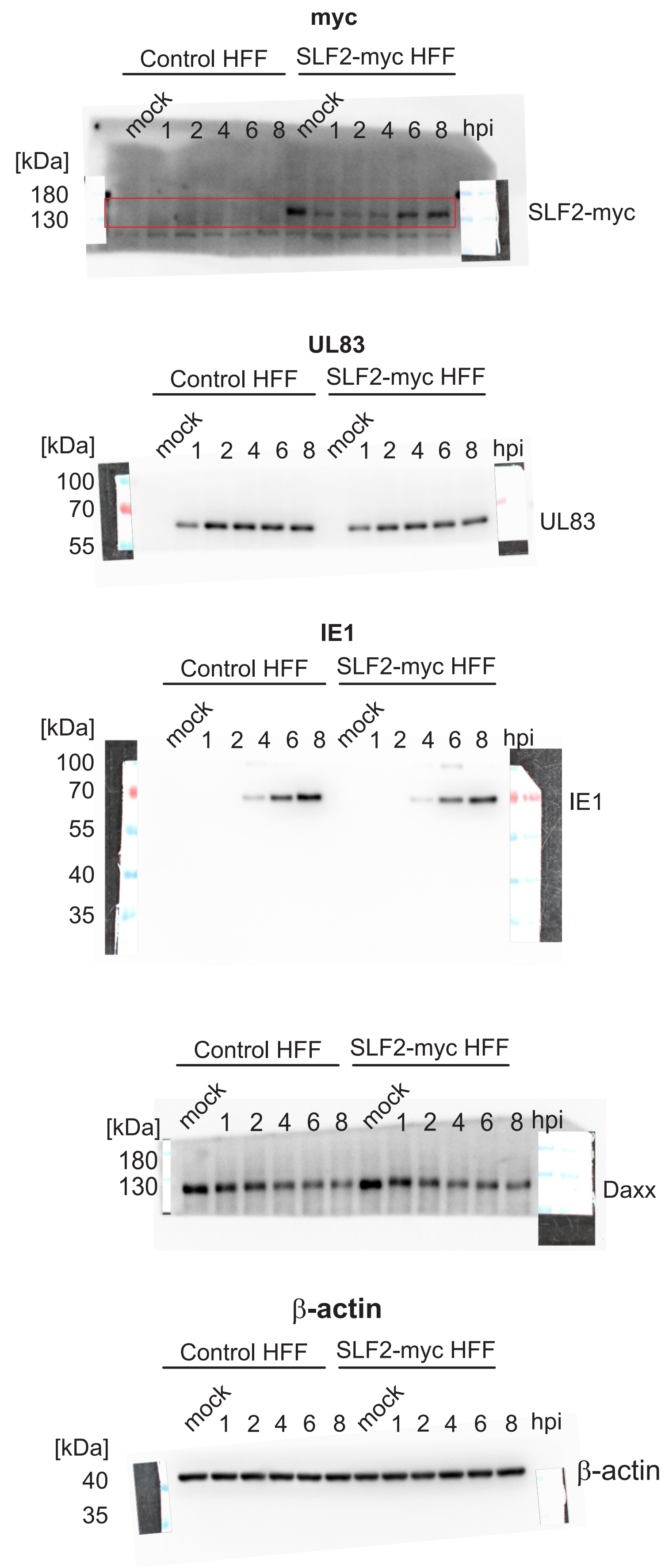

Figure 1E was generated with these images
Detected with: FUSION FX7 imaging system (Vilber Lourmat, Eberhardzell, German)

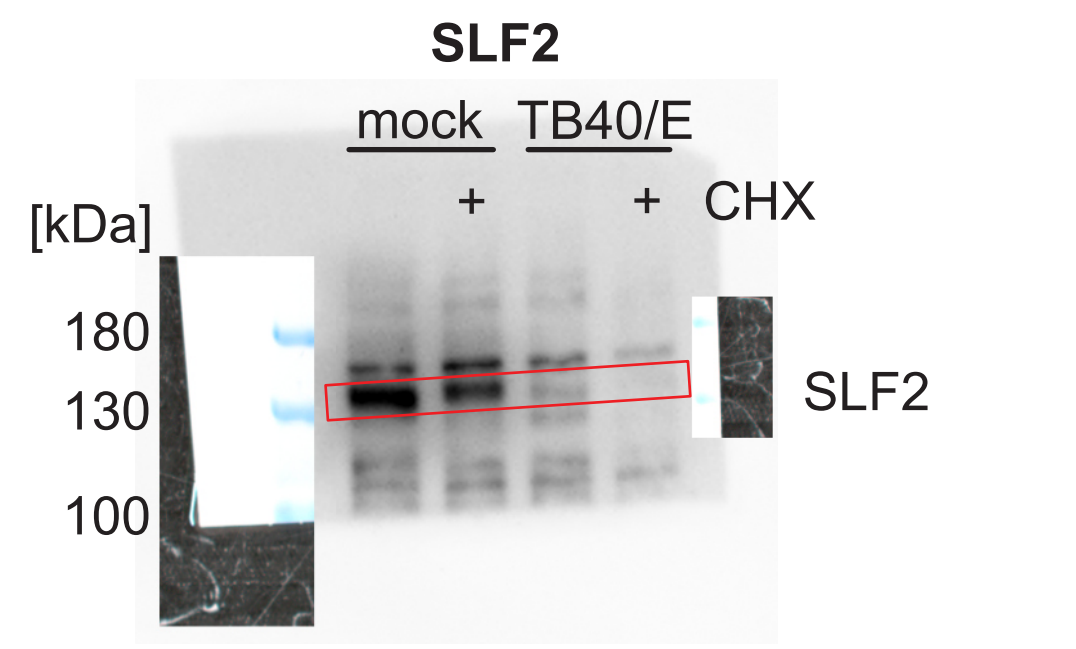

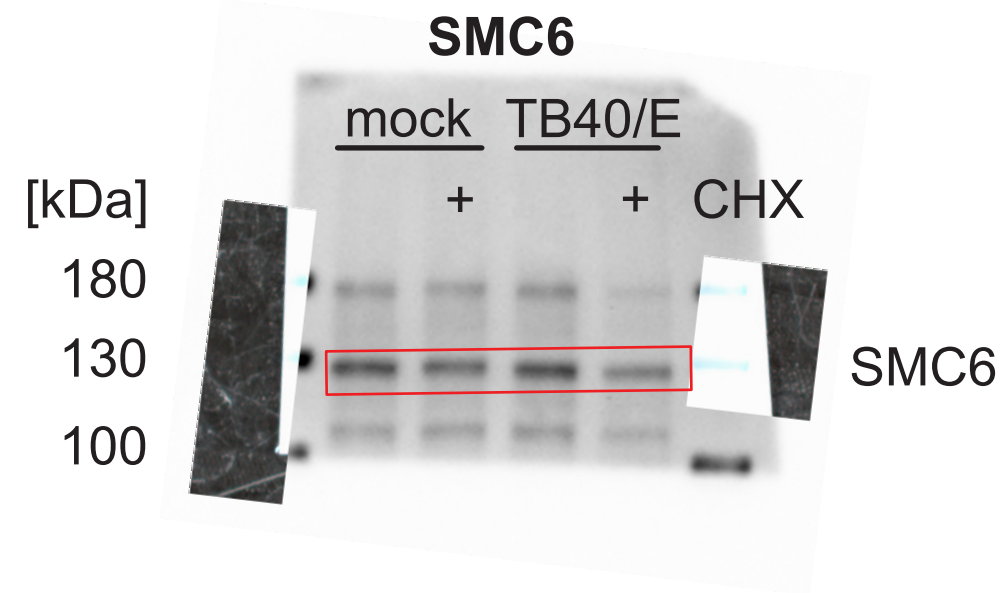

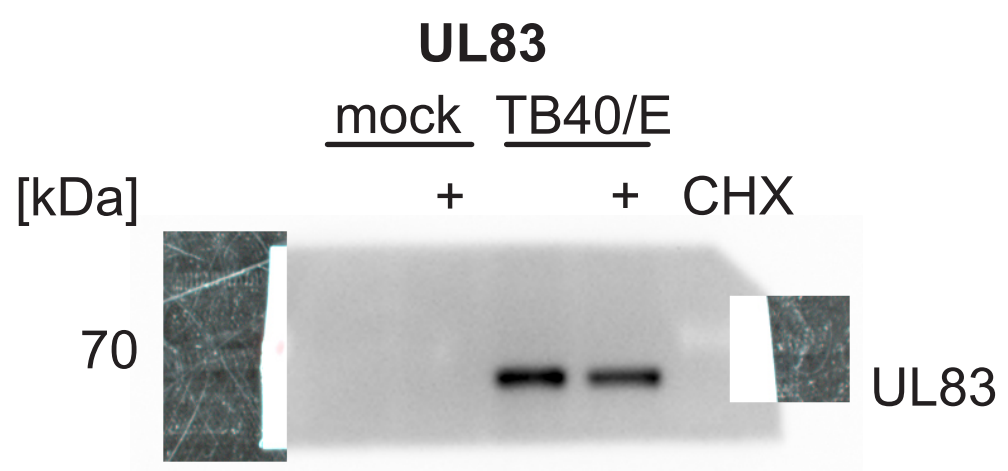

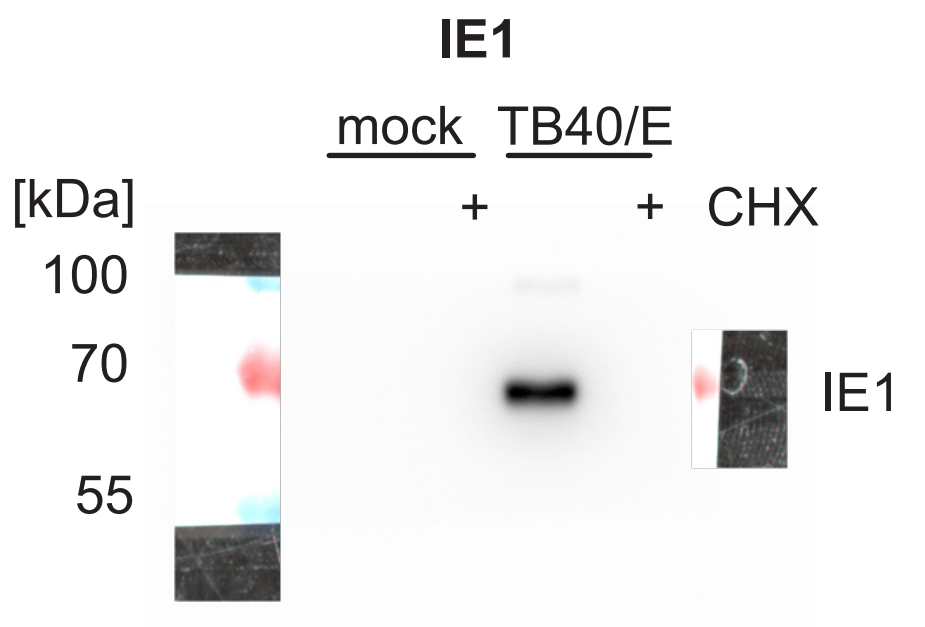

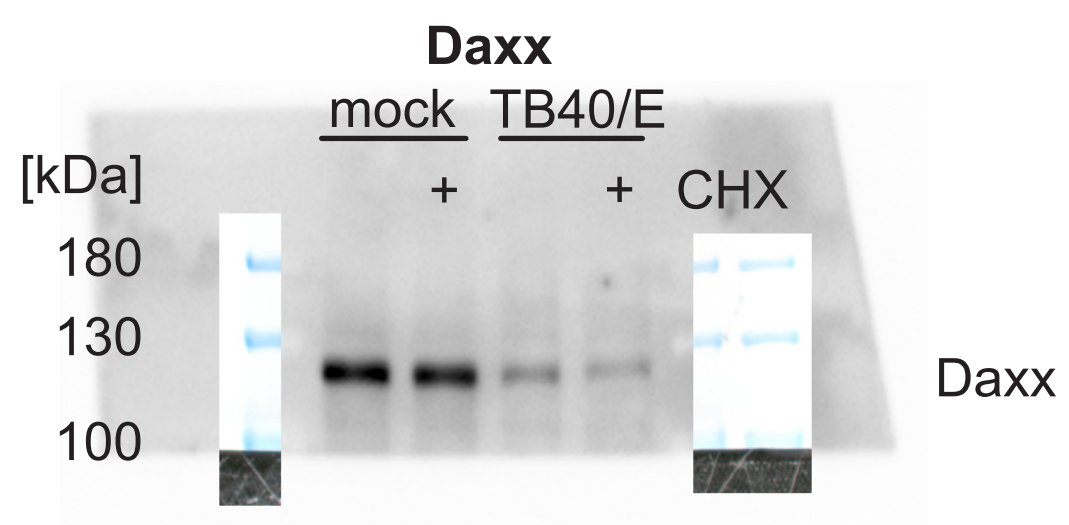

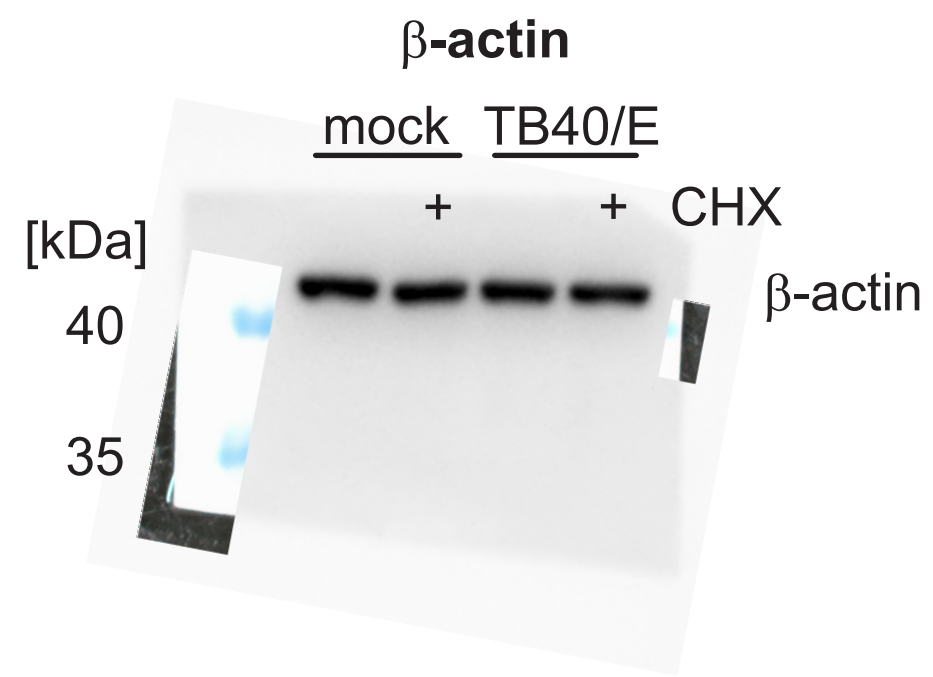

Figure 2C was generated with these images

Detected with: FUSION FX7 imaging system (Vilber Lourmat, Eberhardzell, German)

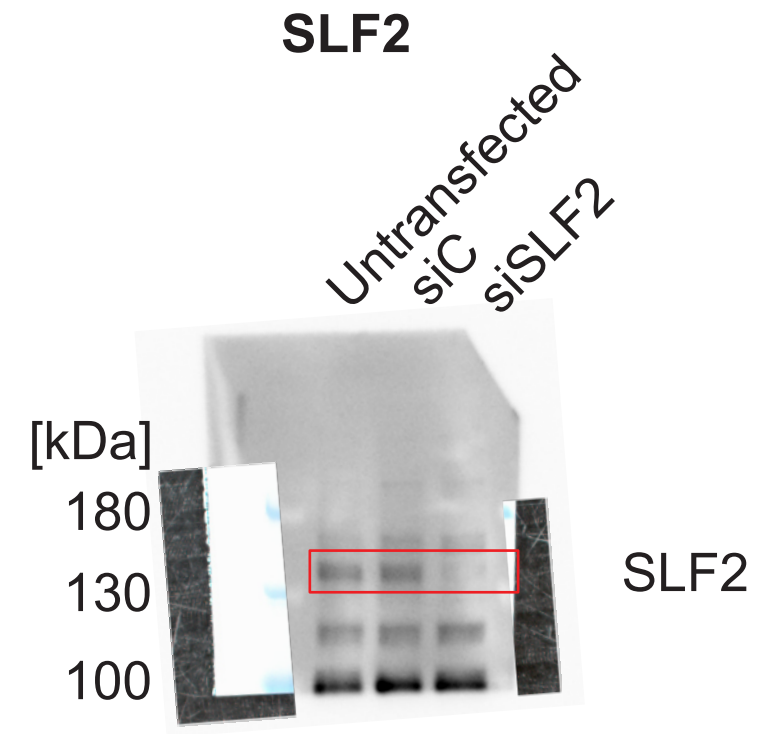

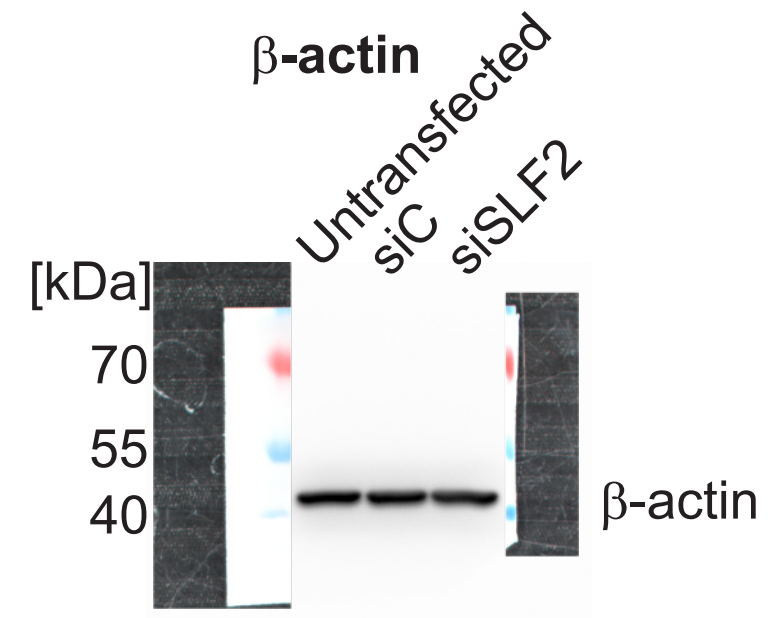

Figure 3A was generated with these images

Detected with: FUSION FX7 imaging system (Vilber Lourmat, Eberhardzell, German)

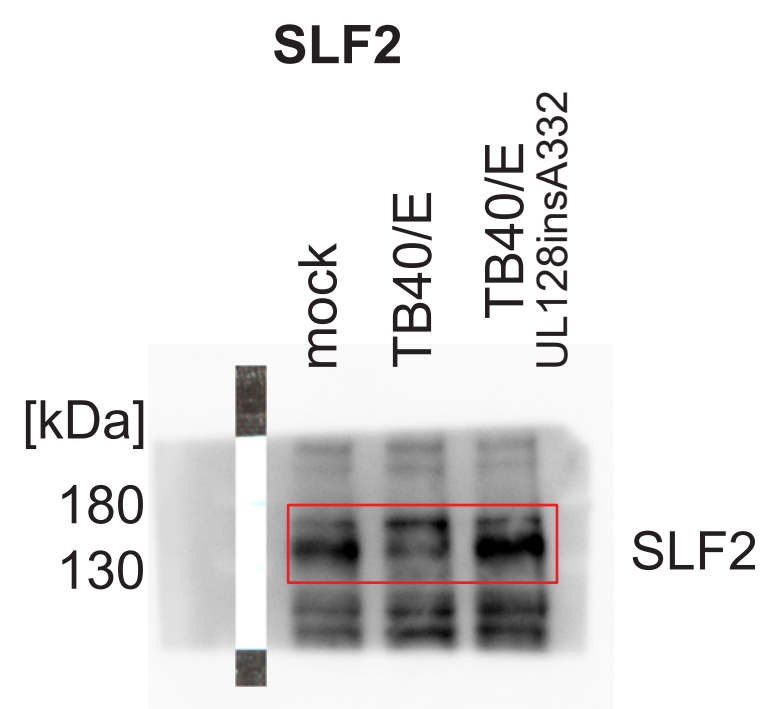

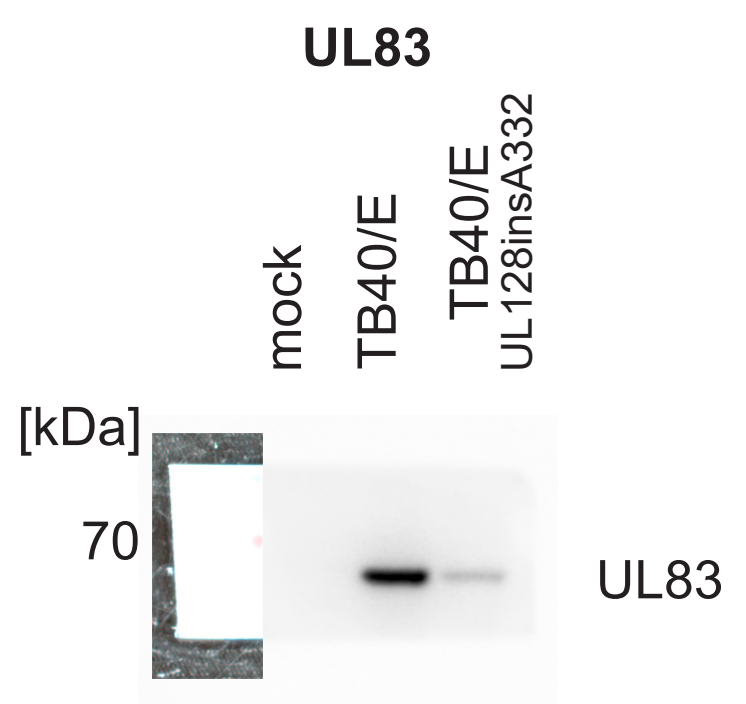

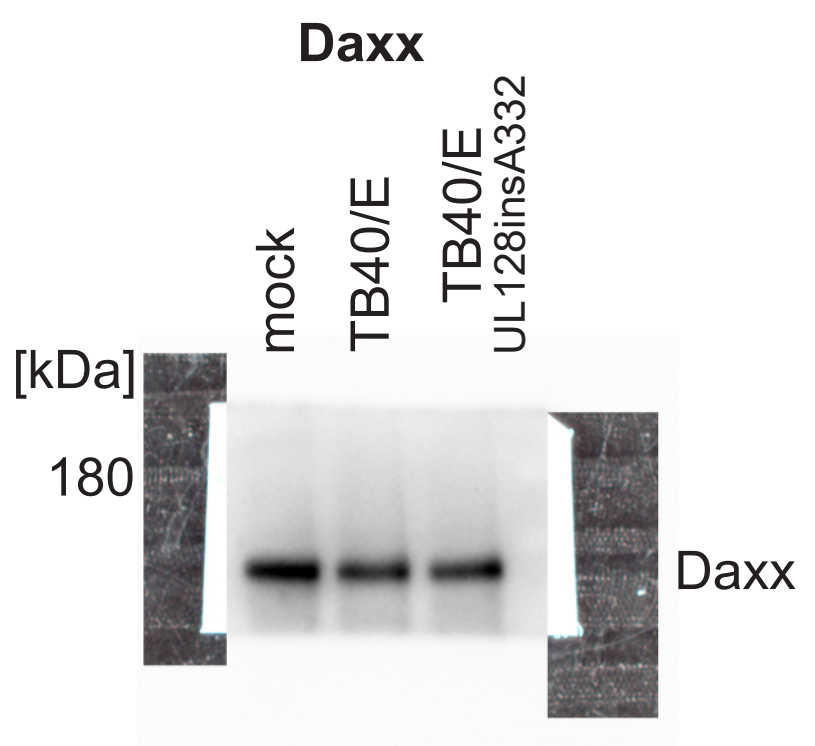

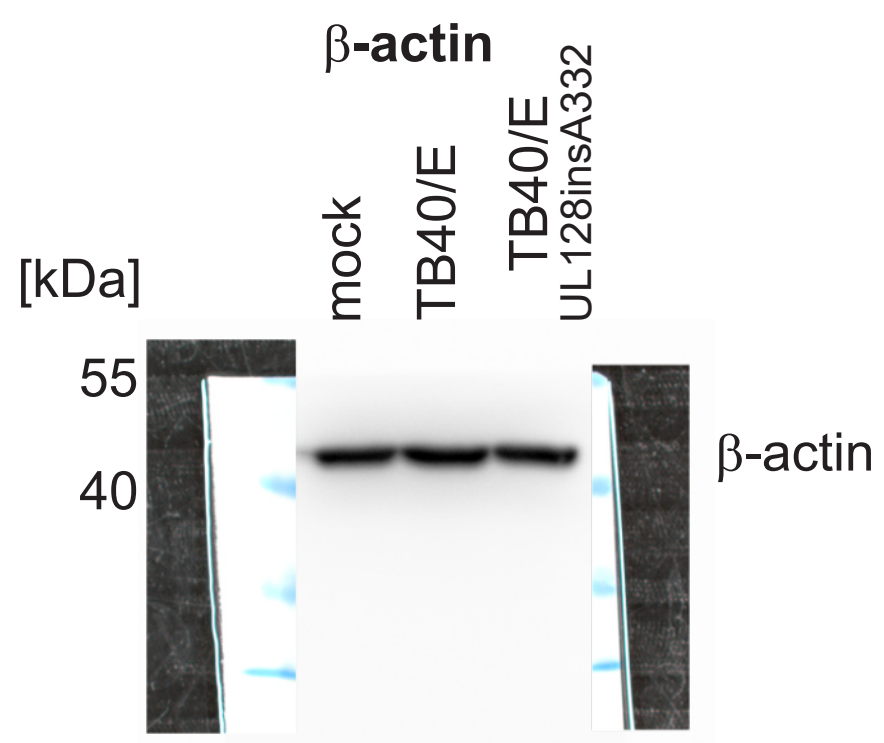

Figure 3B was generated with these images

Detected with: FUSION FX7 imaging system (Vilber Lourmat, Eberhardzell, German)

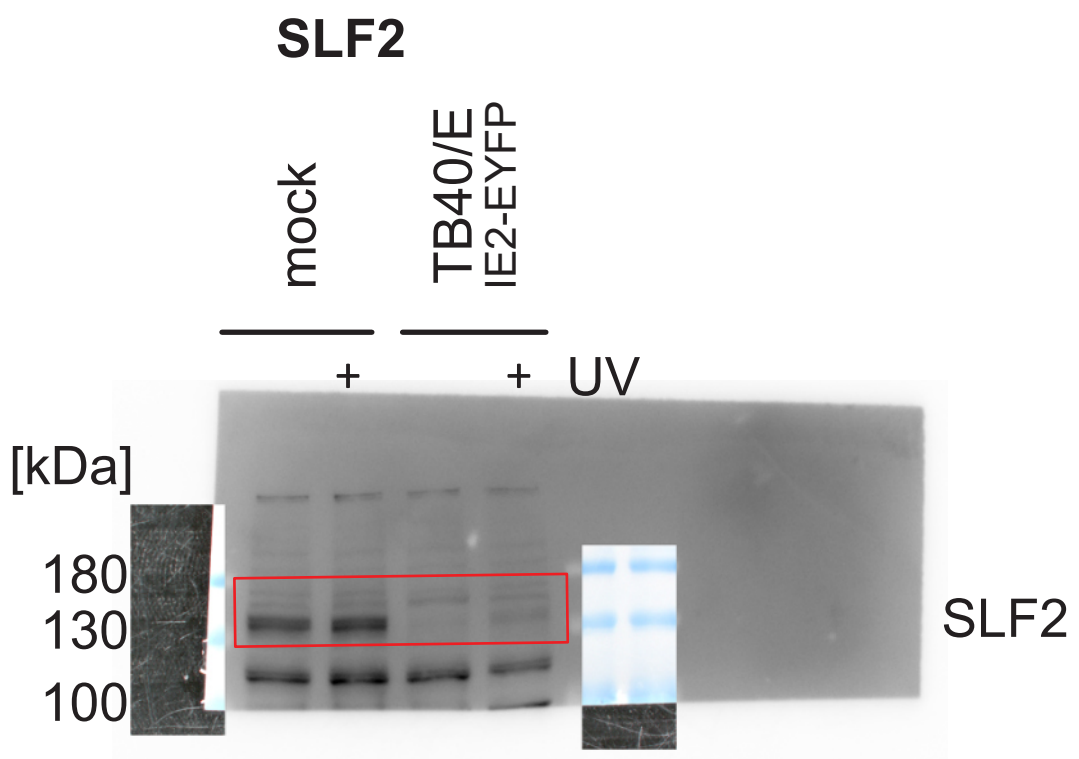

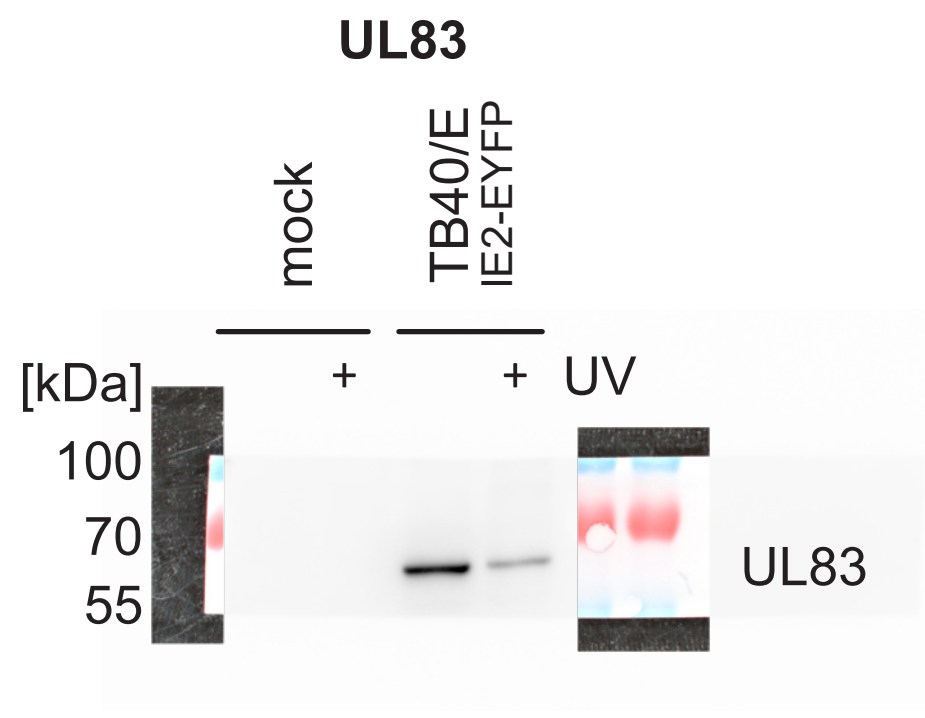

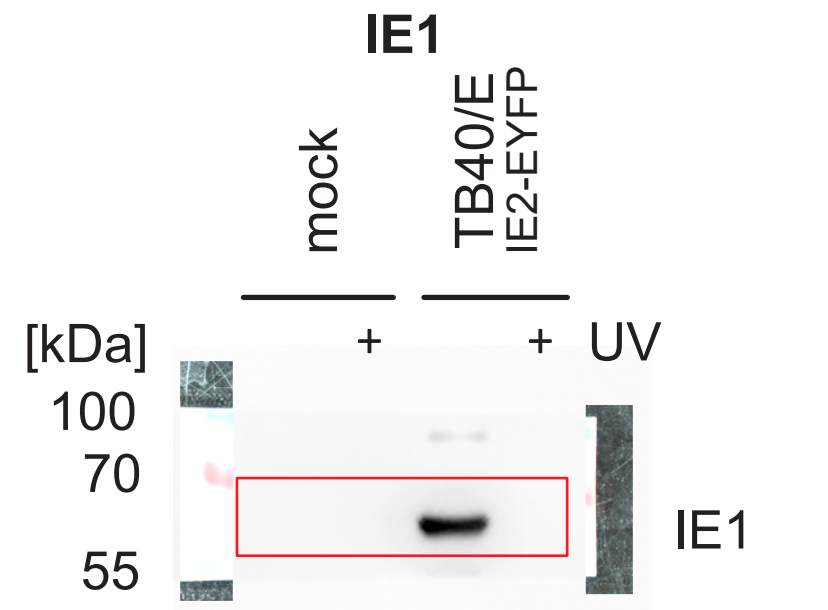

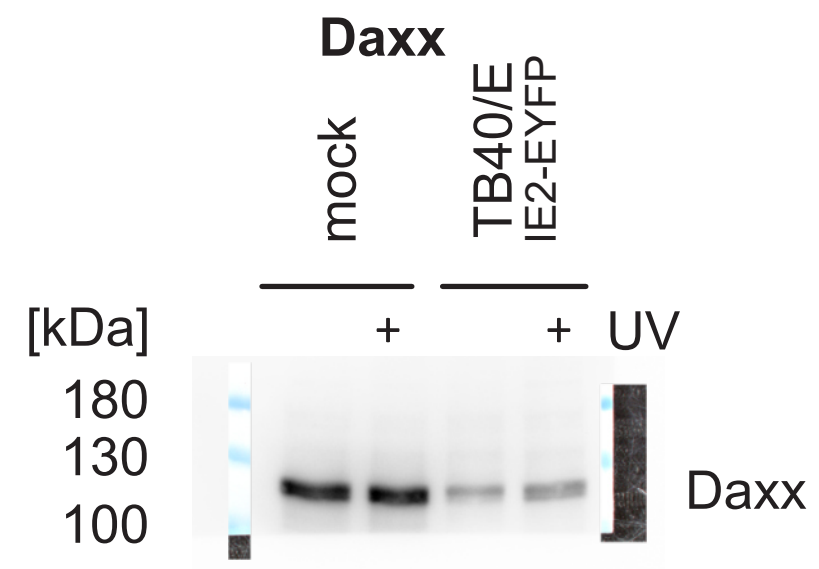

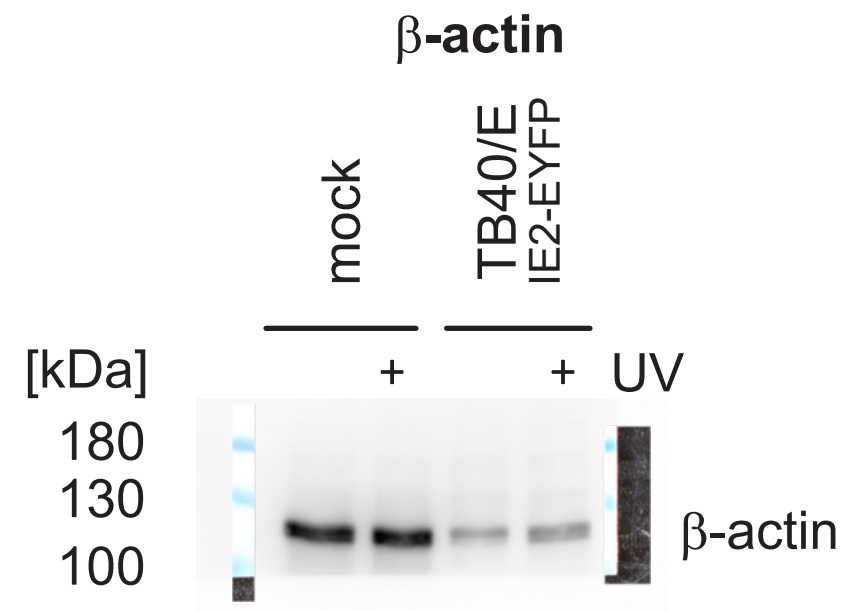

Figure 3E was generated with these images

Detected with: FUSION FX7 imaging system (Vilber Lourmat, Eberhardzell, German)

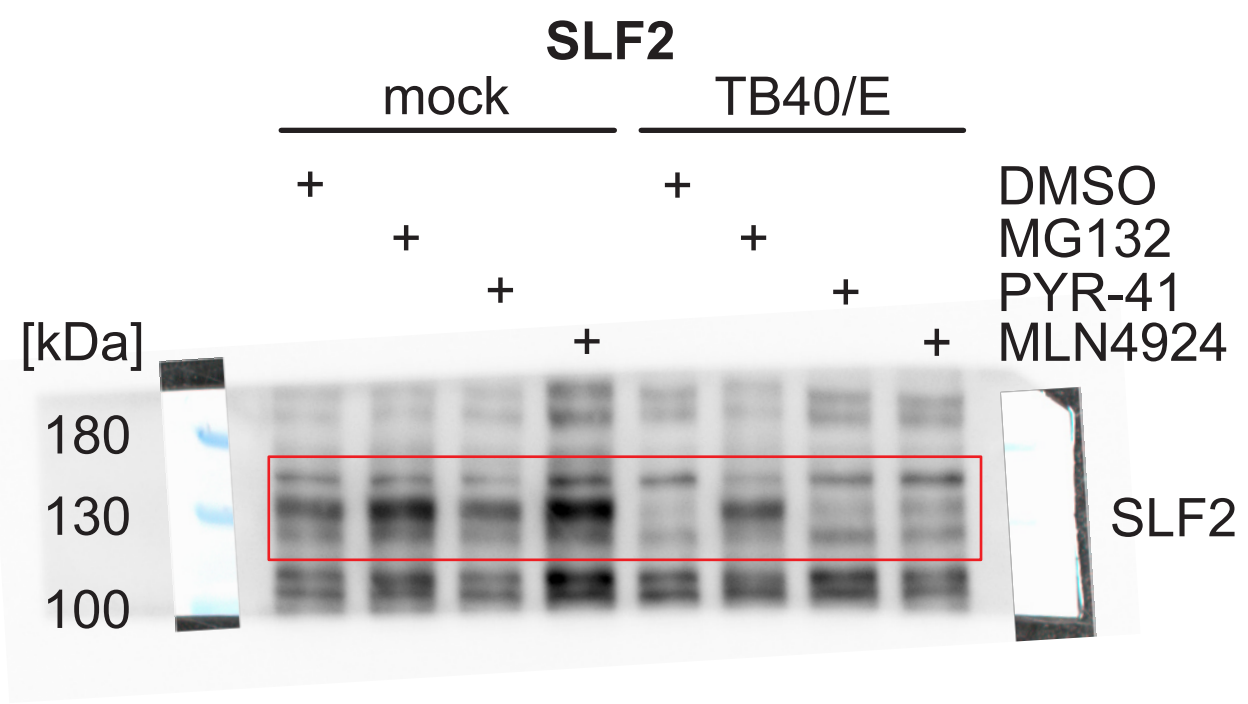

Figure 3H was generated with these images
Detected with: FUSION FX7 imaging system (Vilber Lourmat, Eberhardzell, German)

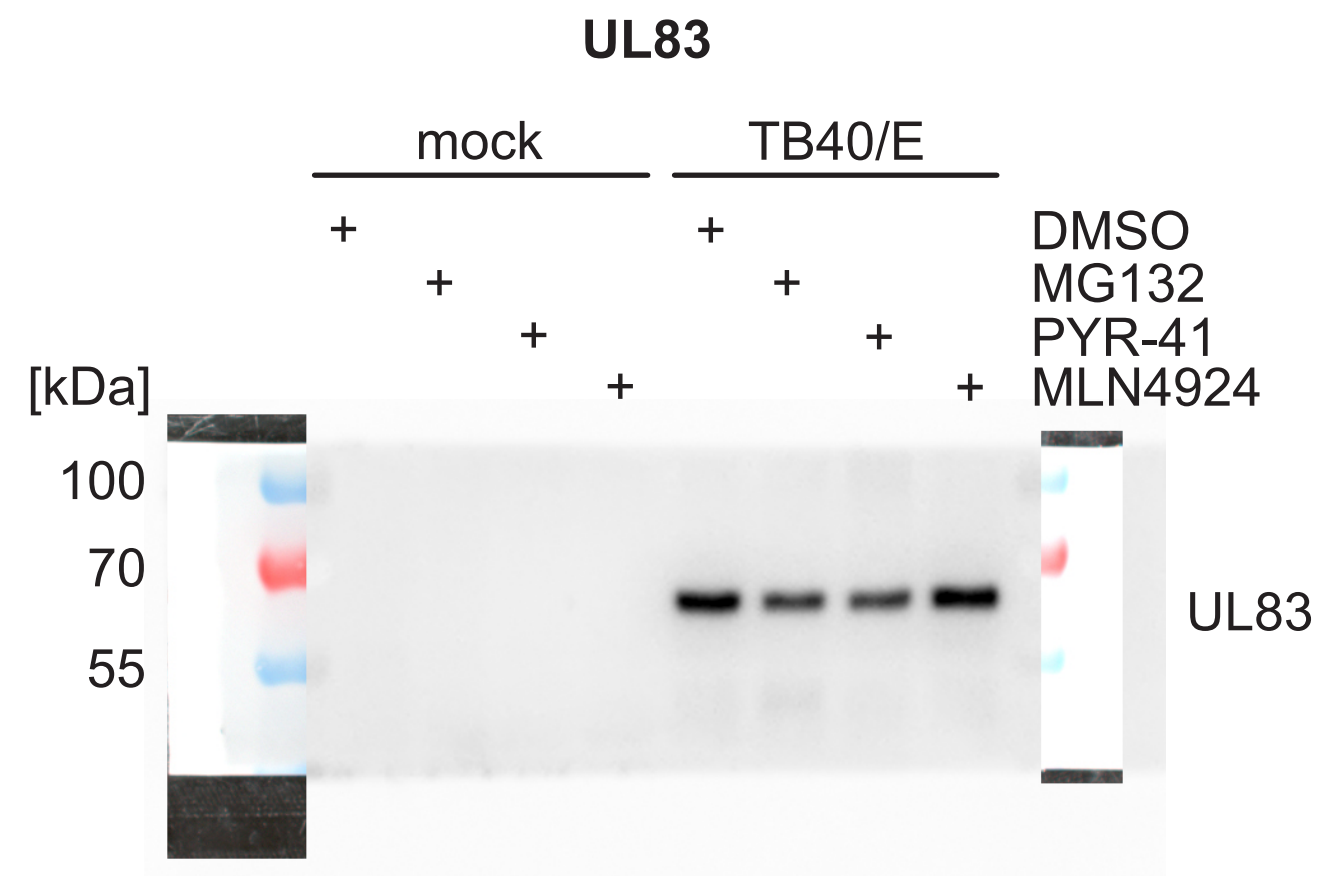

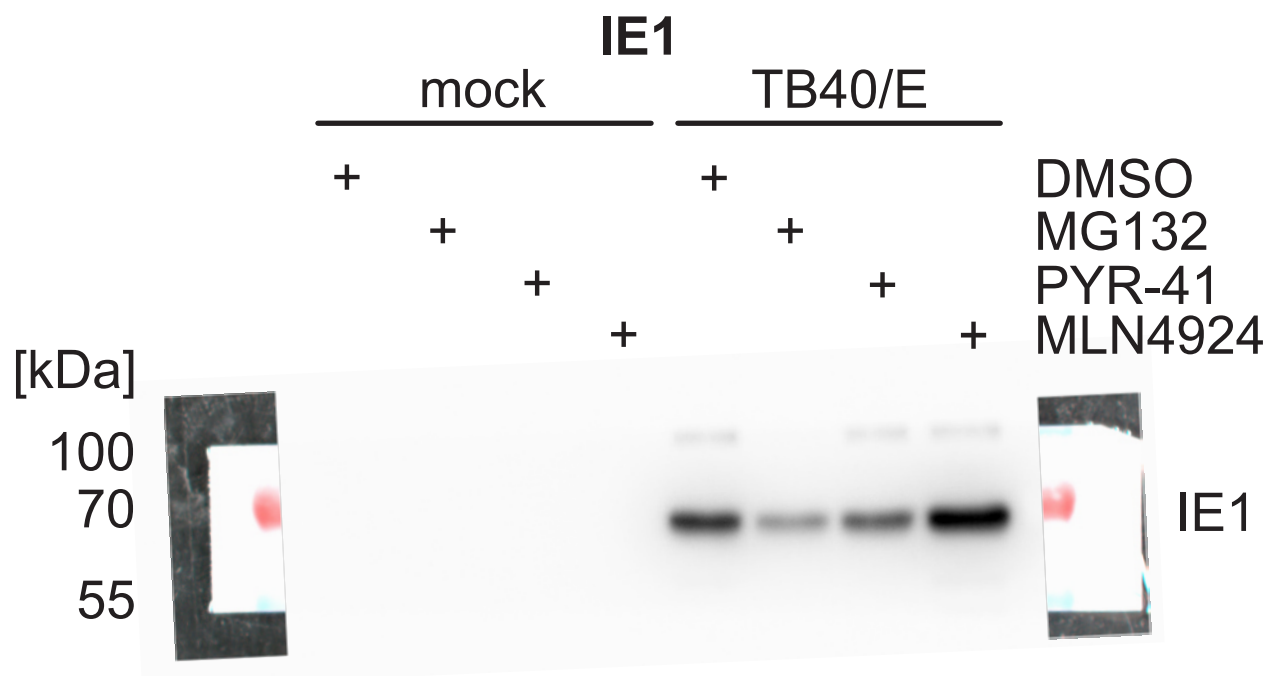

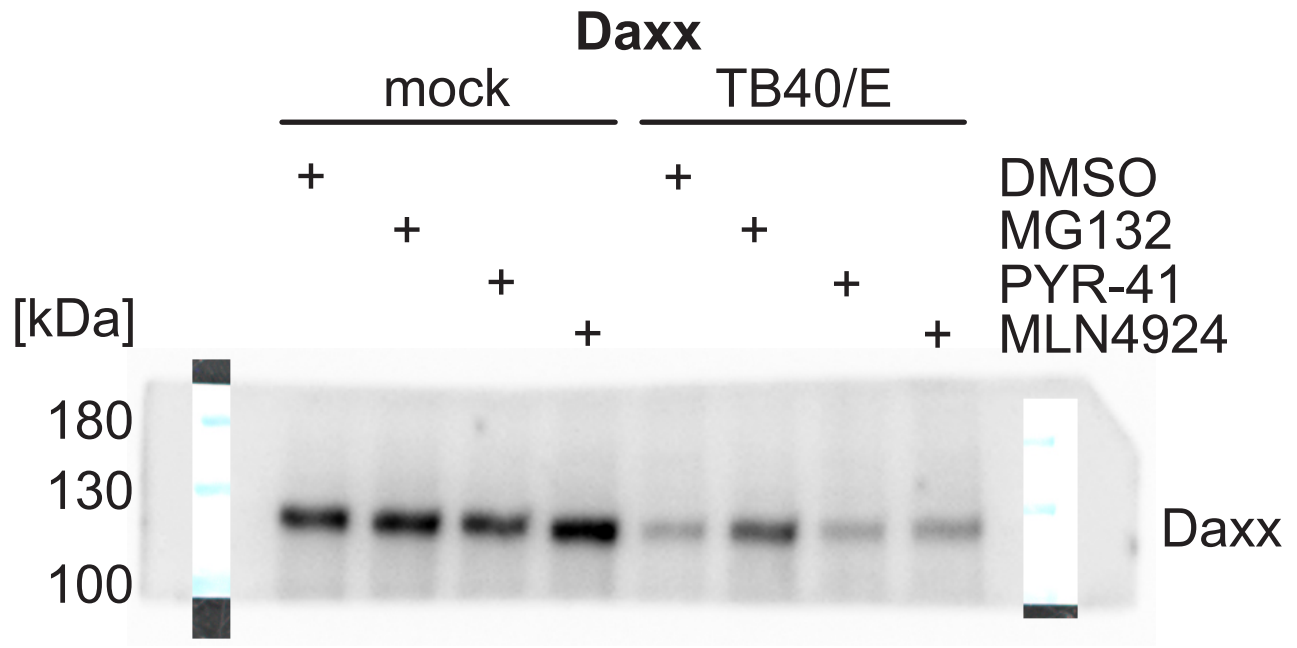

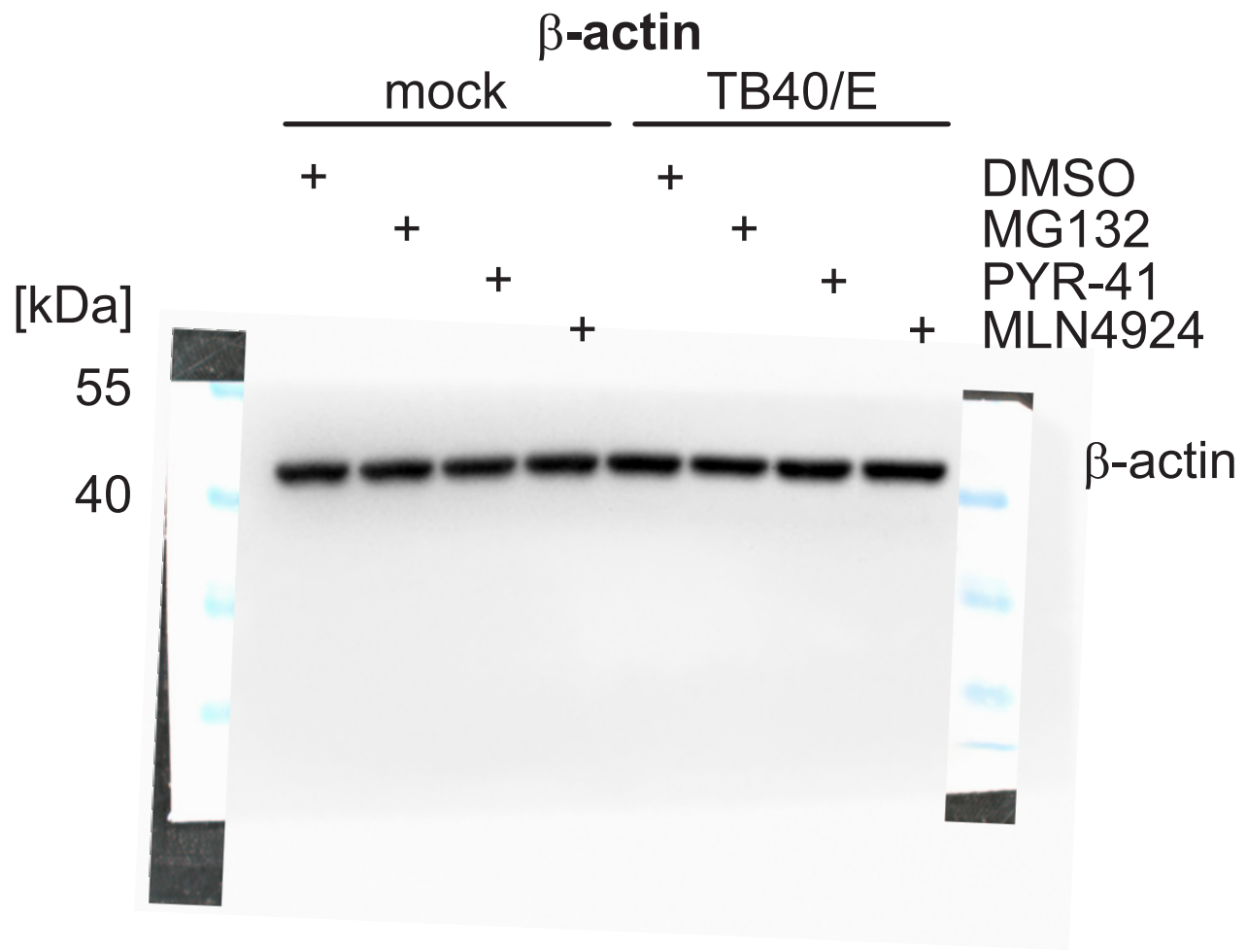

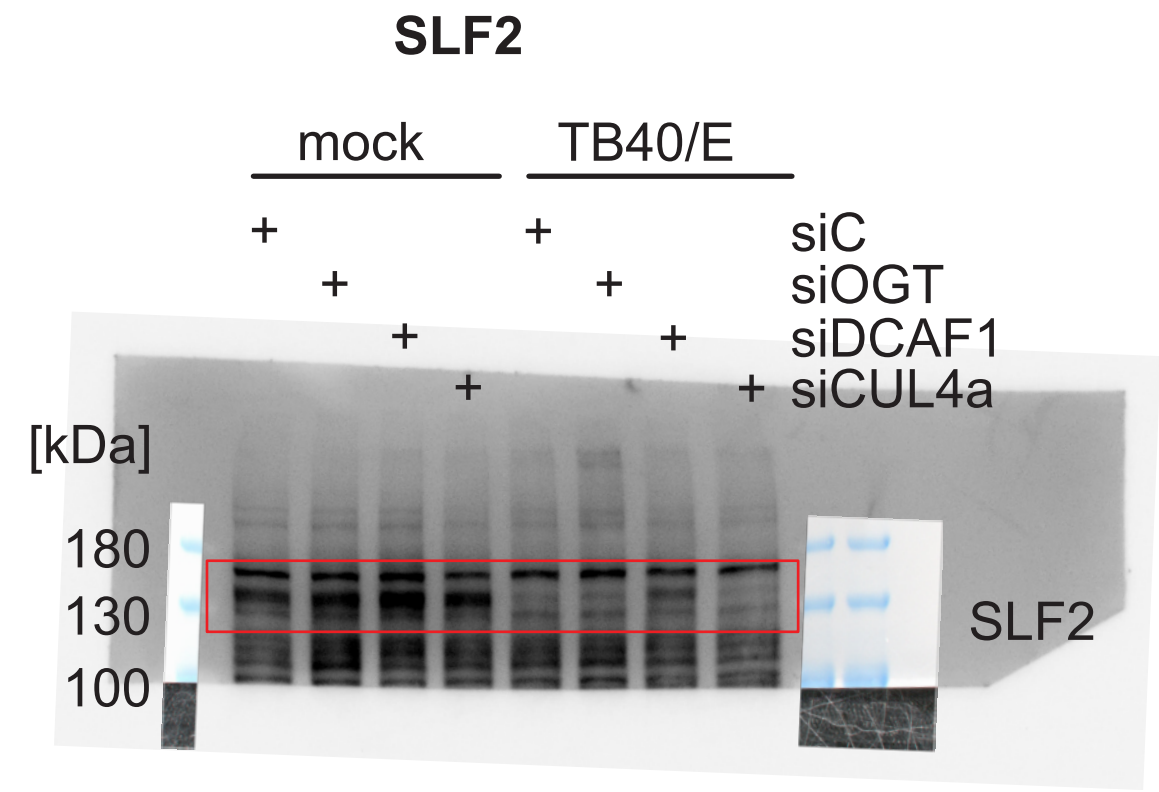

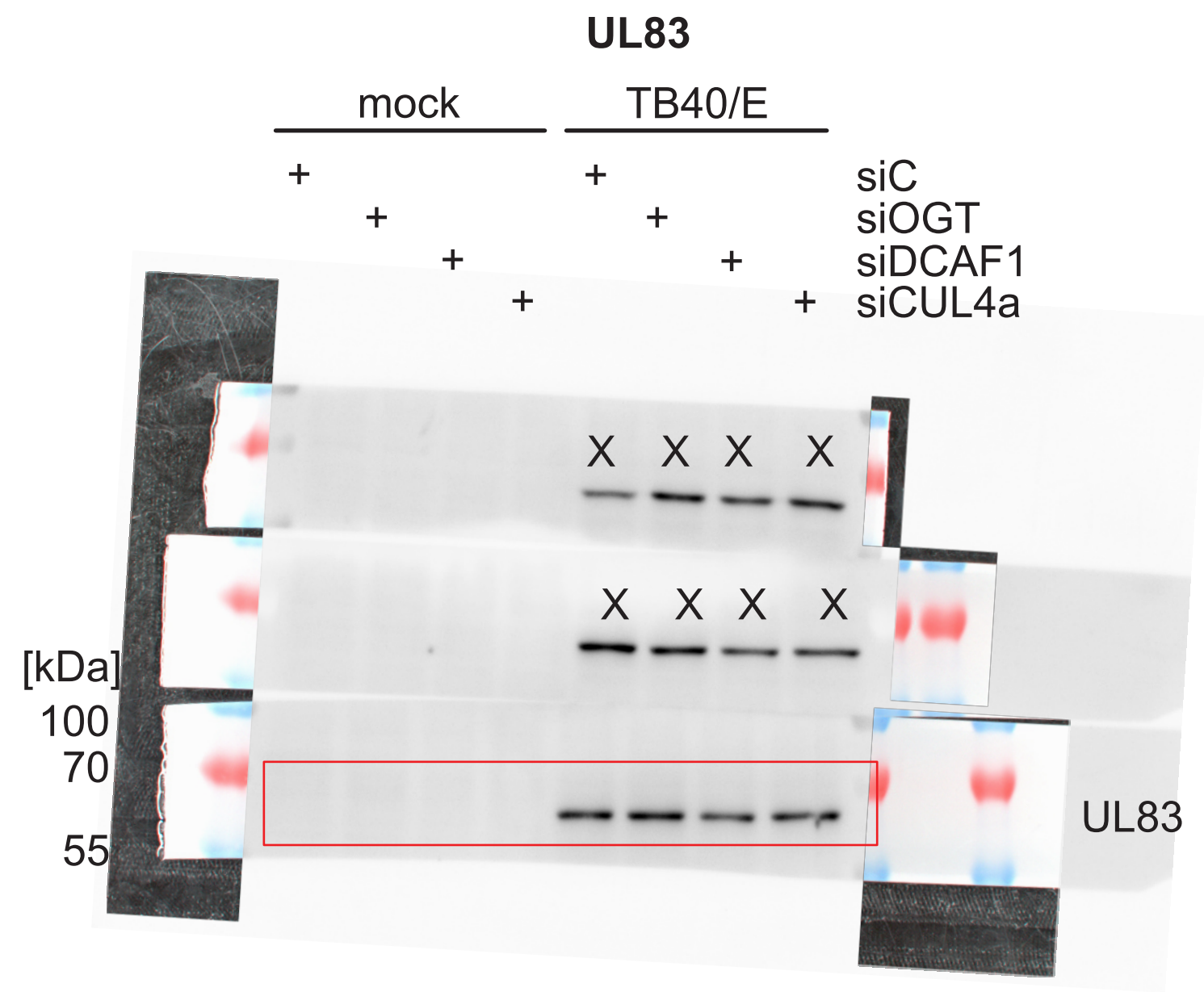

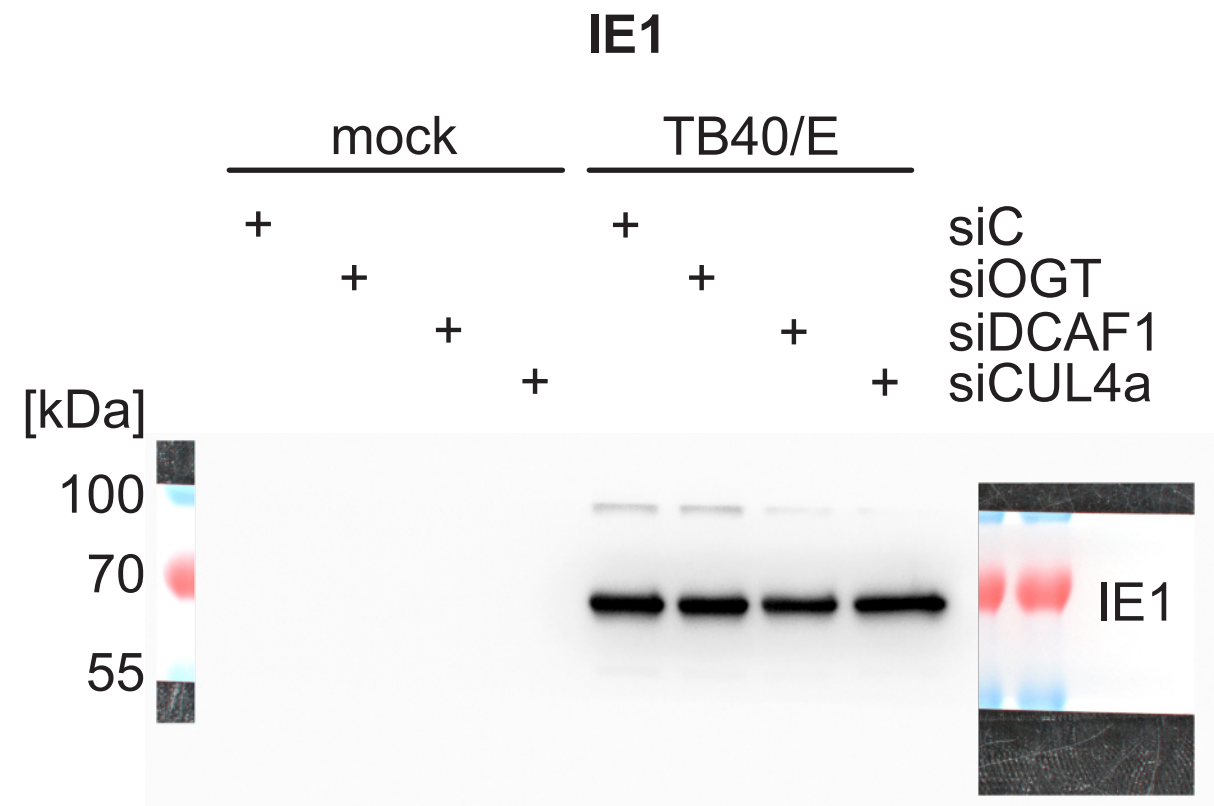

Figure 4A was generated with these images
Detected with: FUSION FX7 imaging system (Vilber Lourmat, Eberhardzell, German)

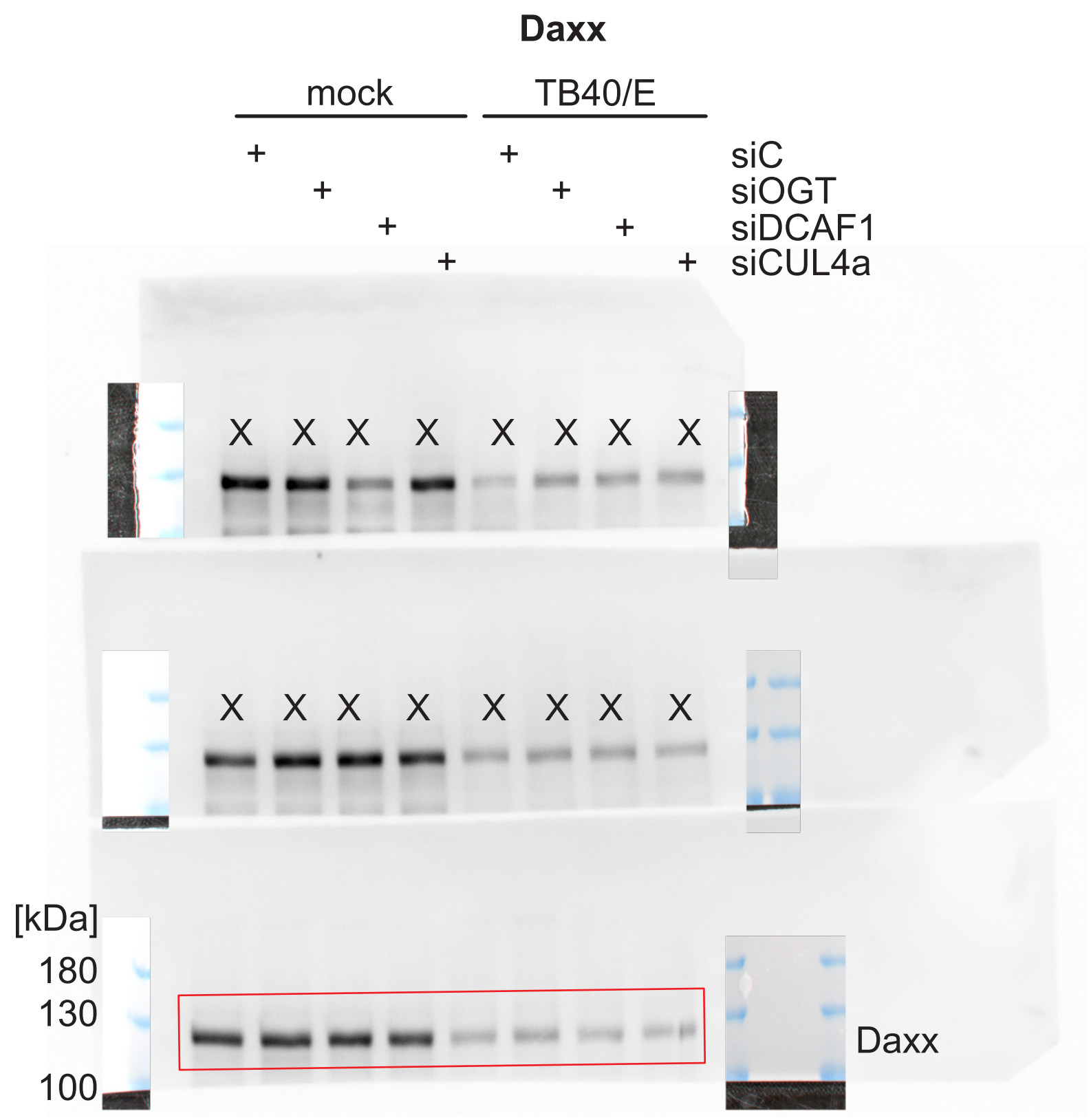

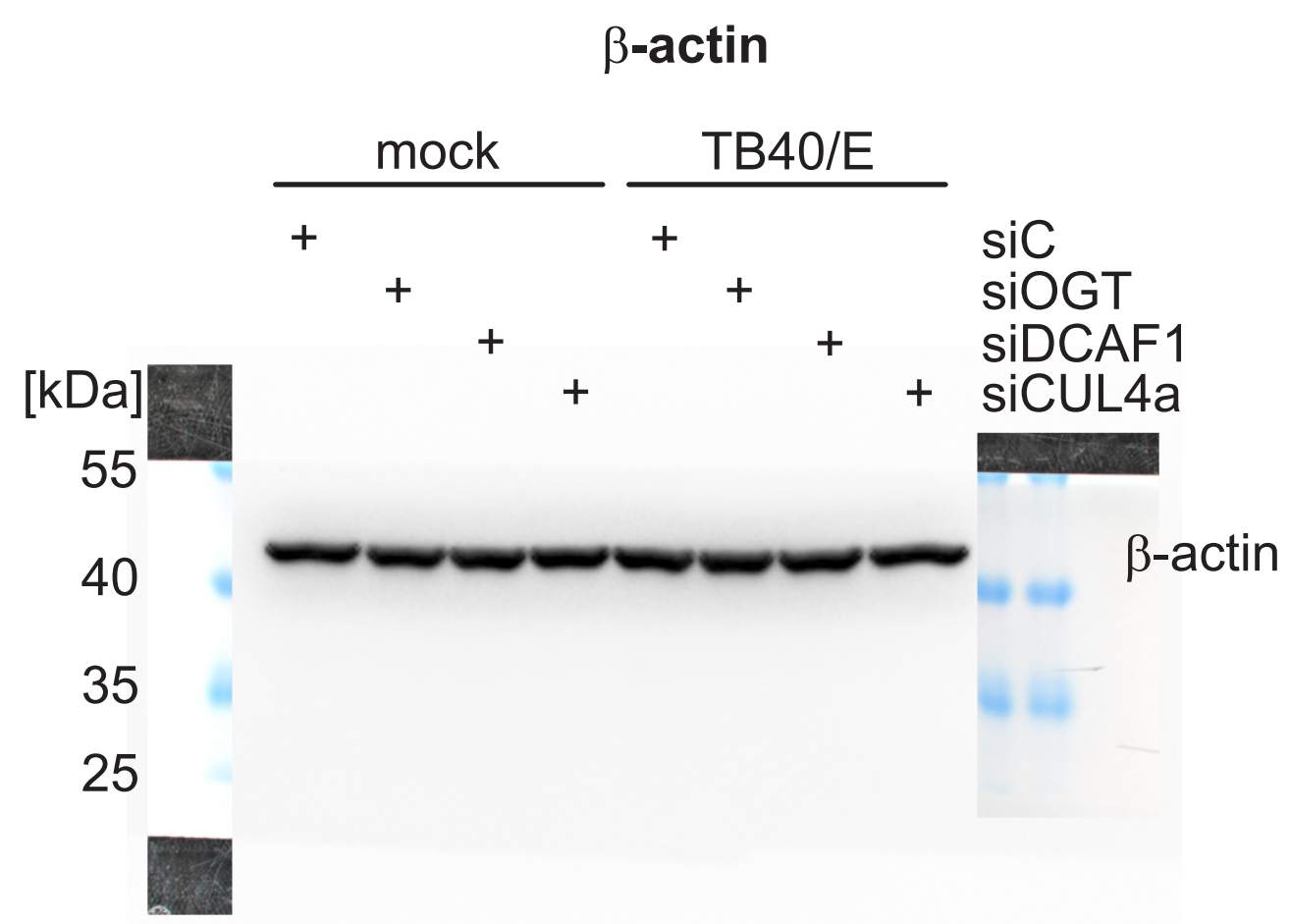

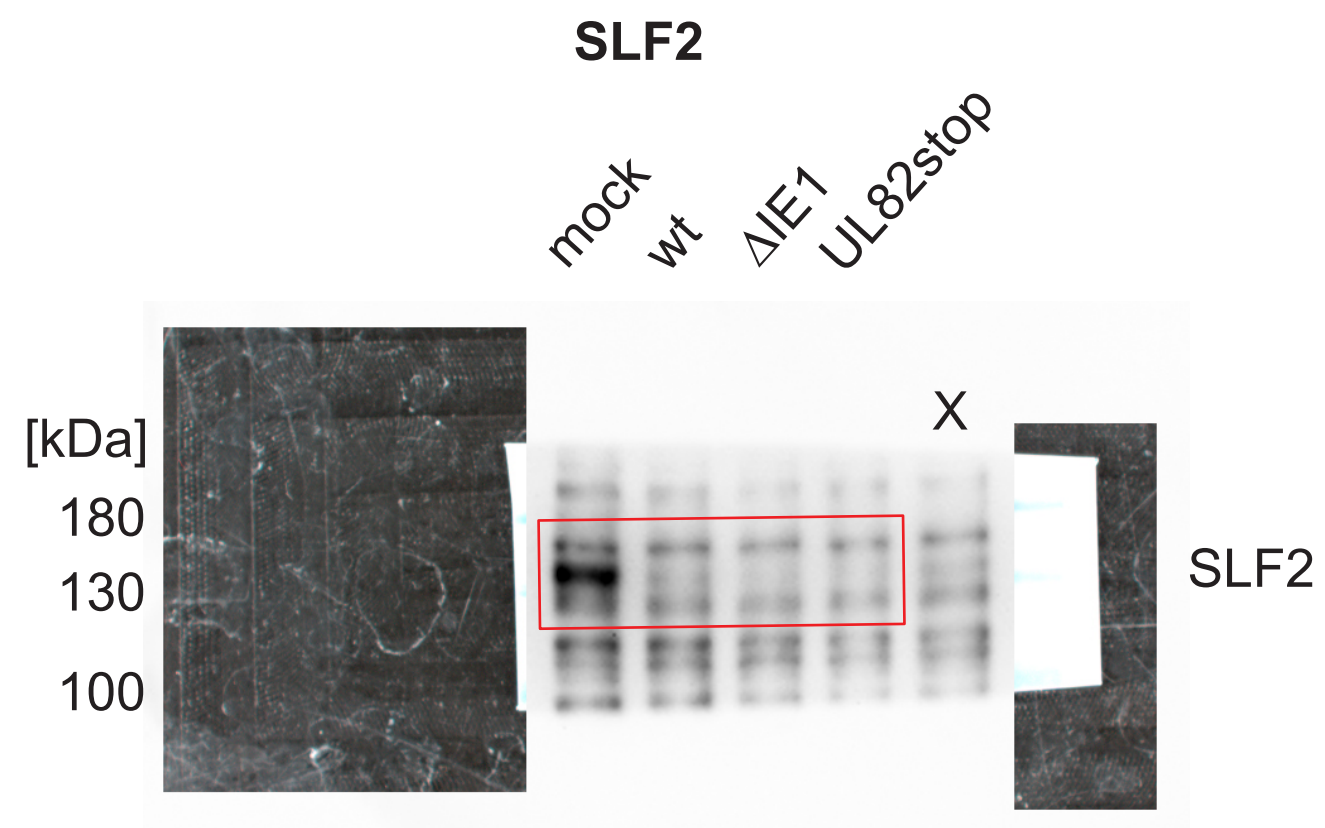

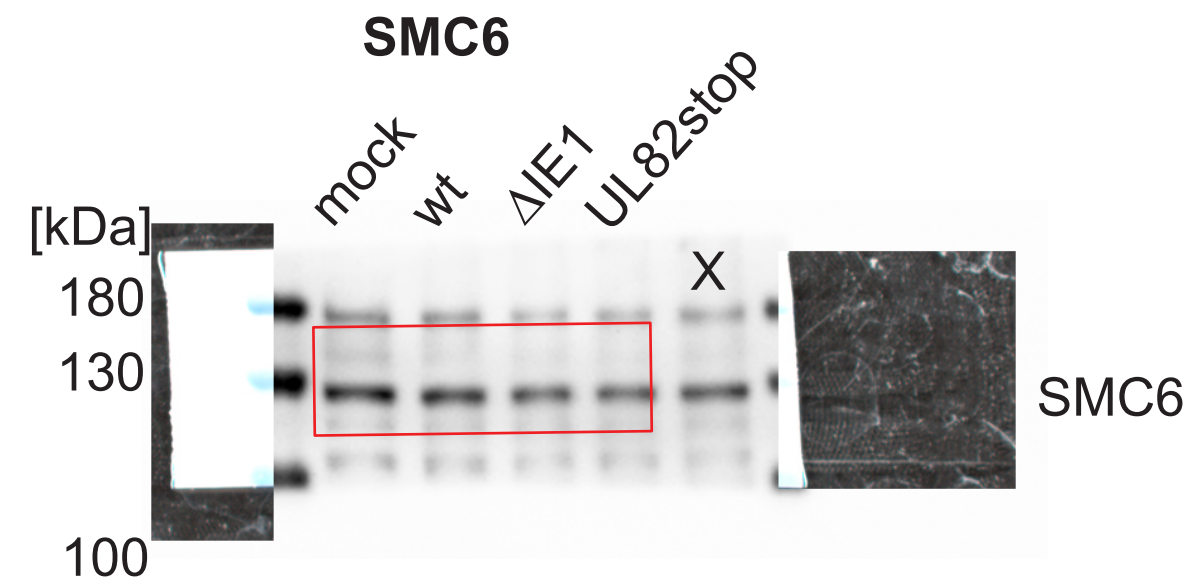

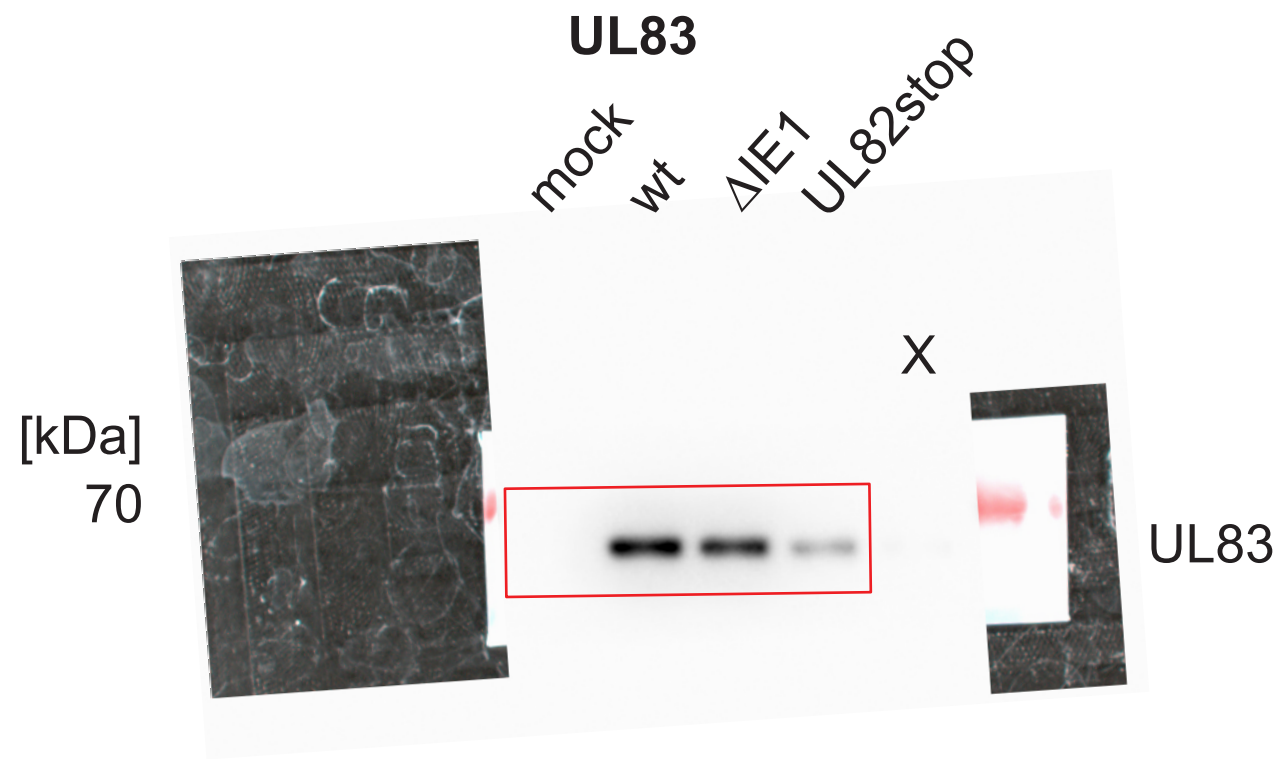

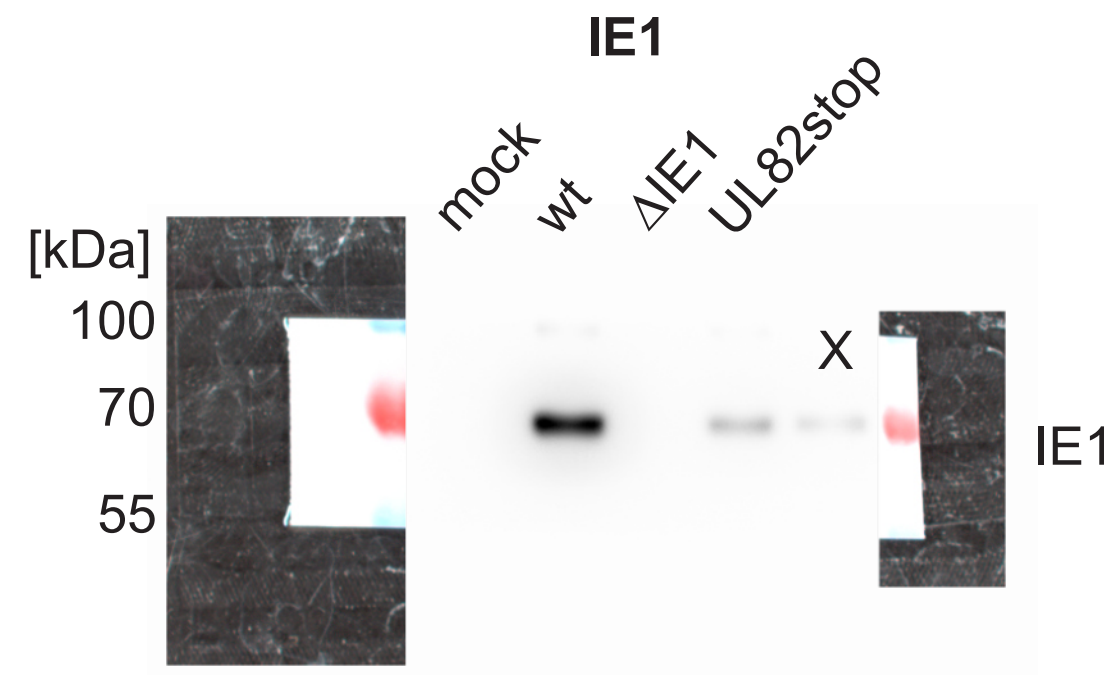

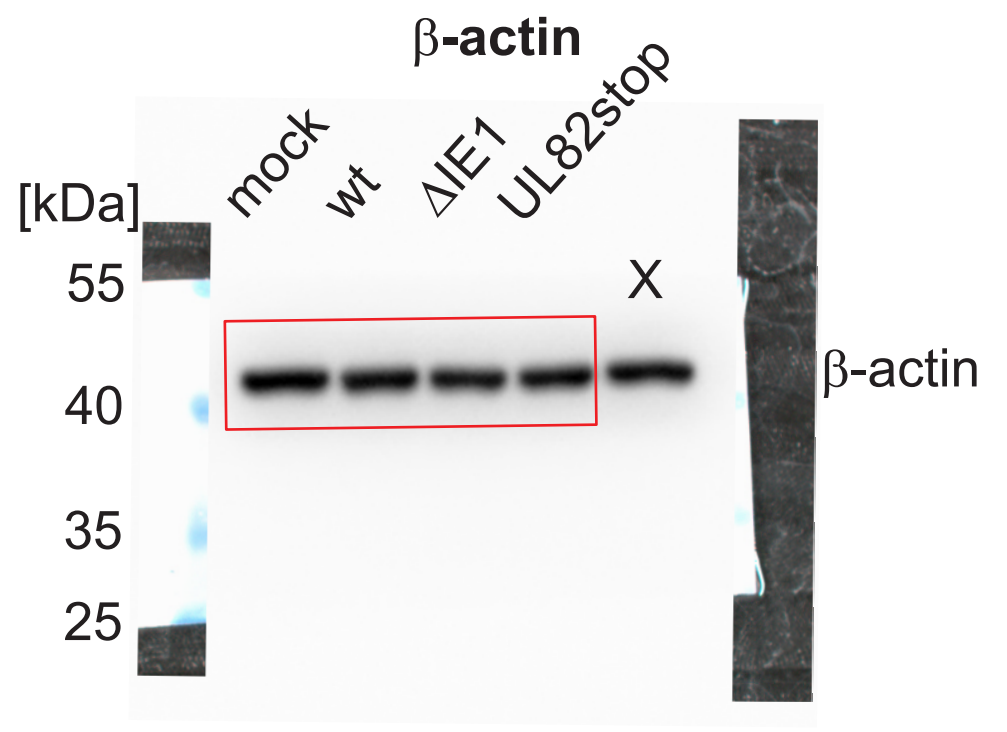

Figure 5C was generated with these images

Detected with: FUSION FX7 imaging system (Vilber Lourmat, Eberhardzell, German)

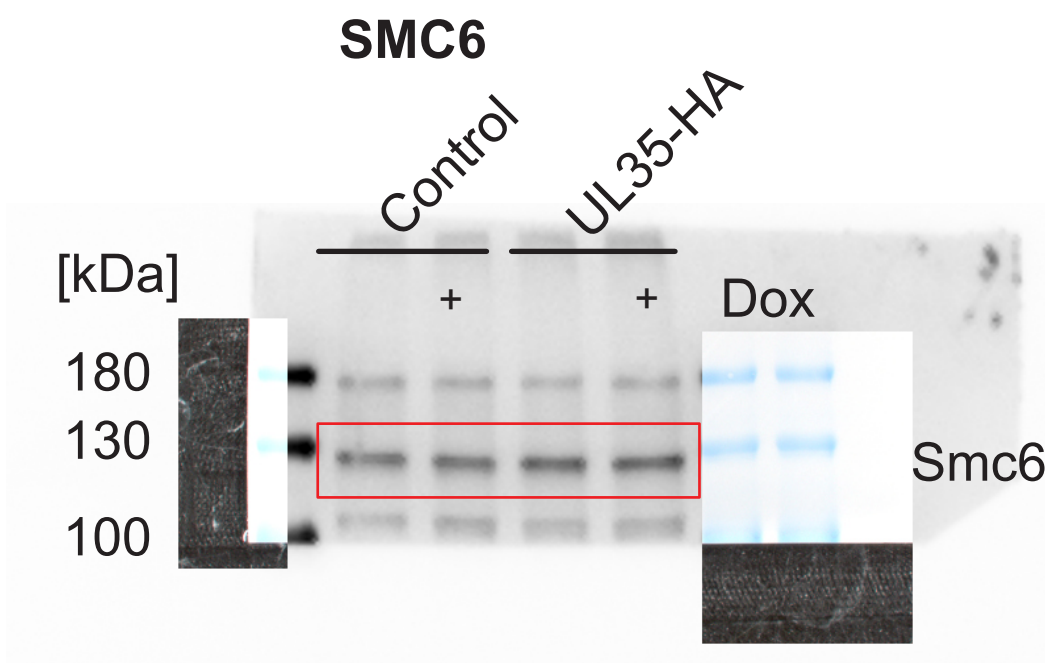

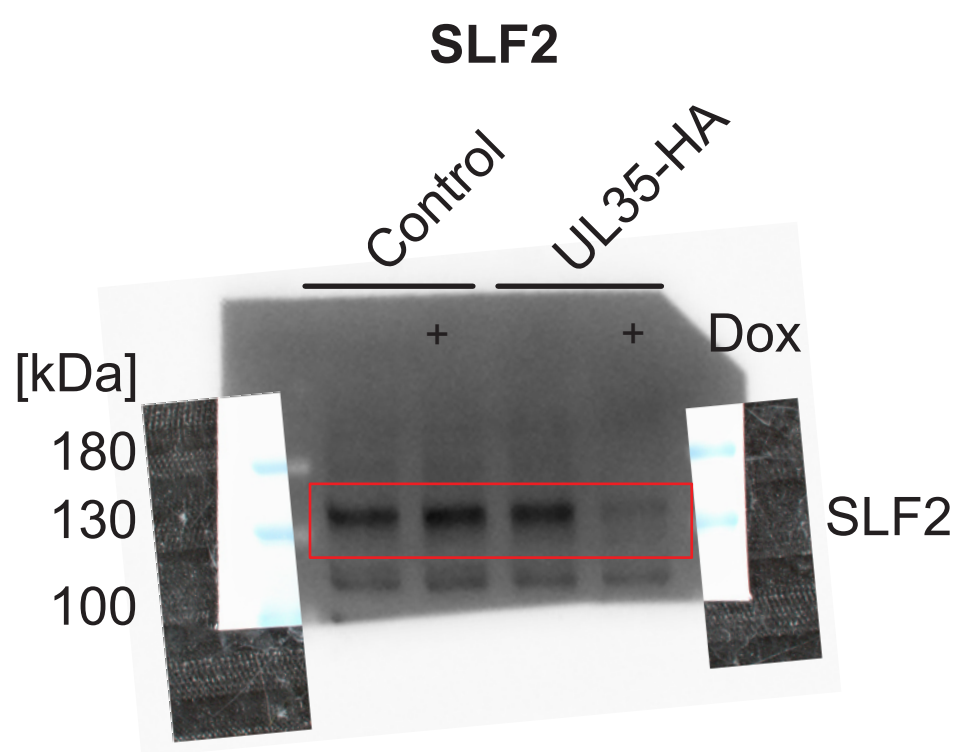

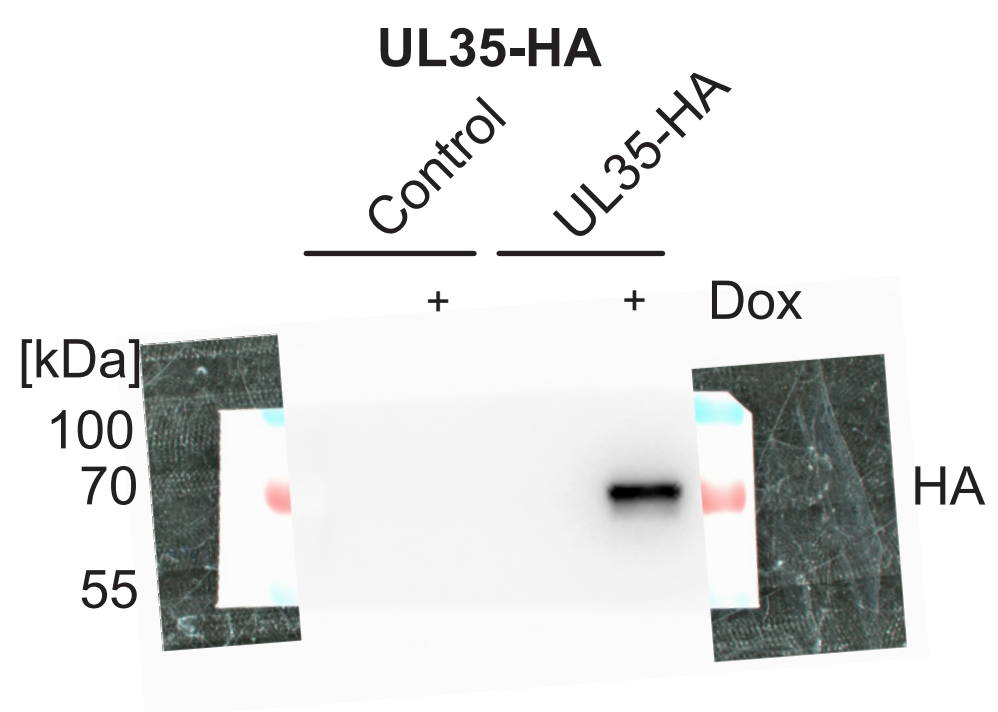

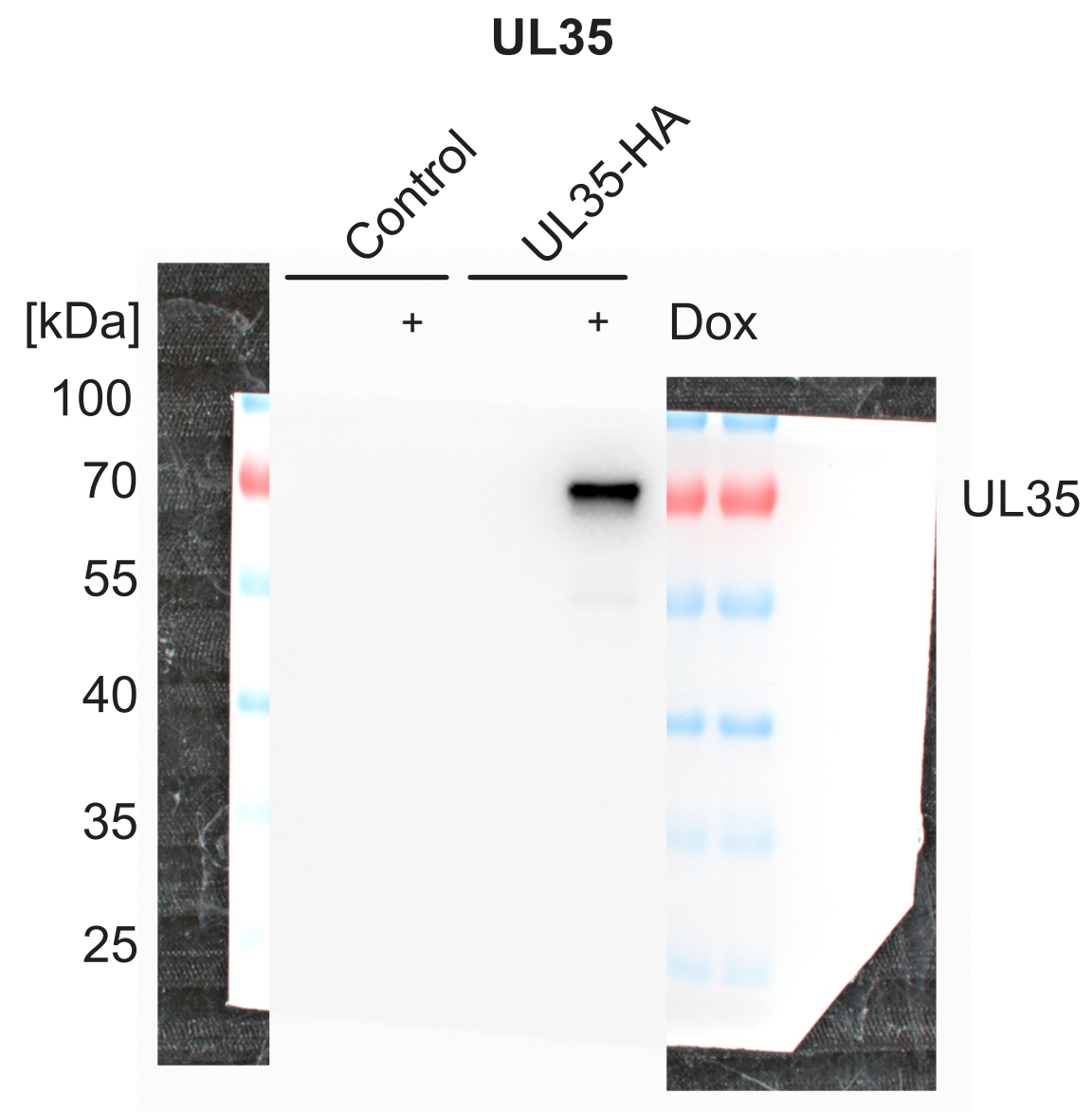

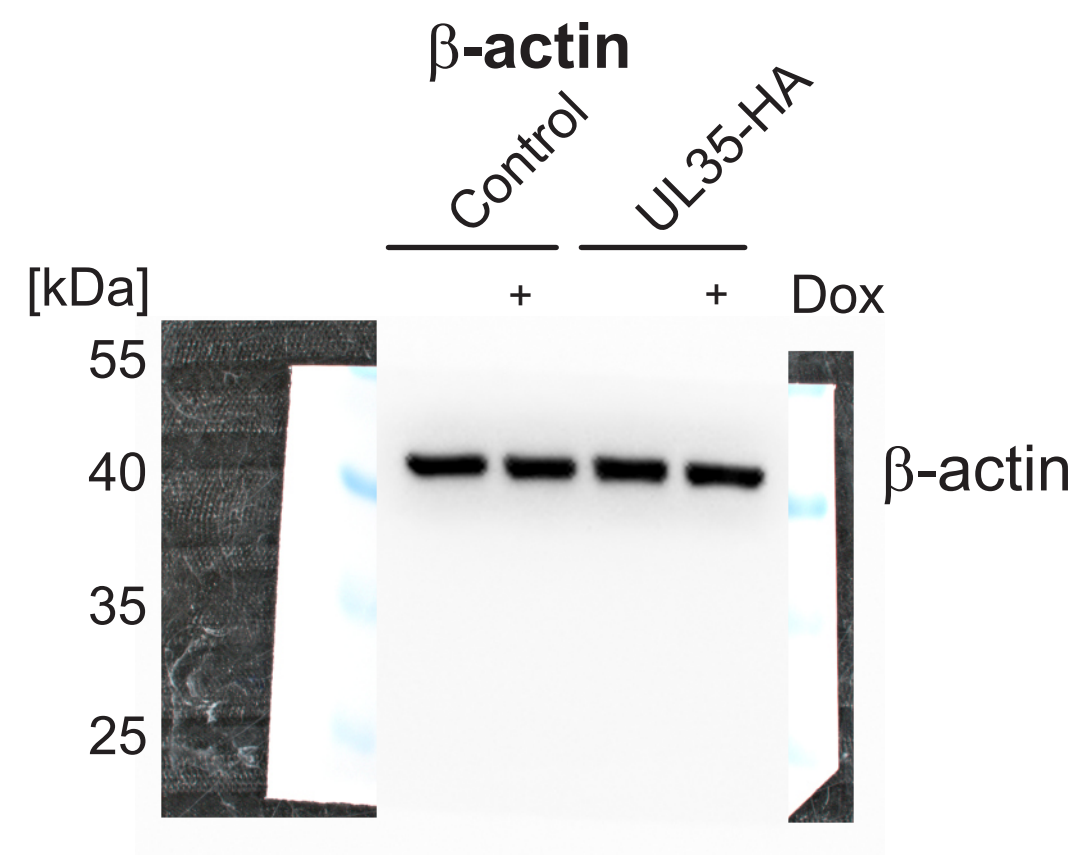

Figure 6C was generated with these images

Detected with: FUSION FX7 imaging system (Vilber Lourmat, Eberhardzell, German)

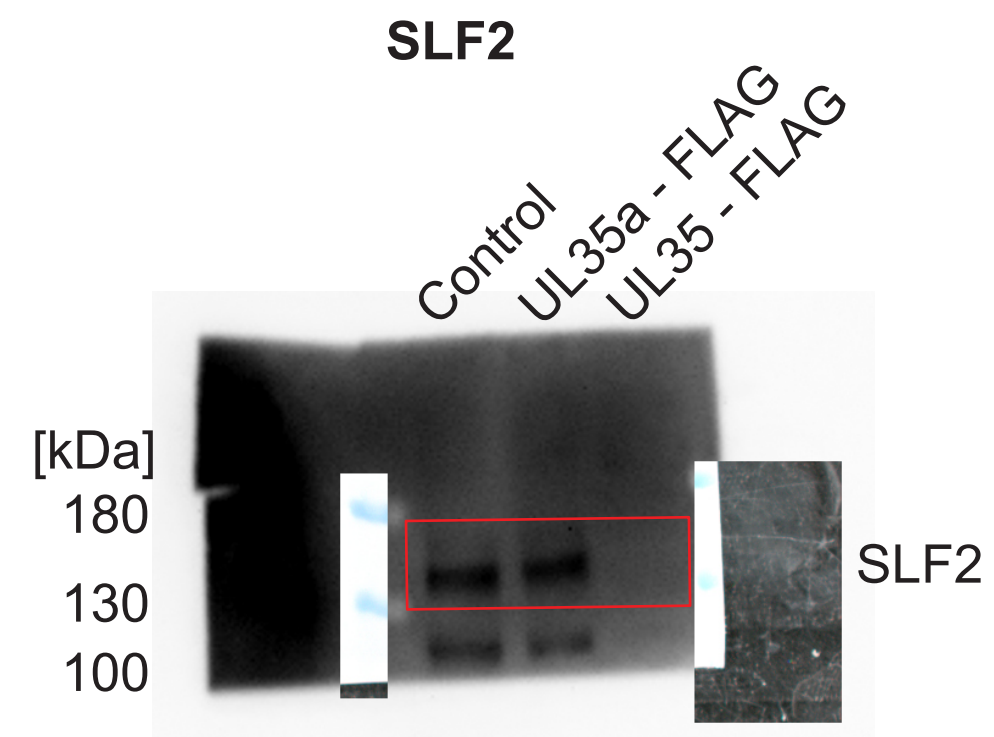

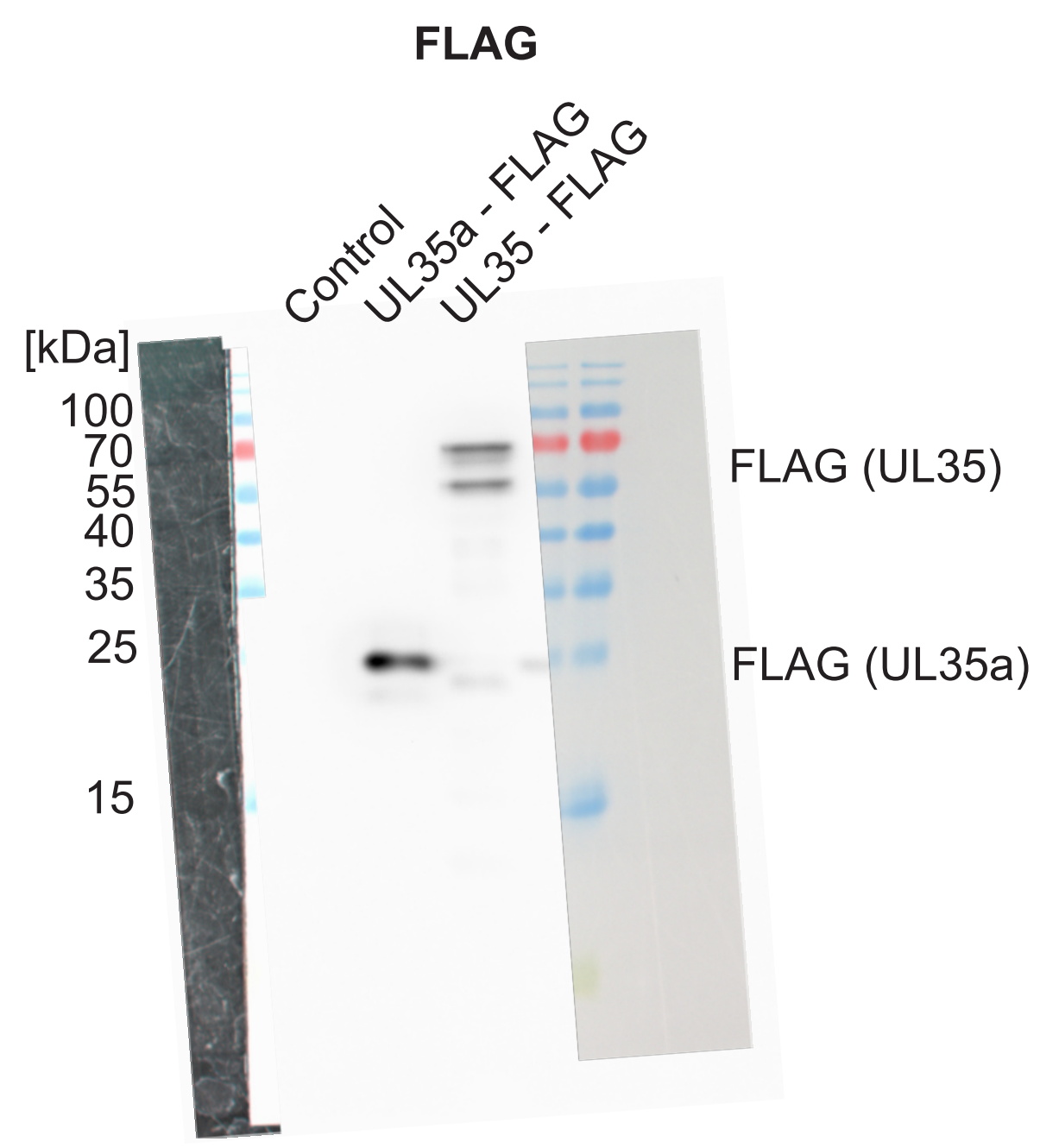

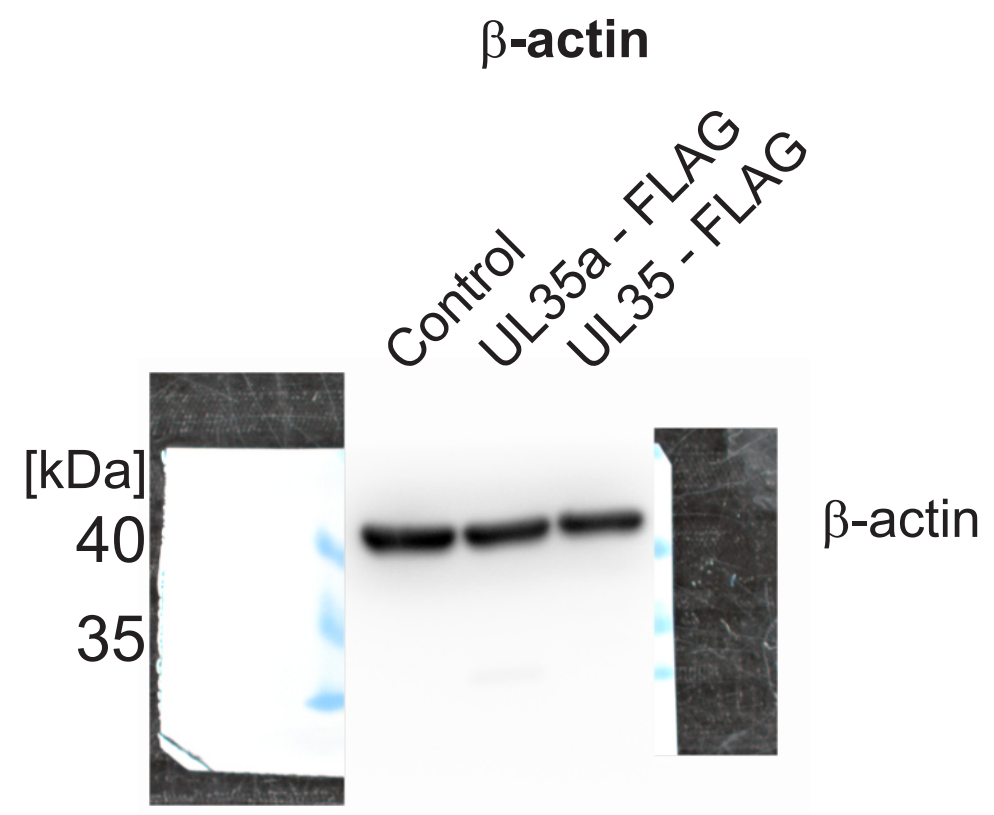

Figure 6D was generated with these images
Detected with: FUSION FX7 imaging system (Vilber Lourmat, Eberhardzell, German)

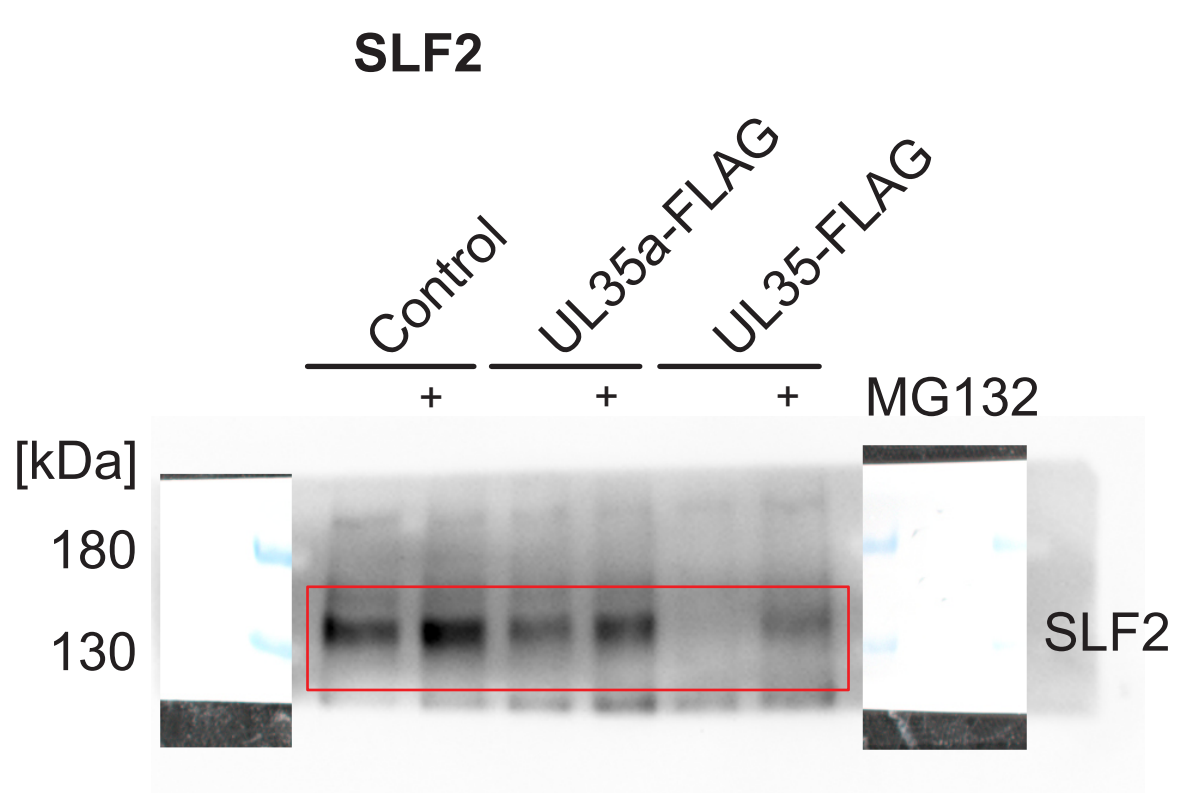

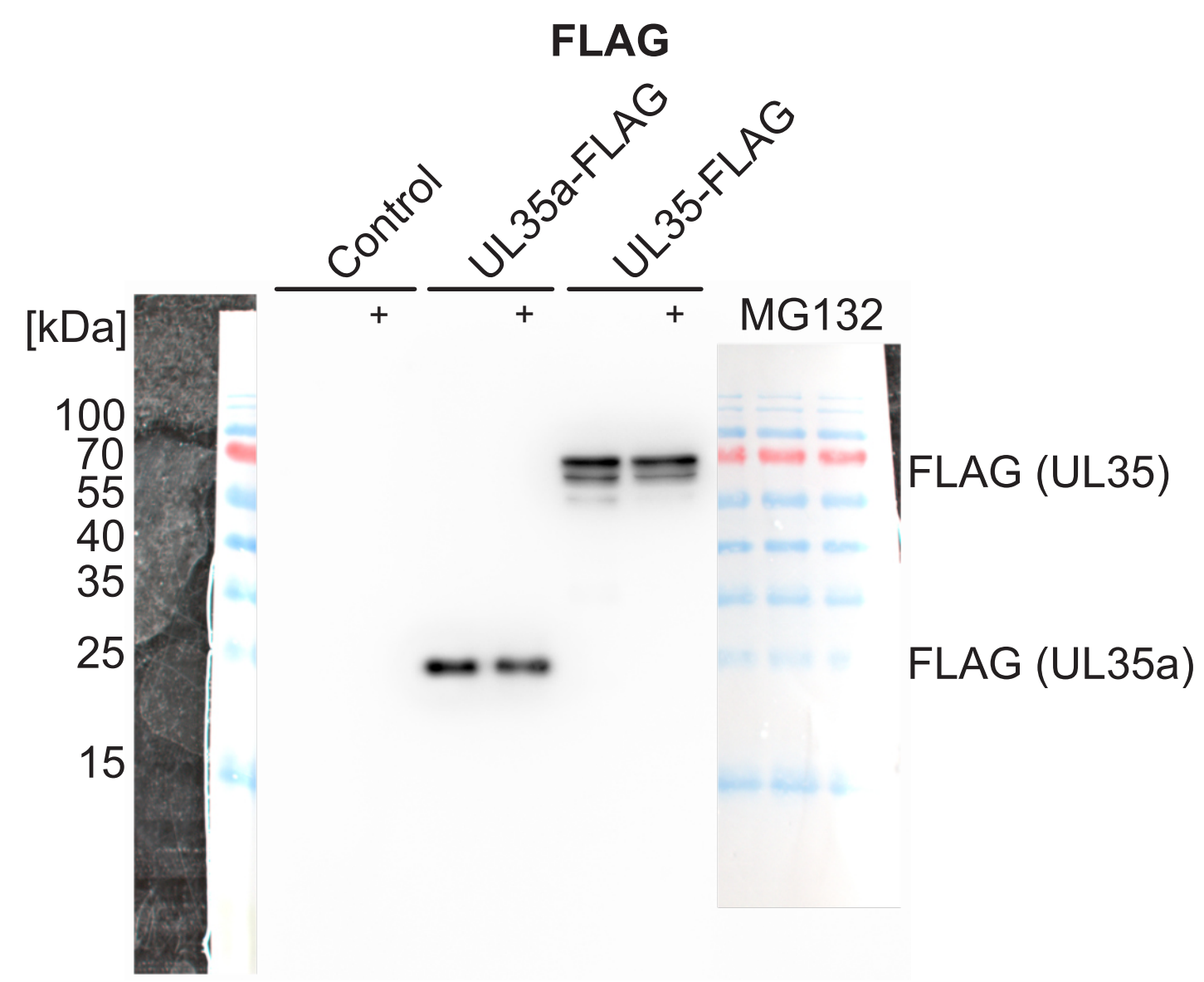

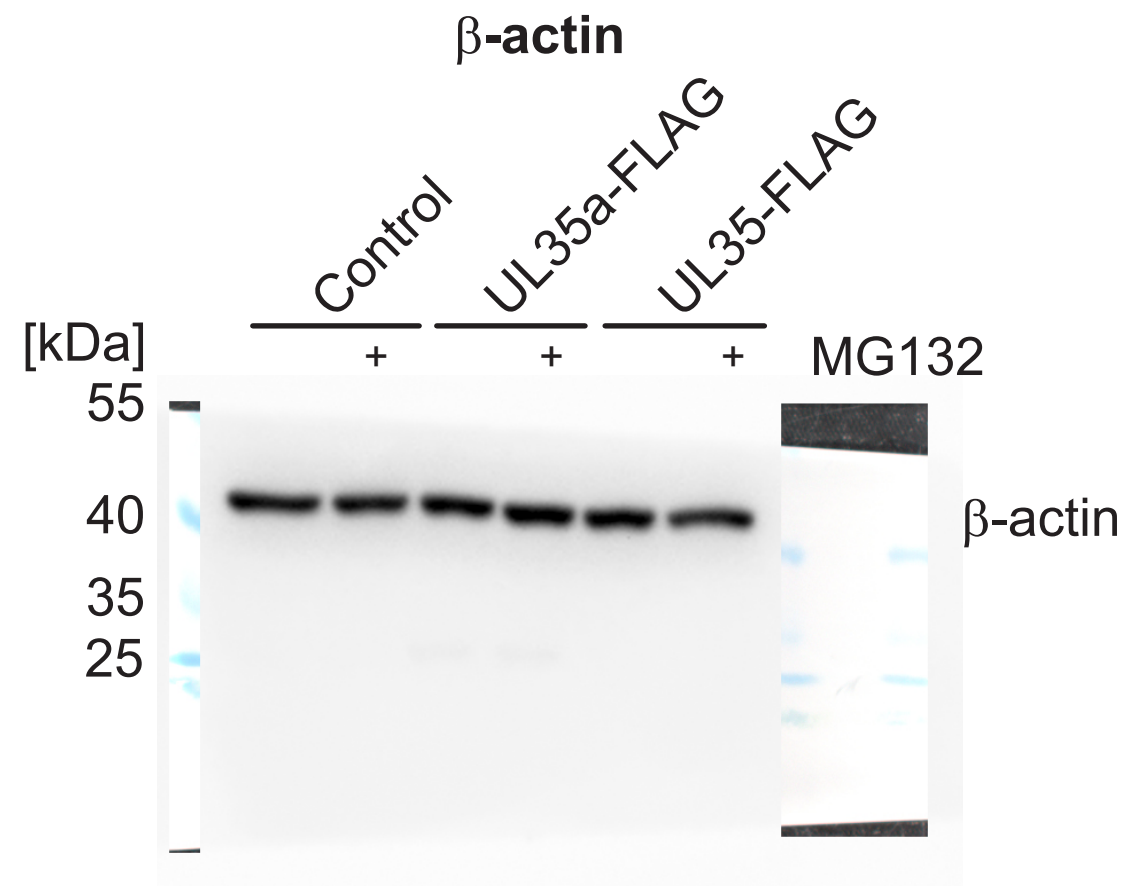

Figure 6F was generated with these images
Detected with: FUSION FX7 imaging system (Vilber Lourmat, Eberhardzell, German)

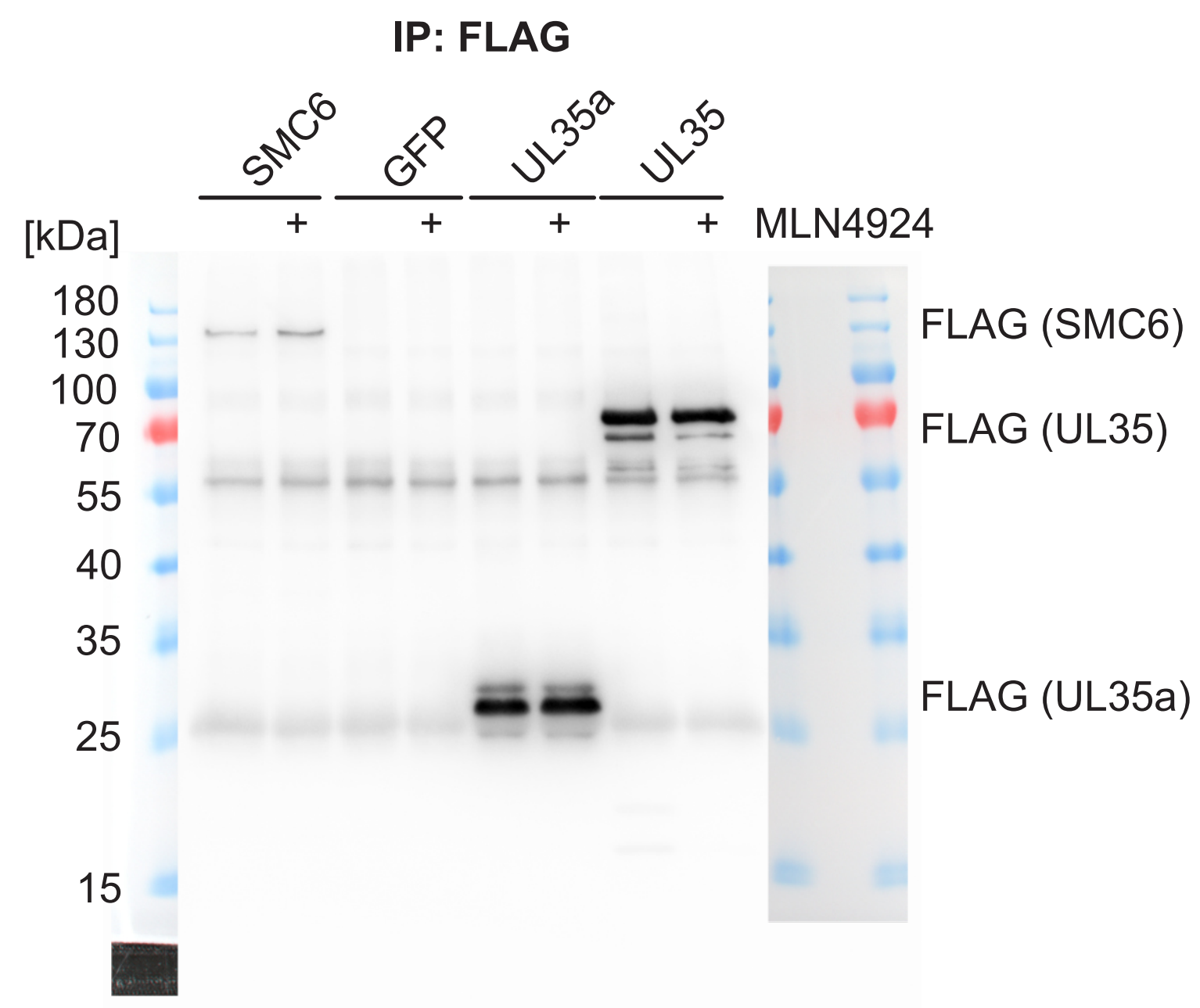

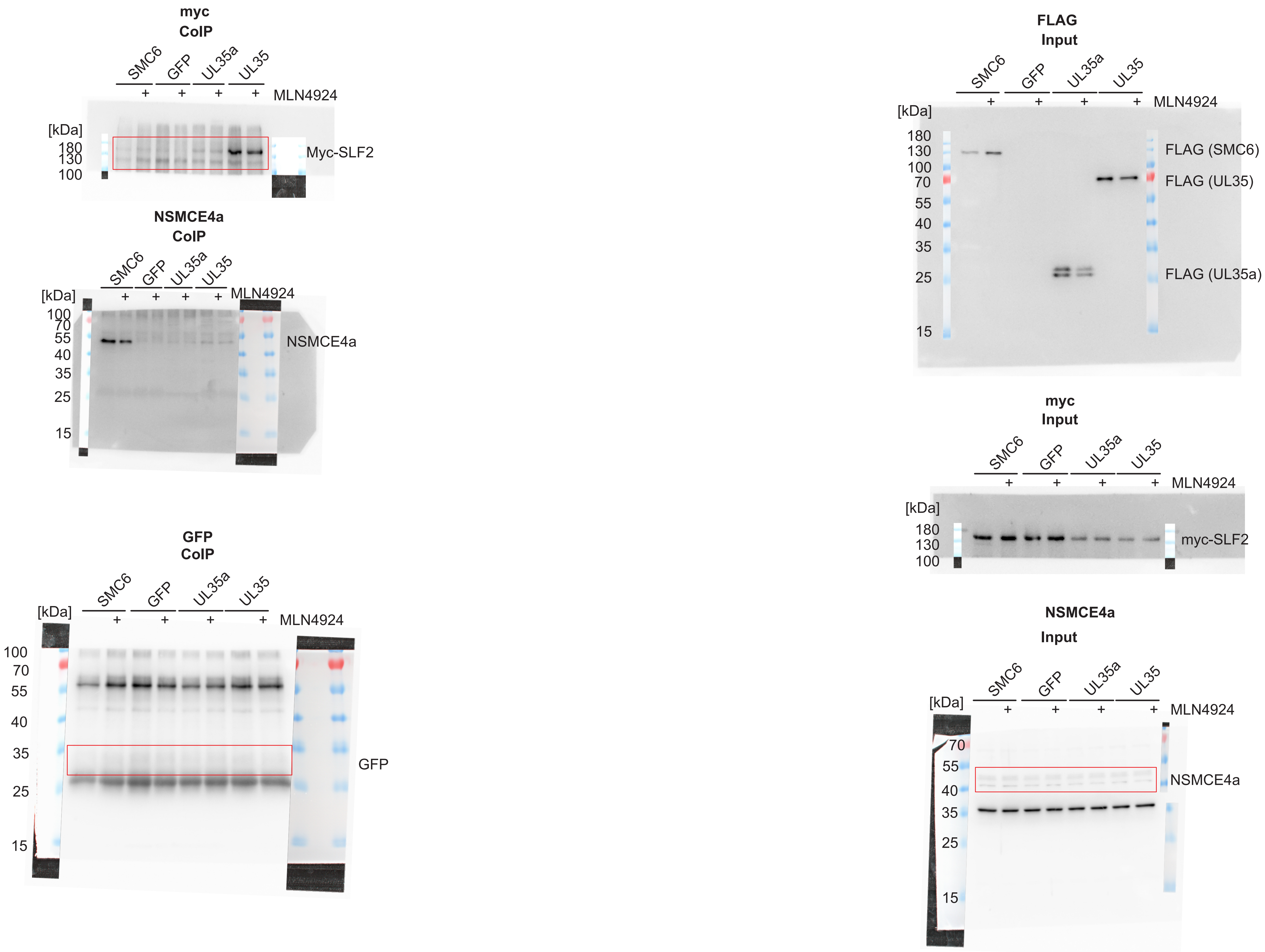
myc
CoIP
SMC6
GFP
UL35a
UL35
+ + + +
MLN4924
[kDa]
180
130
100
Myc-SLF2
NSMCE4a
CoIP
SMC6
GFP
UL35a
UL35
[kDa]
+ + + +
MLN4924
100
70
55
40
35
25
15
NSMCE4a
GFP
CoIP
SMC6
GFP
UL35a
UL35
[kDa]
+ + + +
MLN4924
100
70
55
40
35
25
15
GFP
FLAG
Input
SMC6
GFP
UL35a
UL35
+ + + +
MLN4924
[kDa]
180
130
100
70
55
40
35
25
15
FLAG (SMC6)
FLAG (UL35)
FLAG (UL35a)
myc
Input
SMC6
GFP
UL35a
UL35
+ + + +
MLN4924
[kDa]
180
130
100
myc-SLF2
NSMCE4a
Input
SMC6
GFP
UL35a
UL35
[kDa]
+ + + +
MLN4924
70
55
40
35
25
15
NSMCE4a

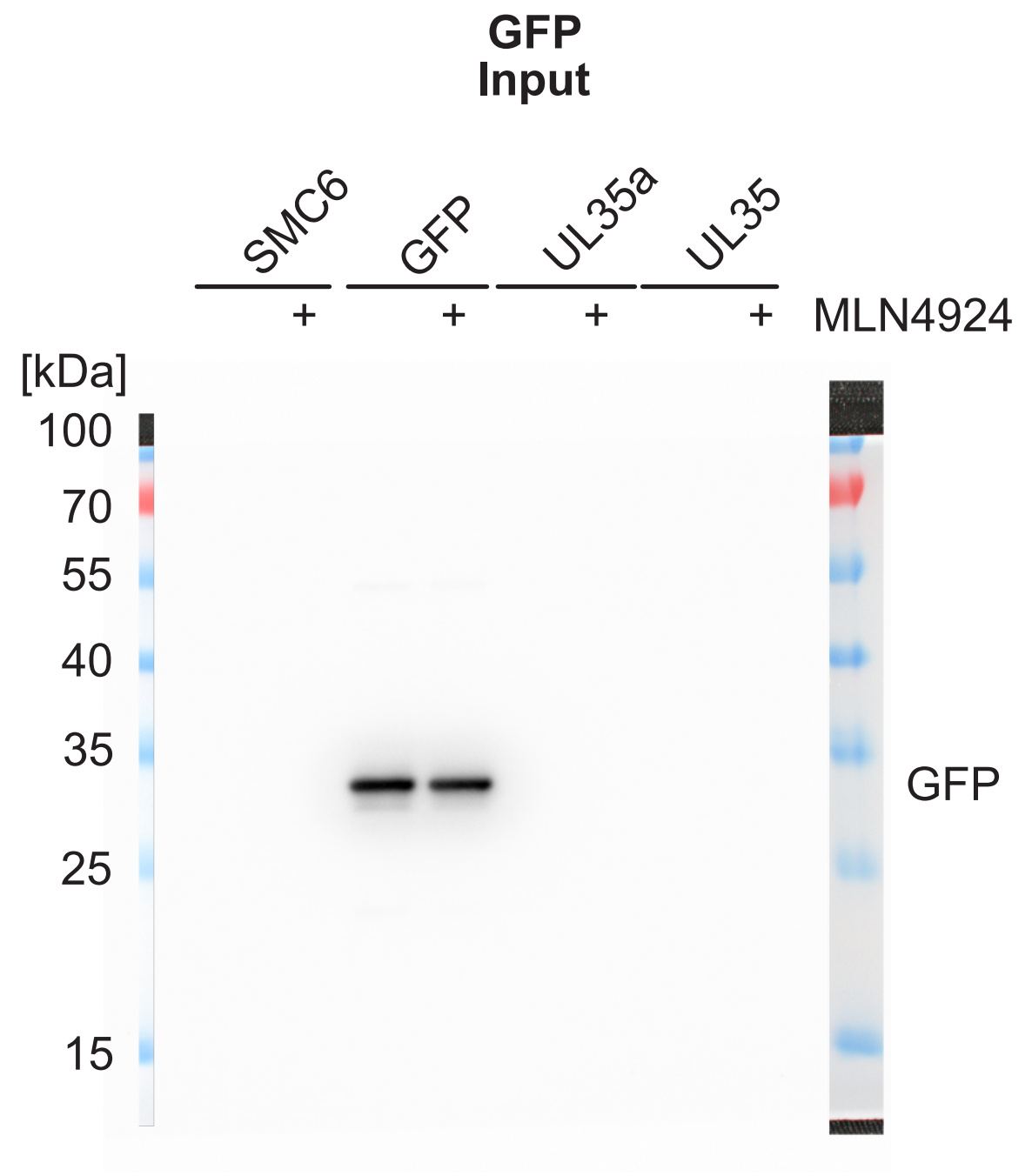

Figure 7A was generated with these images
Detected with: FUSION FX7 imaging system (Vilber Lourmat, Eberhardzell, German)

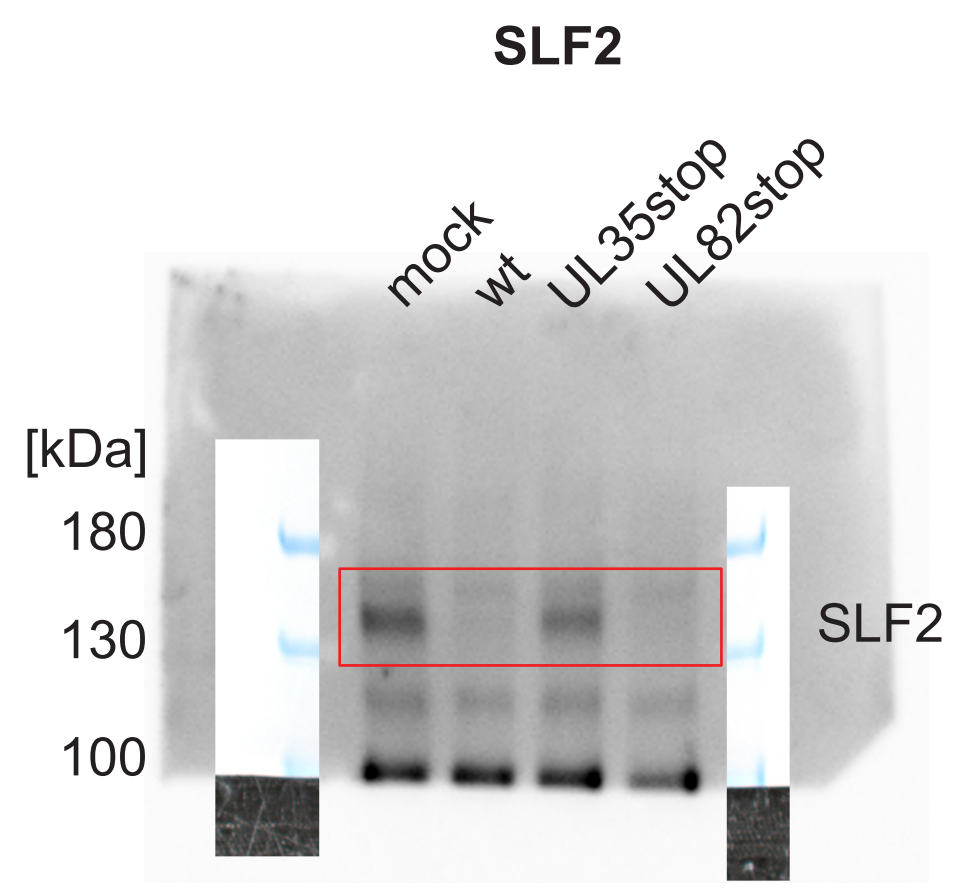

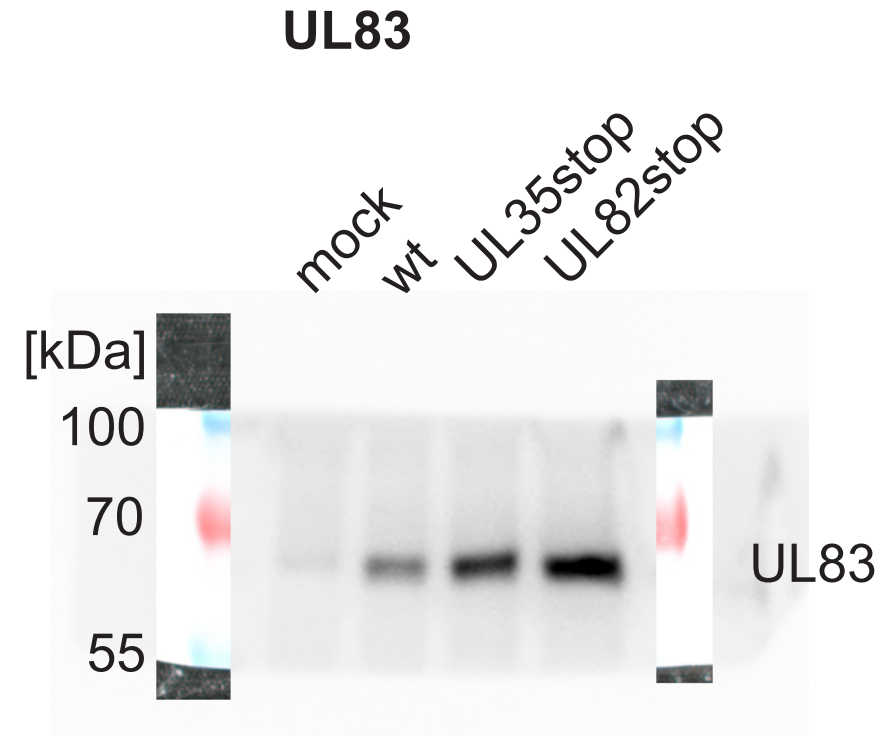

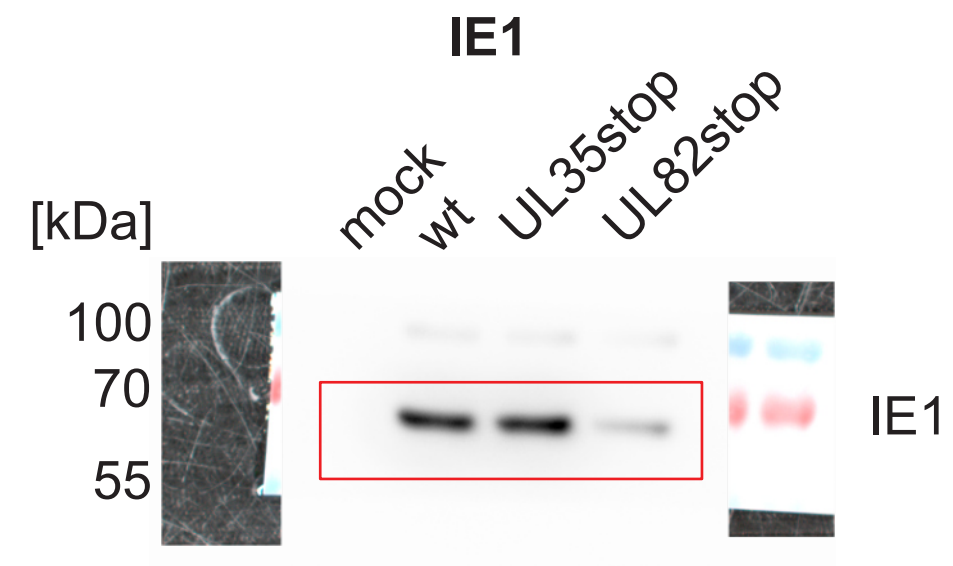

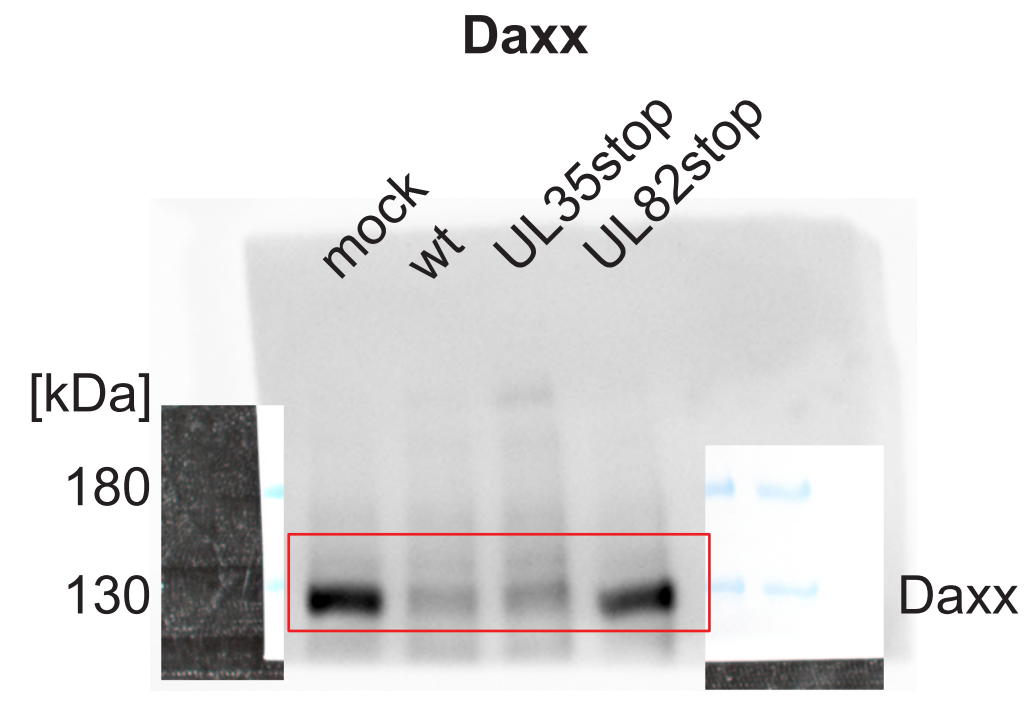

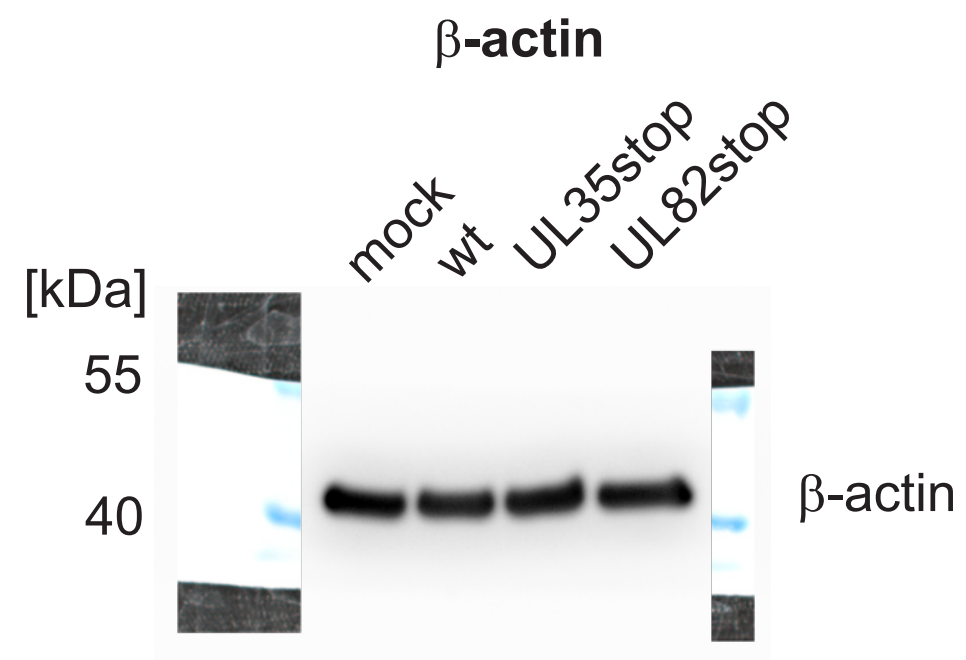

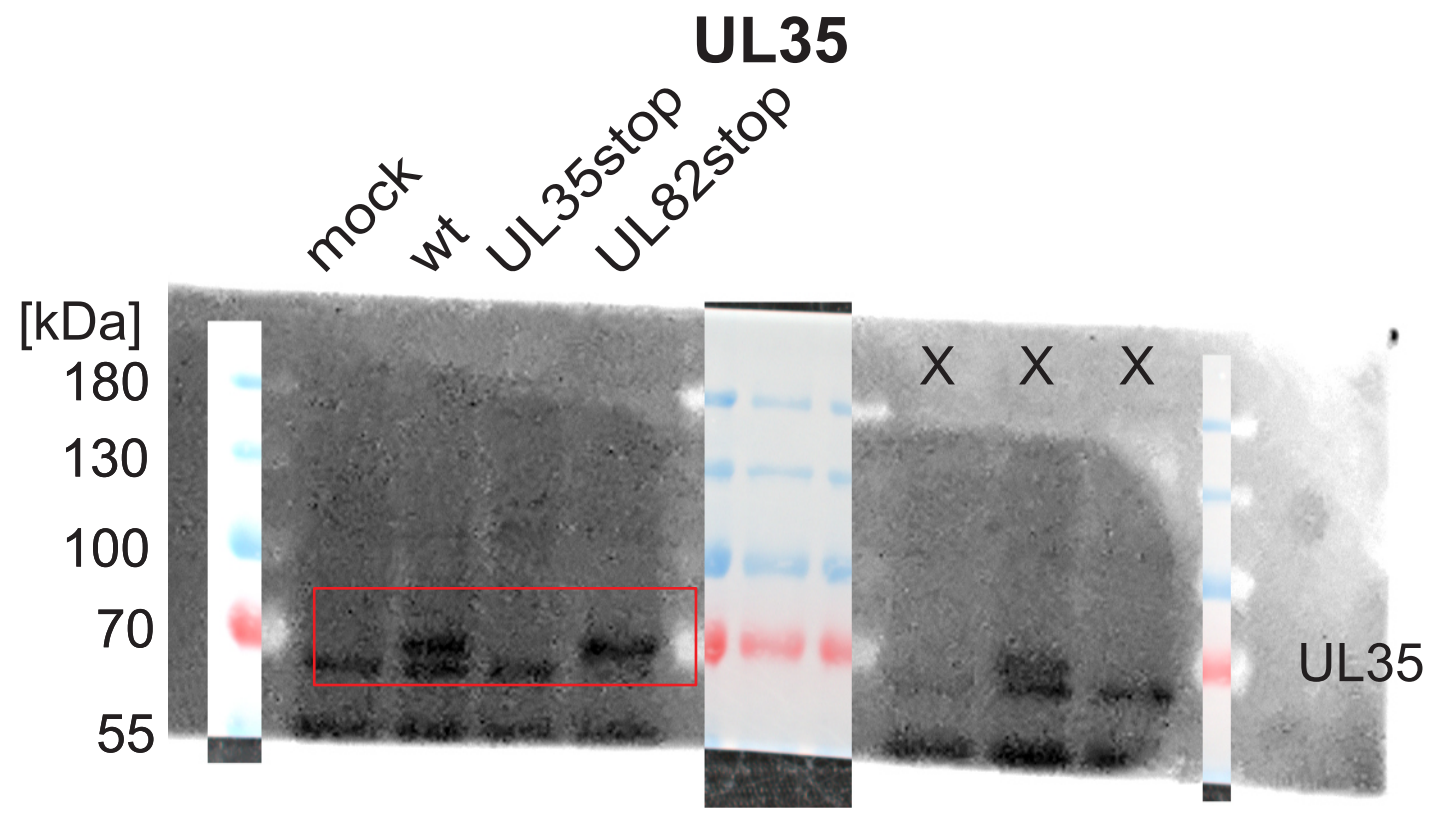

Figure **Supplementary 1A** was generated with these images
Detected with: FUSION FX7 imaging system (Vilber Lourmat, Eberhardzell, German)

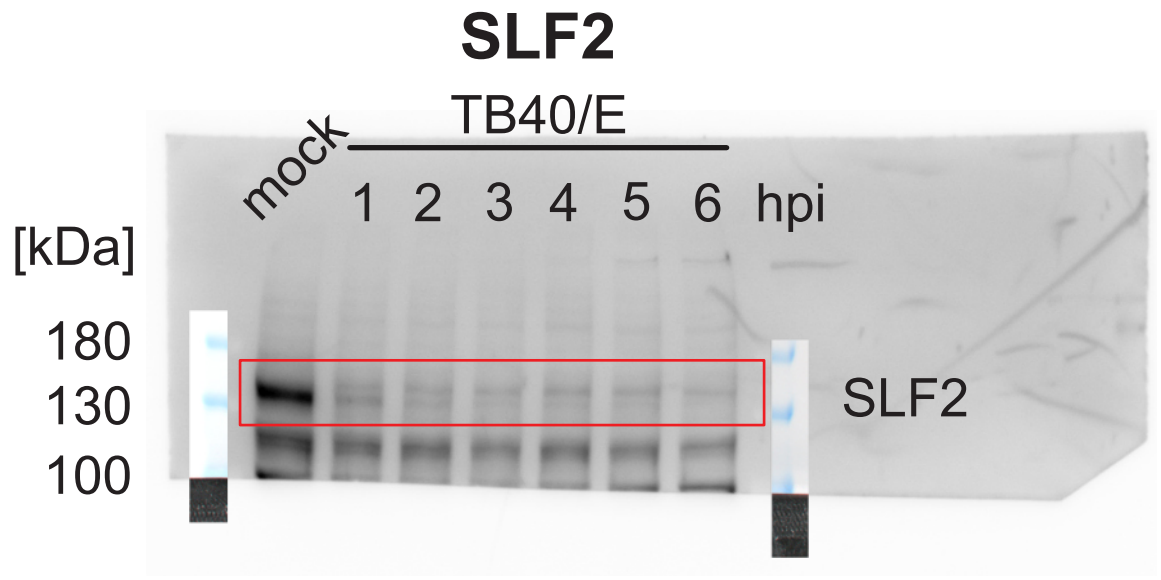

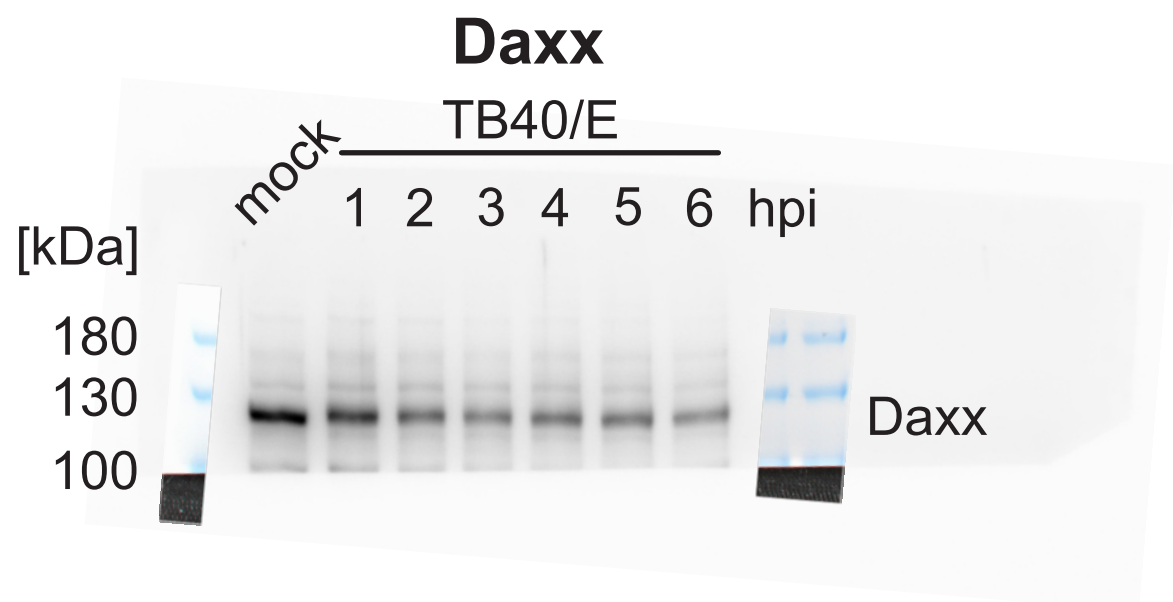

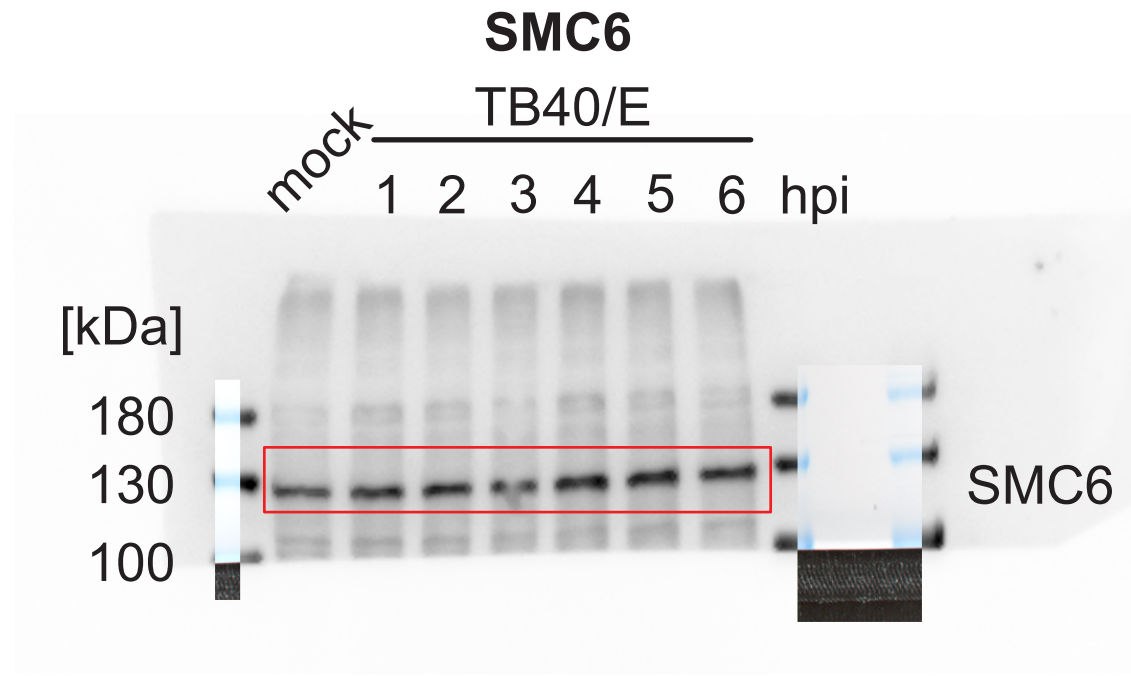

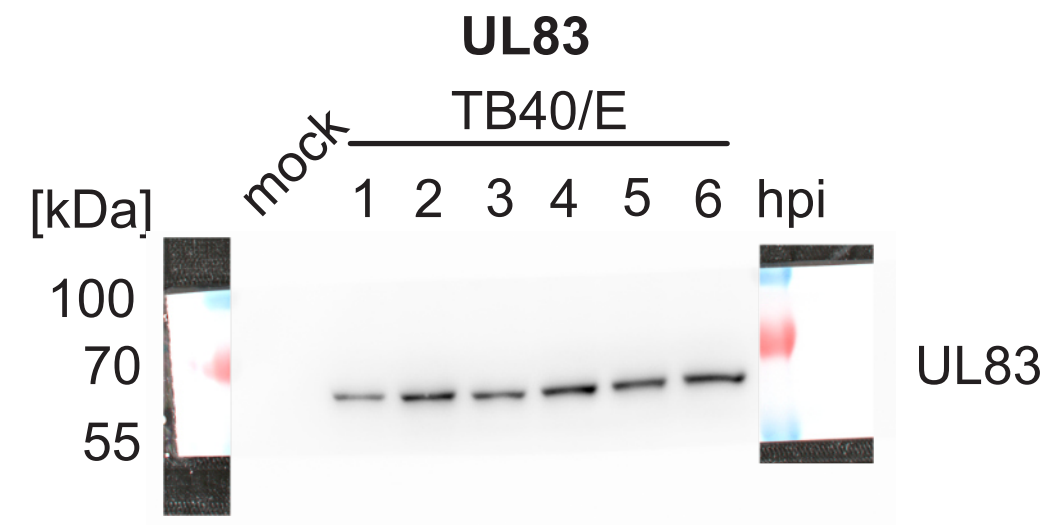

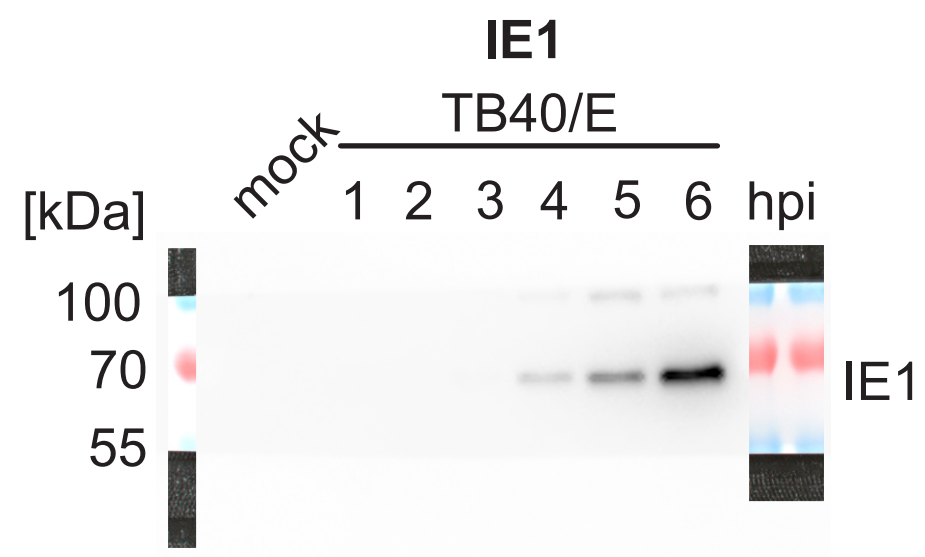

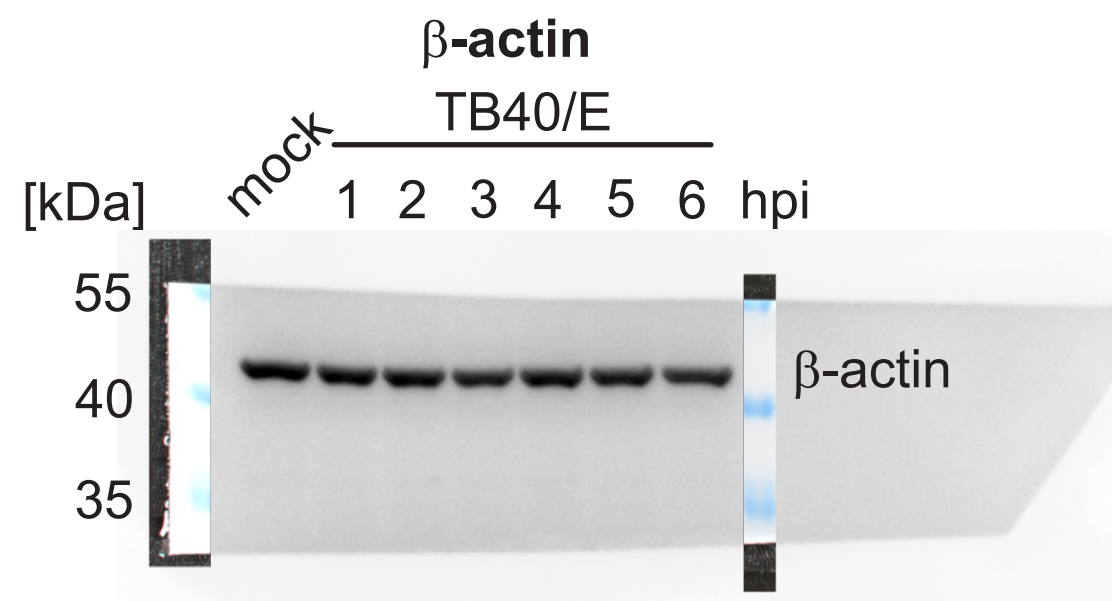

Figure **Supplementary 1B** was generated with these images
Detected with: FUSION FX7 imaging system (Vilber Lourmat, Eberhardzell, German)

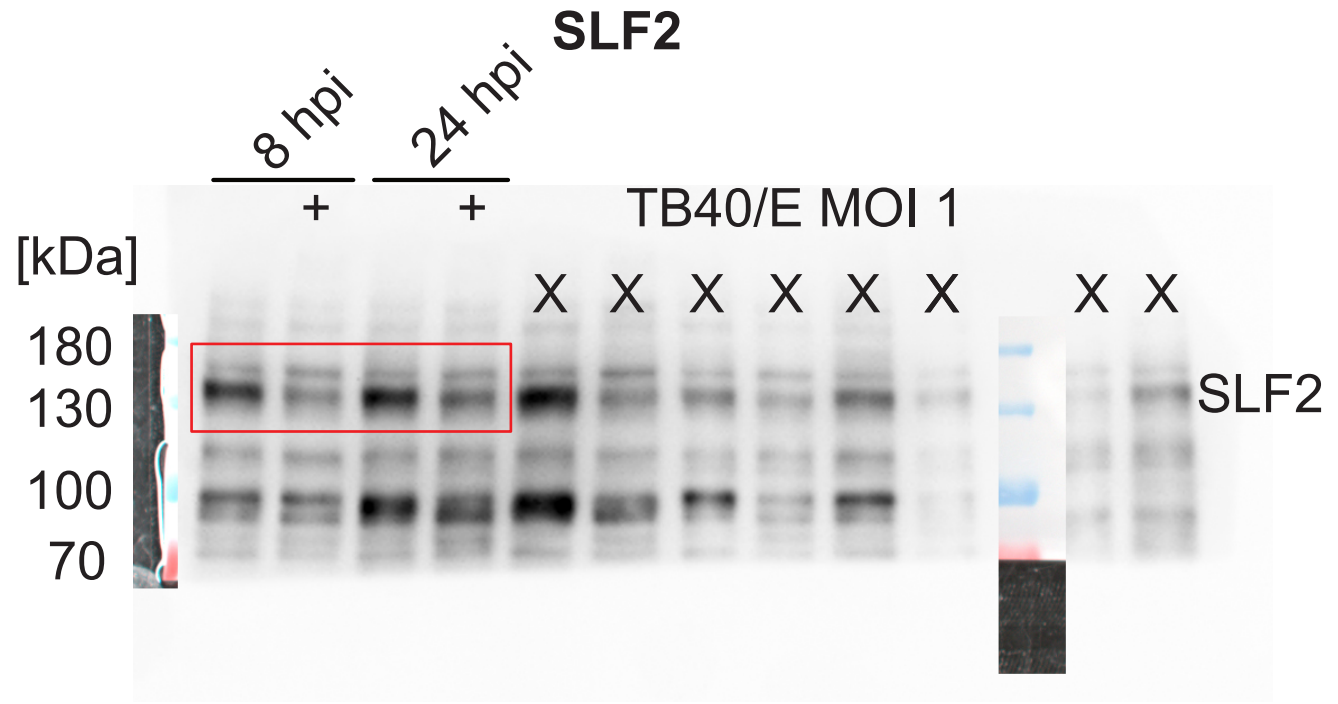

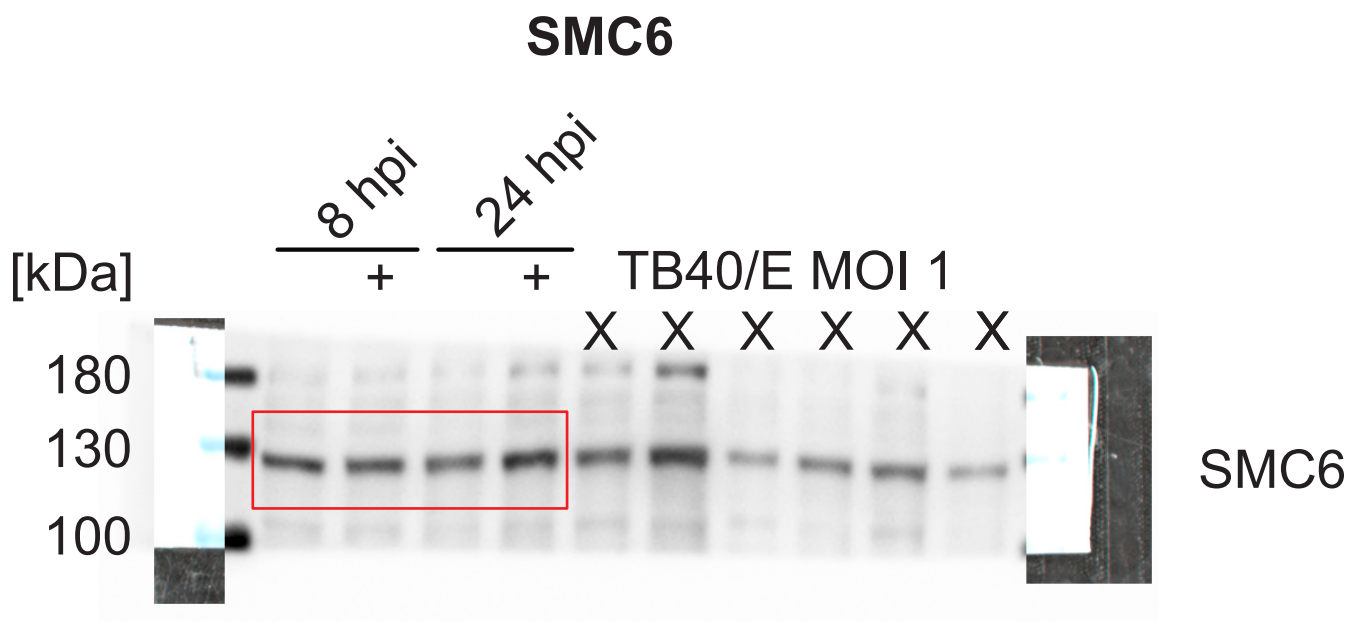

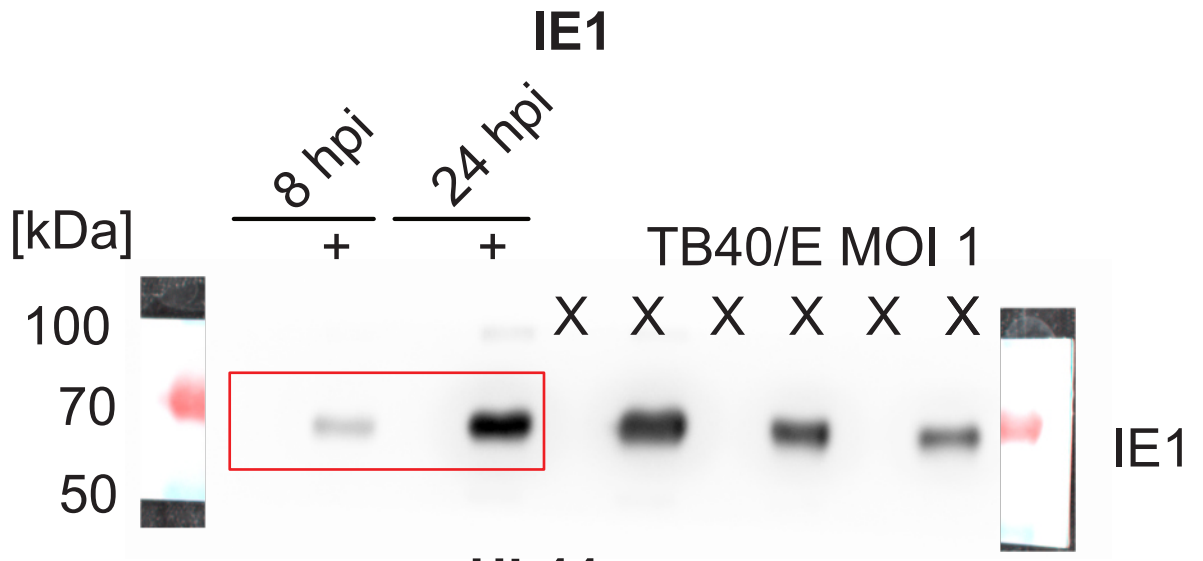

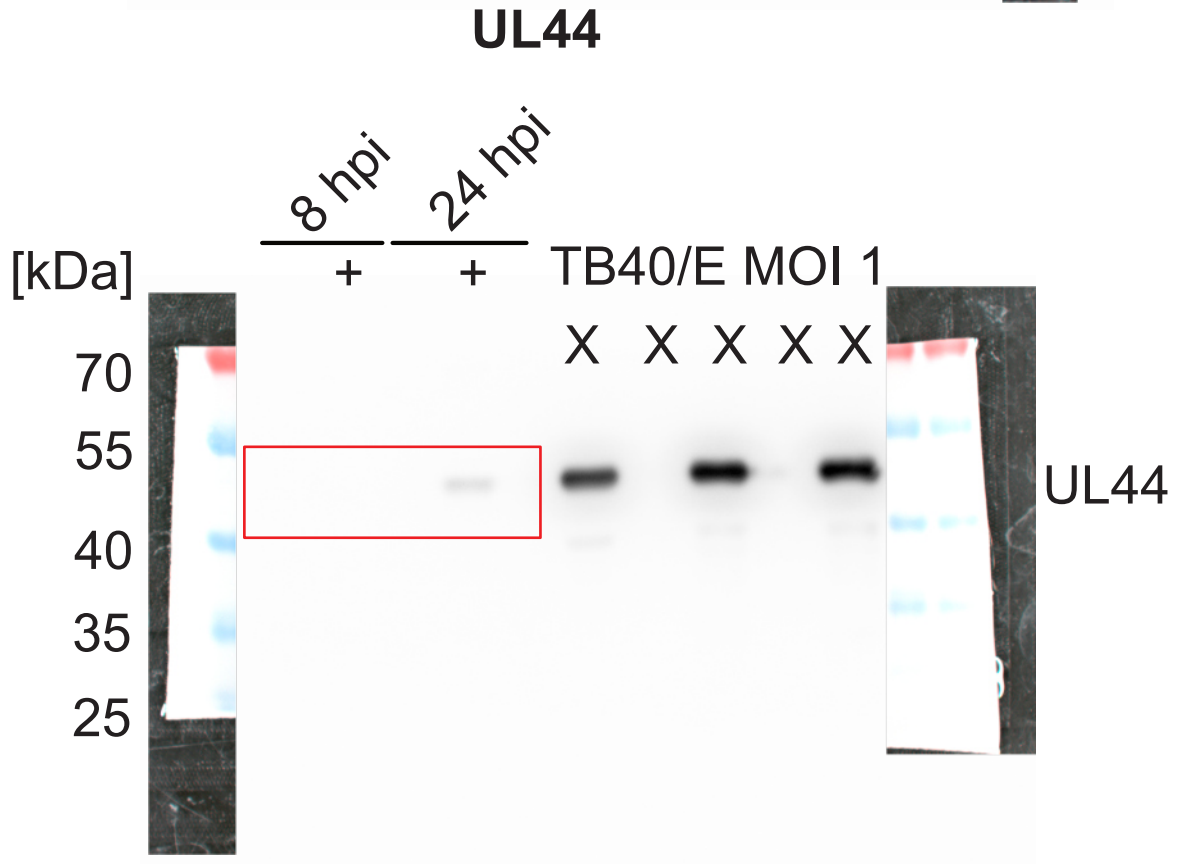

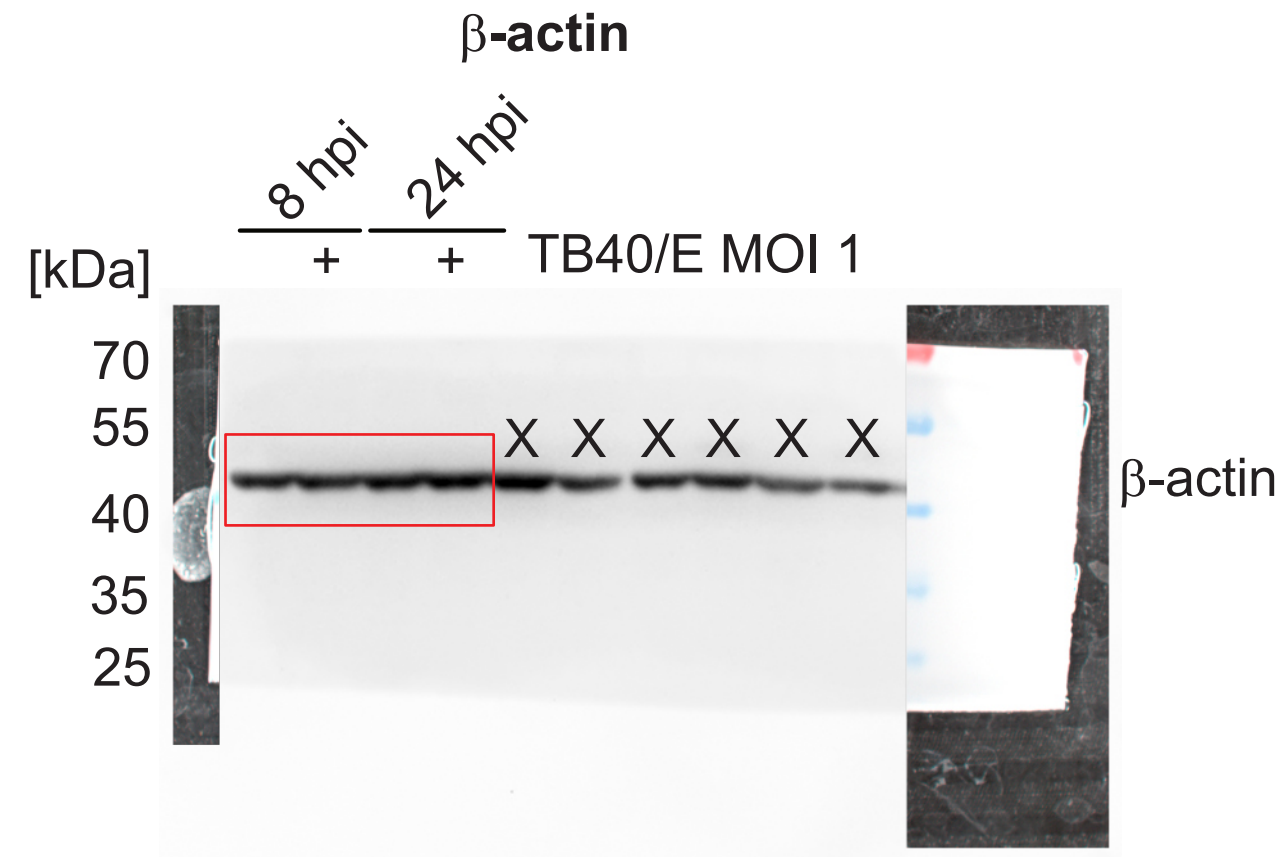

Figure **Supplementary 3A** was generated with these images
Detected with: FUSION FX7 imaging system (Vilber Lourmat, Eberhardzell, German)

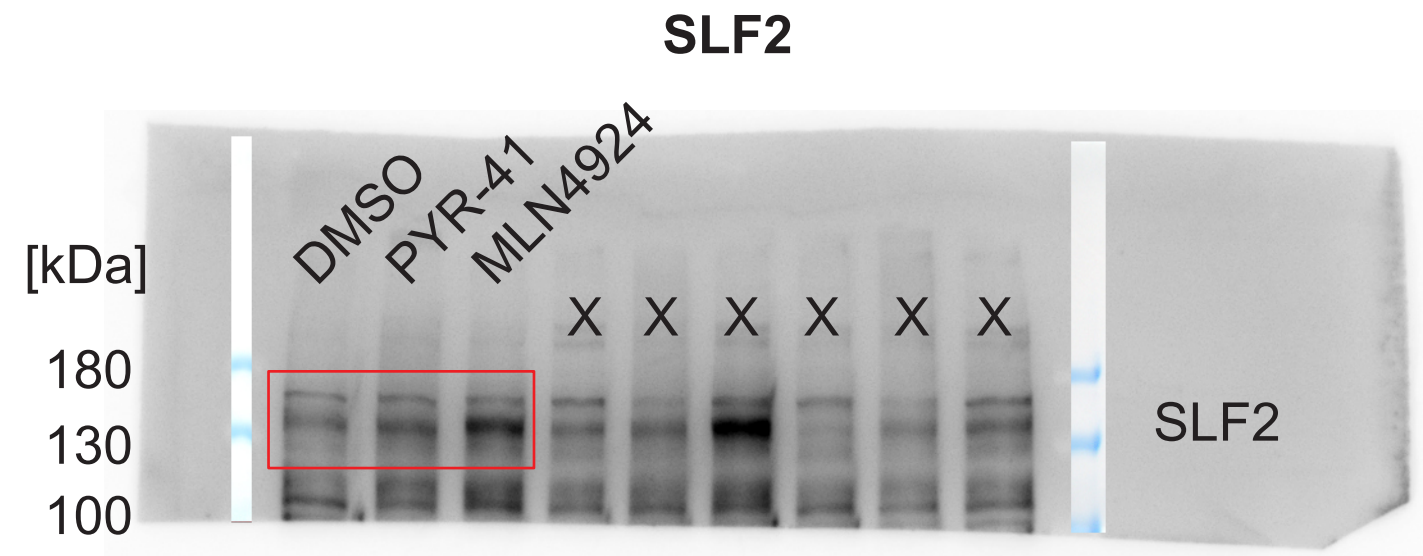

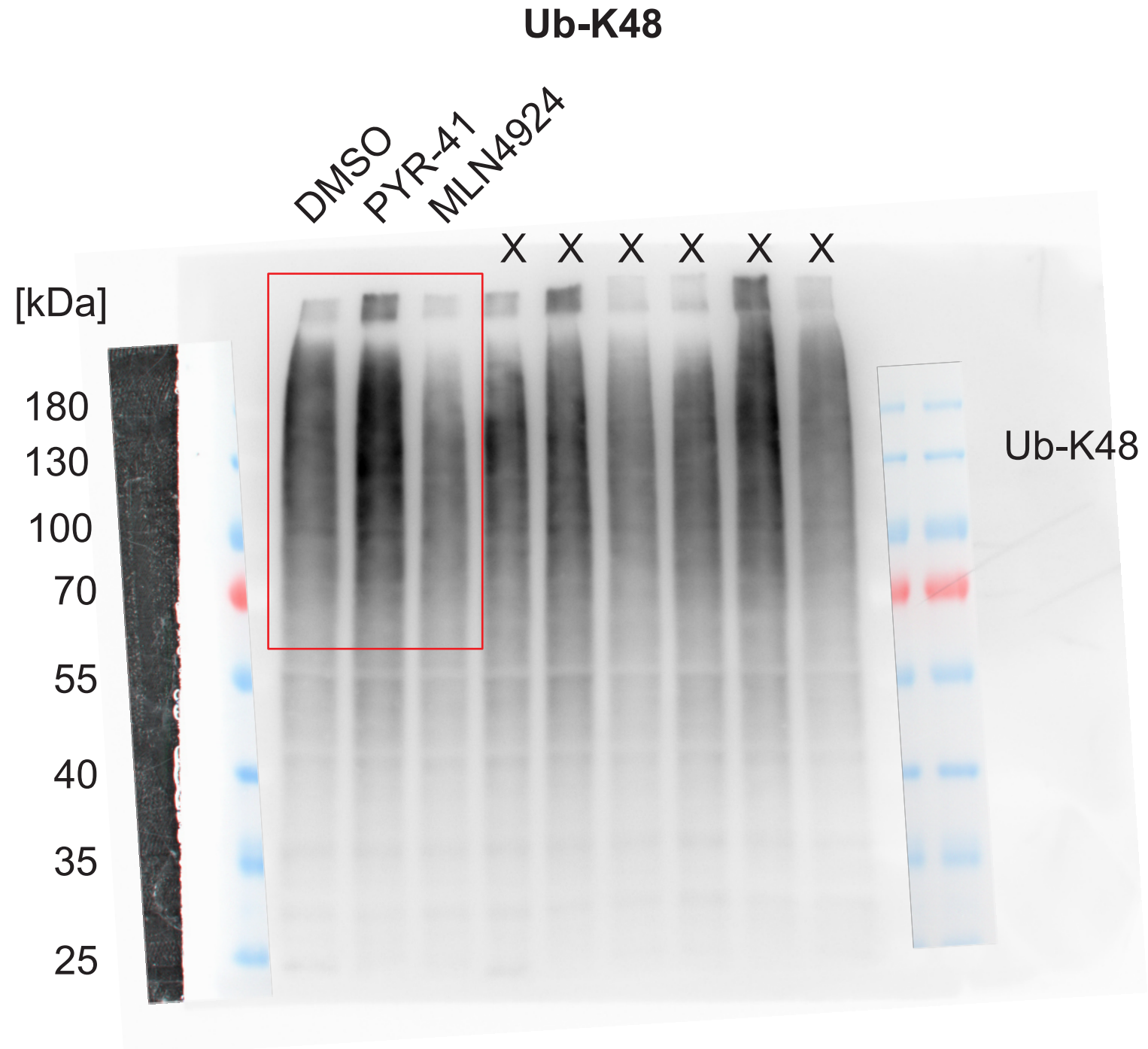

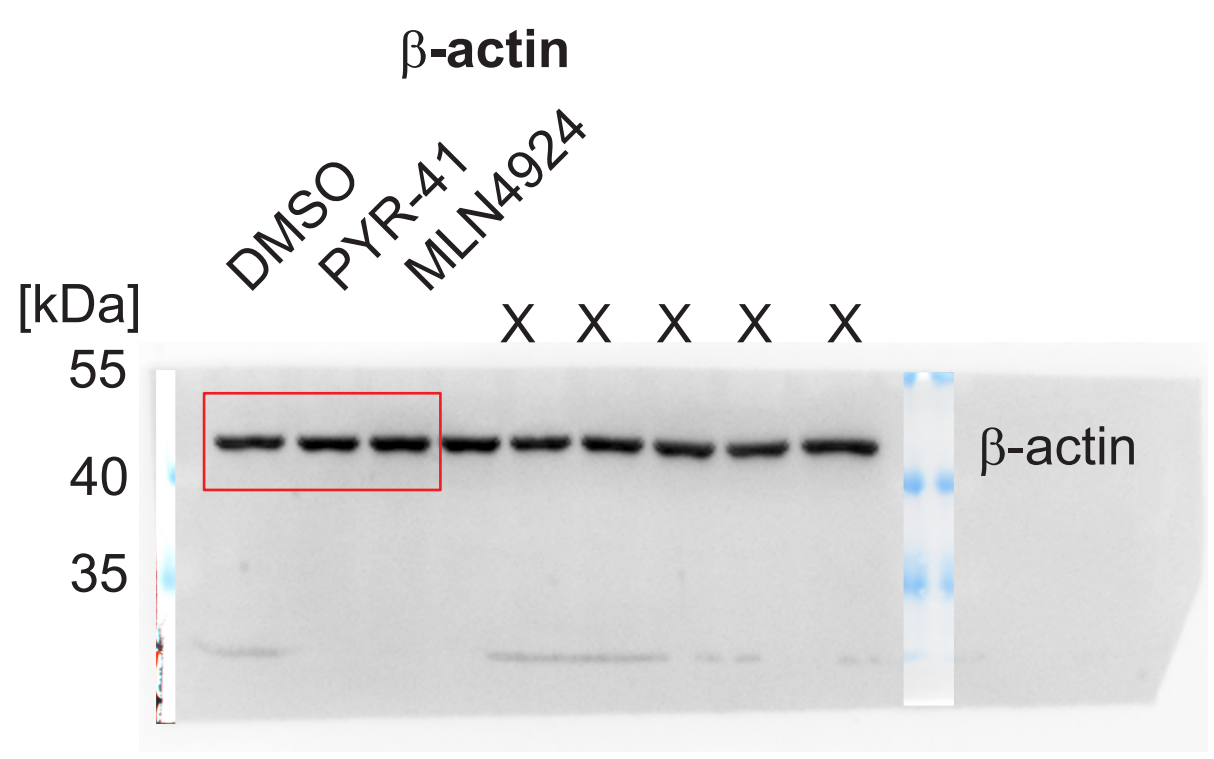

Figure **Supplementary 3B** was generated with these images
Detected with: FUSION FX7 imaging system (Vilber Lourmat, Eberhardzell, German)

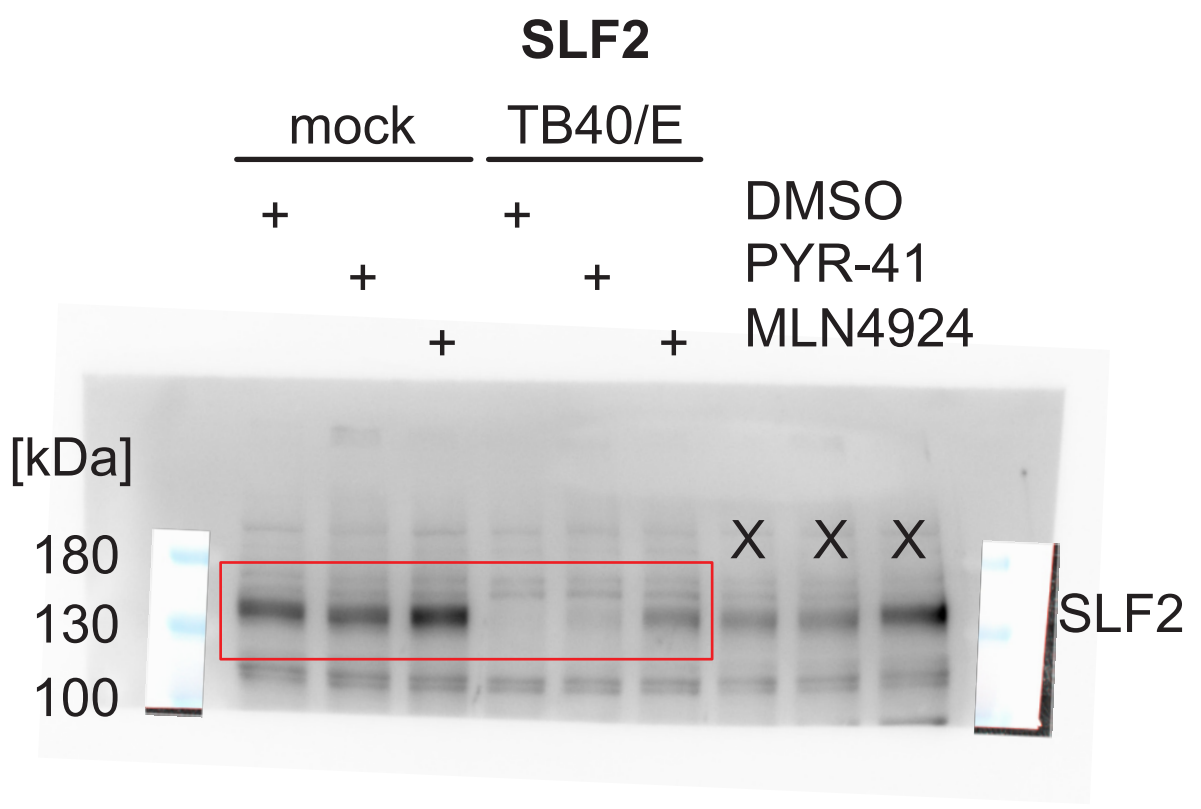

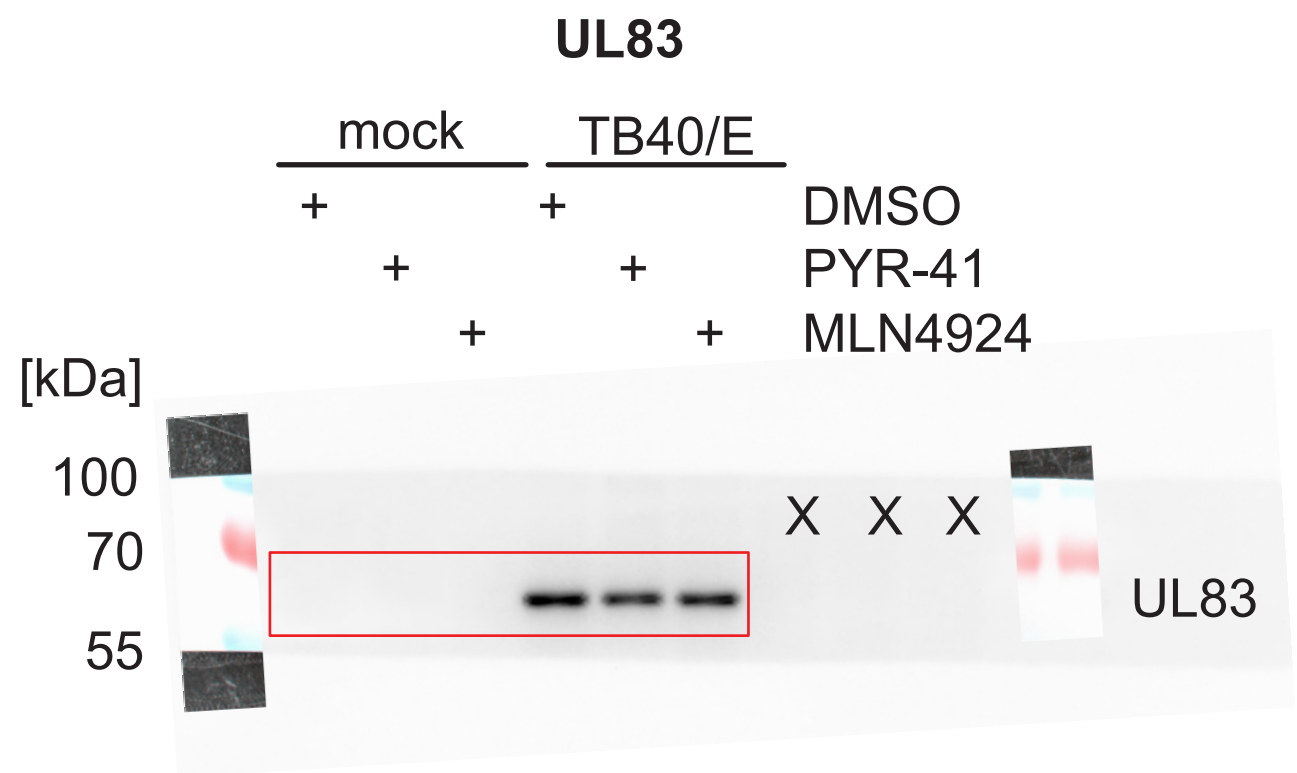

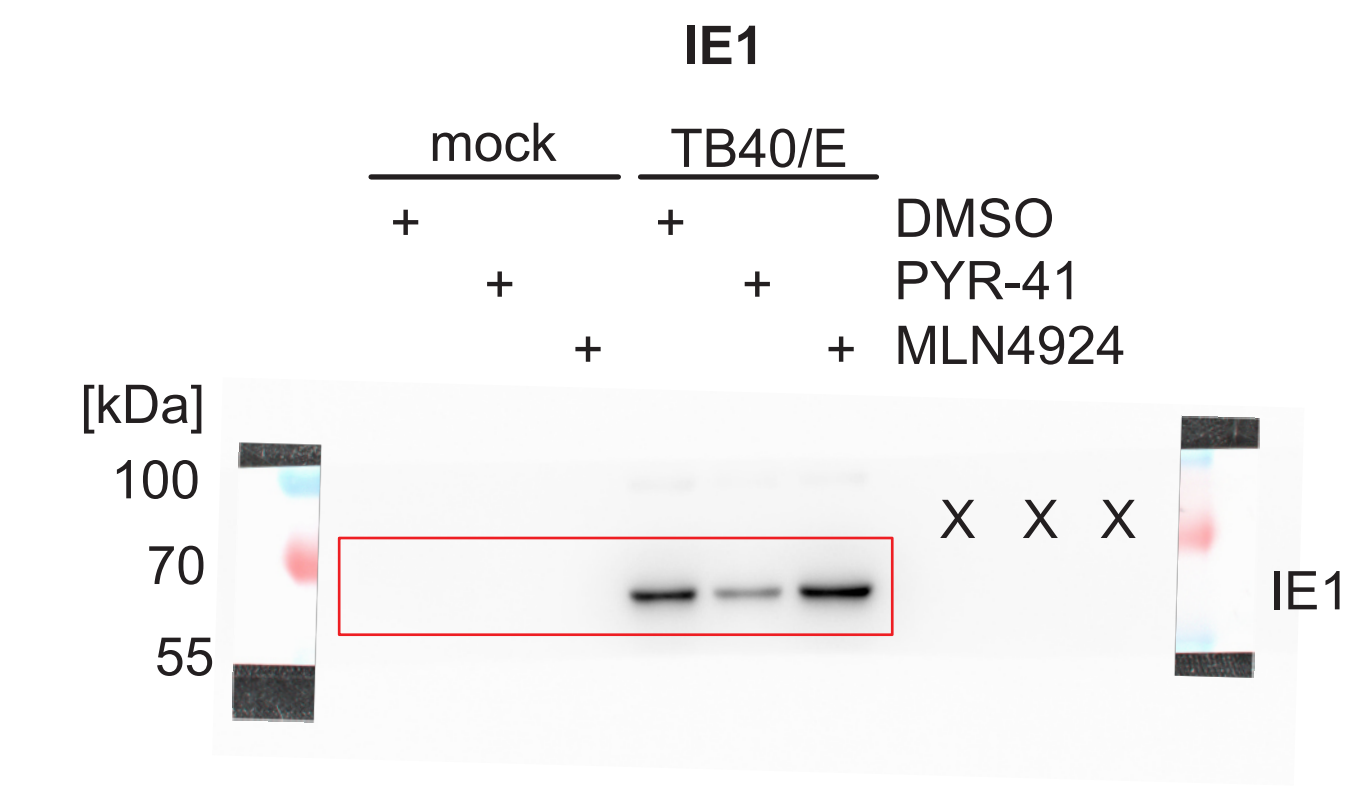

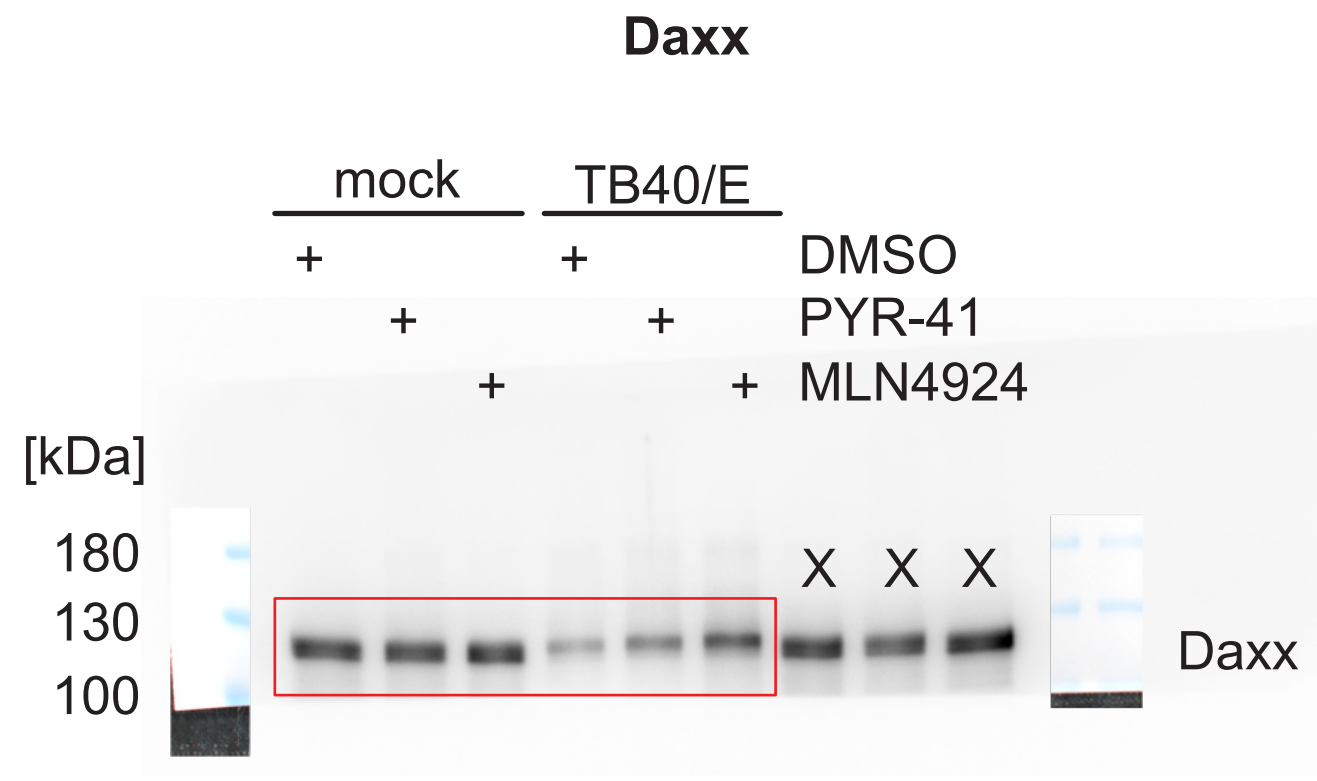

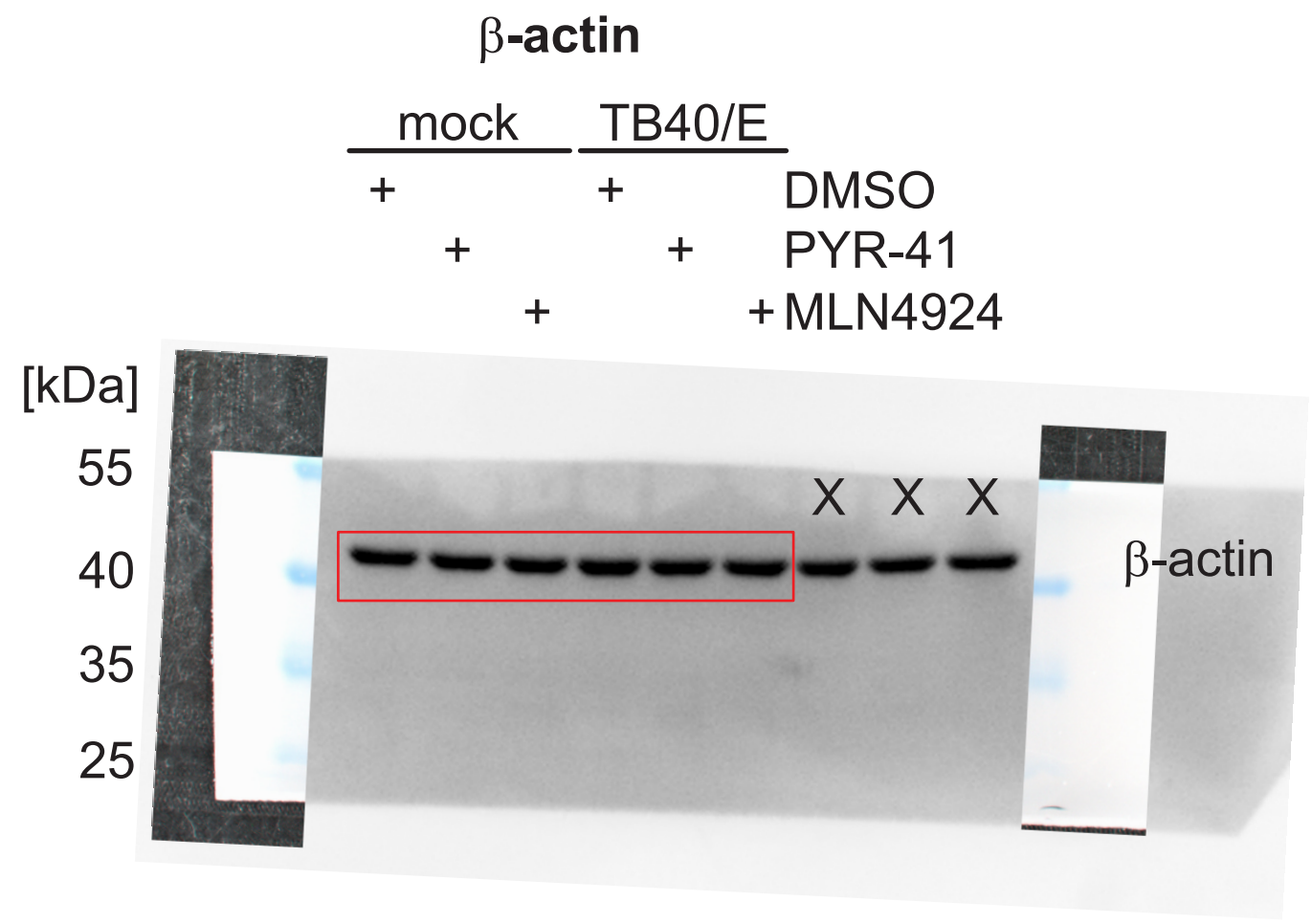

Figure **Supplementary 4C** was generated with these images
Detected with: FUSION FX7 imaging system (Vilber Lourmat, Eberhardzell, German)

IE1

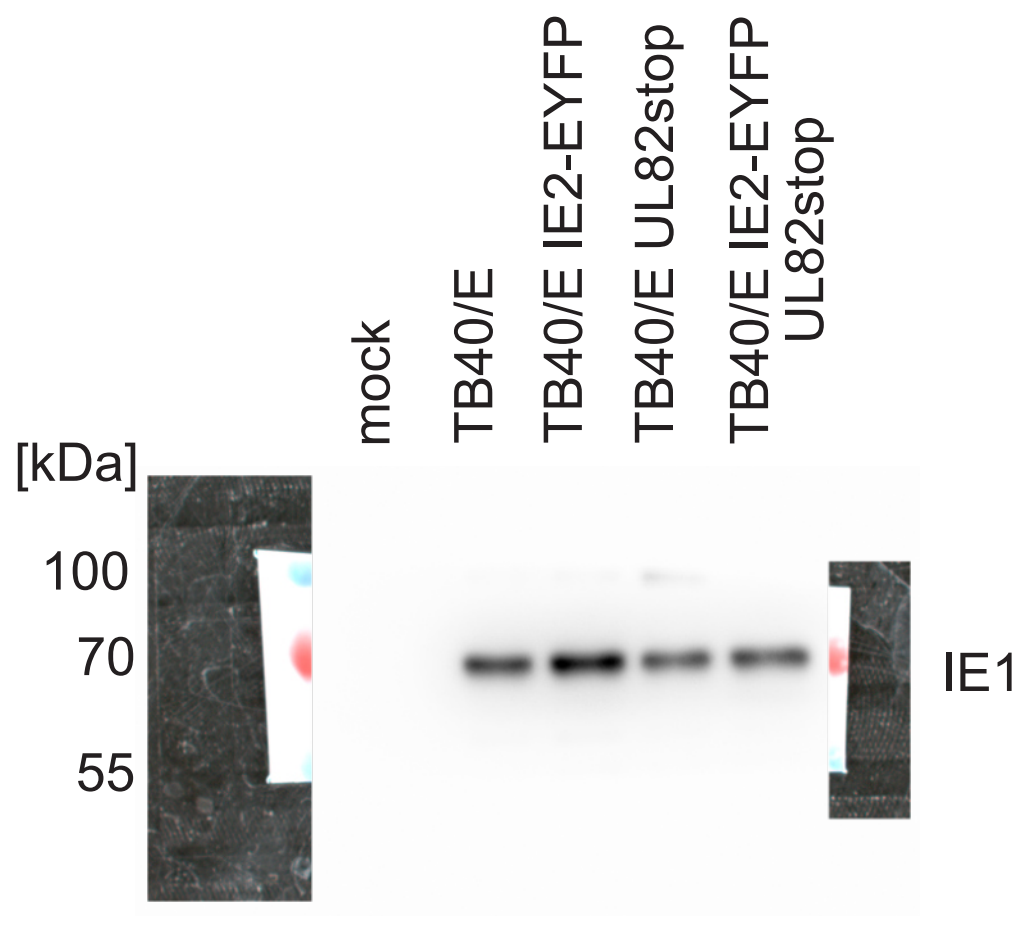

UL82

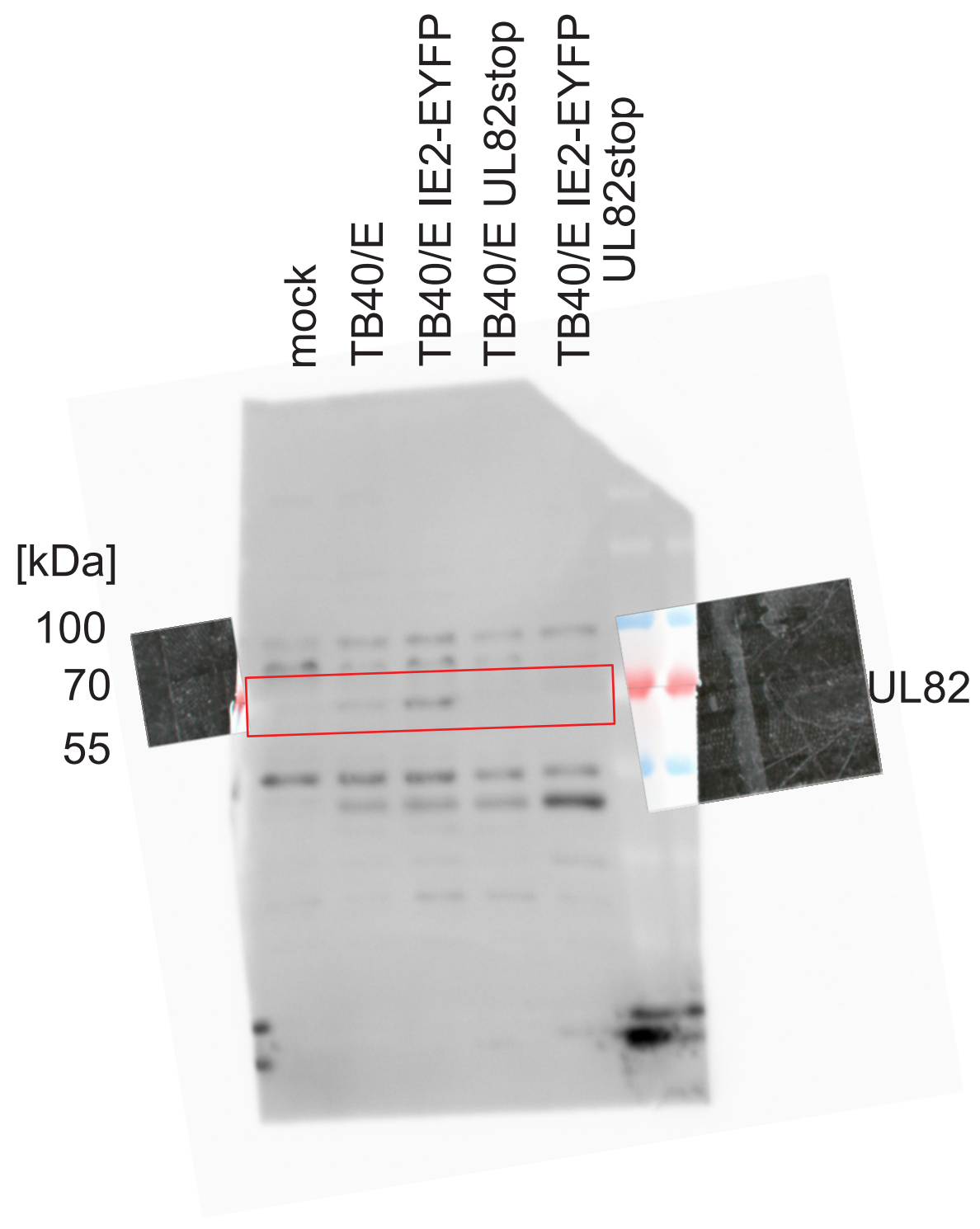

UL44

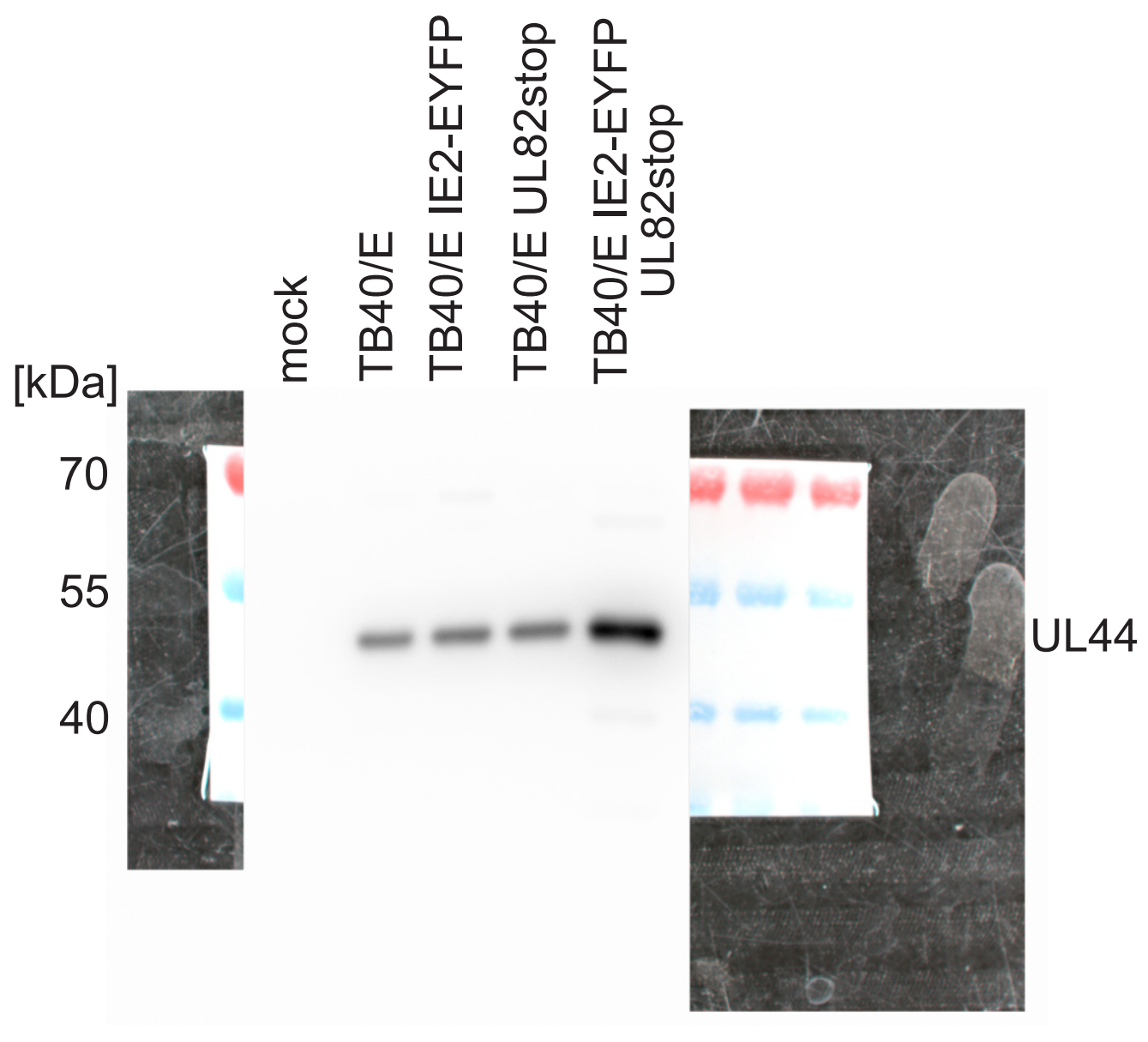

UL86

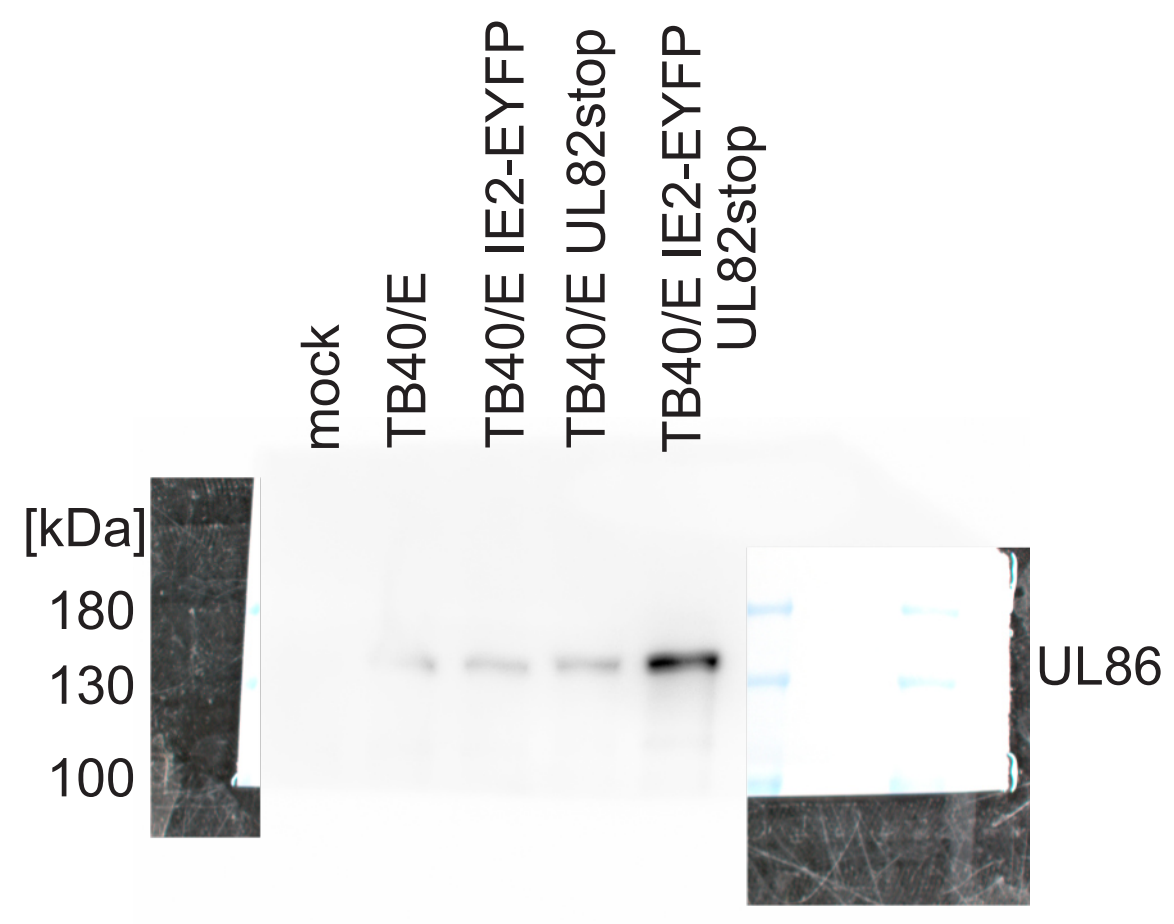

β-actin

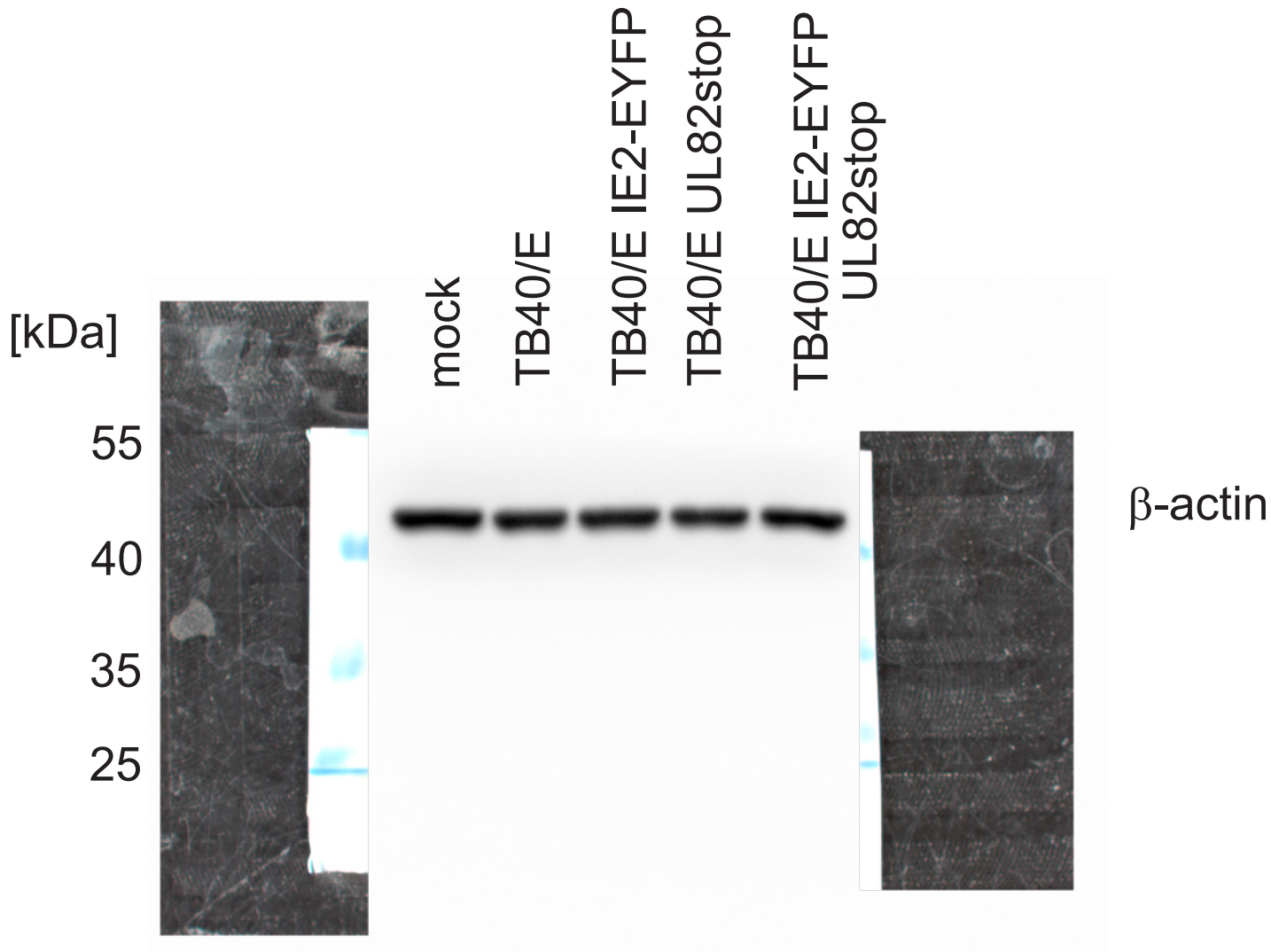

[kDa]
55
40
35
25
mock
TB40/E
TB40/E IE2-EYFP
TB40/E UL82stop
TB40/E IE2-EYFP UL82stop
β-actin
