# Supplementary material for: Human cytomegalovirus antagonizes SMC5/6 driven genome silencing via UL35 instituted proteasomal degradation of SLF2
Source: PLoS Pathog. 2026 Oct 12;22(10):e1014709. doi: 10.1371/journal.ppat.1014709 (PMC13690197; doi:10.1371/journal.ppat.1014709)
Supplement: Supplementary file 2 [file ppat.1014709.s005.docx]

**S1 Table: Key resources table**

| Key Resources Table | | | | |
| --- | --- | --- | --- | --- |
| Reagent type (species) or resource | Designation | Source or reference | Identifiers | Additional information |
| genetic reagent (Human cytomegalovirus) | TB40/E | [1]  Accession number EF999921.1 |  | Based on TB40/E-BAC4 |
| genetic reagent (Human cytomegalovirus) | TB40/E UL32-EGFP UL100-mCherry | [2]  Kindly provided by C. Sinzger (Ulm, Germany) |  | Based on TB40/E-BAC_KL7_ |
| genetic reagent (Human cytomegalovirus) | TB40/E UL128insA332 UL32-EGFP UL100-mCherry | [2]  Kindly provided by C. Sinzger (Ulm, Germany) |  | Based on TB40/E- BAC_KL7_ |
| genetic reagent (Human cytomegalovirus) | TB40/E IE2-EYFP | [3] | pHM3661 | Based on TB40/E-BAC4 |
| genetic reagent (Human cytomegalovirus) | TB40/E ΔIE1 | [4] | pHM5054 | Based on TB40/E-BAC4 |
| genetic reagent (Human cytomegalovirus) | TB40/E UL35stop | [5] |  | Based on TB40/E-BAC4 |
| genetic reagent (Human cytomegalovirus) | TB40/E UL82stop | this study | pHM5257 | Based on TB40/E-BAC4 |
| Cell line (Homo sapiens, human retinal pigmented epithelial cell) | ARPE-19 |  | ATCC-CRL-2302 |  |
| Cell line (Homo sapiens, human kidney) | HEK293T | DSMZ-German Collection of Microorganisms and Cell Cultures GmbH | Cat# ACC 635 |  |
| Cell line (Homo sapiens, human fibroblasts) | HFF expressing pW-YC1 hygro (empty vector control) | this study |  | Based on HFF-1 (ATCC SCRC-1041) |
| Cell line (Homo sapiens, human fibroblasts) | HFF expressing pW-YC1 hygro UL35HA | this study |  | Based on HFF-1 (ATCC SCRC-1041) |
| antibody | mAb-UL44 BS 510 (mouse monoclonal) | [6] |  | WB (1:5000) |
| antibody | mAb-HA HA-7 (mouse monoclonal) | Sigma-Aldrich | Cat# H9658,  RRID: AB_260092 | WB (1:5000), IF (1:250) |
| antibody | pAb-PML A301-167A (rabbit polyclonal) | Bethyl Laboratories | Cat# A301-167A,  RRID: AB_873108 | IF (1:1000) |
| antibody | pAb-PML A301-168A (rabbit polyclonal) | Bethyl Laboratories | Cat# A301-168A,  RRID: AB_2284081 | IF (1:1000) |
| antibody | pAb-Sp100 (mouse polyclonal) | Abnova | Cat# H00006672-B01,  RRID: [AB_982933](https://identifiers.org/RRID/RRID:AB_982933) | IF (1:2500) |
| antibody | mAb-β-ACTIN (mouse monoclonal) | Sigma | Cat# A5441,  RRID: AB_476744 | WB (1:10000) |
| antibody | mAb-c-myc (9E10) | Hybridoma cells, ATCC-CRL-1729 |  | WB (1:2) |
| antibody | mAb-Daxx E94 (rabbit monoclonal) | Abcam | Cat# ab32140,  RRID: AB_731847 | WB (1:1000) |
| antibody | FLAG M2 F1804 (mouse monoclonal) | Sigma | Cat# F1804  RRID: AB_262044 | IF (1:1000) |
| antibody | FLAG M2 F3165 (mouse monoclonal) | Sigma | Cat# F3165  RRID: AB_259529 | WB (1:10000) |
| antibody | mAb-GFP (mouse monoclonal) | Roche | Cat# 11814460001, RRID: AB_390913 | WB (1:5000) |
| antibody | mAb-Ub-K48 (rabbit  monoclonal) | Cell Signaling Technology | Cat# 8081S,  RRID: AB_10859893 | WB (1:1000) |
| antibody | HPR-coupled goat IgG anti-mouse | Dianova | Cat# 115-035-003  RRID: AB_10015289 | WB (1:10000) |
| antibody | HPR-coupled goat IgG anti-rabbit | Dianova | Cat# 111-035-003  RRID: AB_2313567 | WB (1:10000) |
| antibody | F(ab')2-goat anti-mouse IgG (H+L) cross-adsorbed secondary antibody, Alexa Fluor 488 | Thermo Fisher | Cat# A11029  RRID: AB_2534088 | IF (1:500) |
| antibody | Goat anti-Rabbit IgG (H+L) Highly Cross-Adsorbed Secondary Antibody, Alexa Fluor 488 | Thermo Fisher | Cat# A11034  RRID: AB_2576217 | IF (1:500) |
| antibody | Goat anti-Mouse IgG (H+L) Highly Cross-Adsorbed Secondary Antibody, Alexa Fluor 555 | Thermo Fisher | Cat# A21424  RRID: AB_141780 | IF (1:500) |
| antibody | Goat anti-Rabbit IgG (H+L) Highly Cross-Adsorbed Secondary Antibody, Alexa Fluor 555 | Thermo Fisher | Cat# A21429  RRID: AB_2535850 | IF (1:500) |
| antibody | F(ab')2-goat anti-mouse IgG (H+L) cross-adsorbed secondary antibody, Alexa Fluor 647 | Thermo Fisher | Cat# A21237  RRID: AB_2535806 | IF (1:500) |
| antibody | Goat anti-Rabbit IgG (H+L) Highly Cross-Adsorbed Secondary Antibody, Alexa Fluor 647 | Thermo Fisher | Cat# A21245  RRID: AB_2535813 | IF (1:500) |
| antibody | Goat anti-Mouse IgG2b Cross-Adsorbed Secondary Antibody, Alexa Fluor 555 | Thermo Fisher | Cat# A21147)  RRID: AB_2535783 | IF (1:500) |
| antibody | Goat anti-Mouse IgG1 Cross-Adsorbed Secondary Antibody, Alexa Fluor 488 | Thermo Fisher | Cat# A-21121, RRID: AB_2535764 | IF (1:500) |
| antibody | mAb-IE1 (mouse monoclonal) | [7] |  | WB (1:100) IF (1:5) |
| antibody | mAb-MCP 28-4 (mouse monoclonal) | [8] |  | WB (1:2) |
| antibody | mAb-Myc-Tag (71D10) (rabbit monoclonal) | Cell Signaling | Cat# 2278  RRID: AB_490778 | WB (1:500) |
| antibody | mAb-Myc-Tag (9B11) (mouse monoclonal) | Cell Signaling | Cat# 2276  RRID: AB_331783 | WB (1:500) |
| antibody | pAb-NSMCE3 (NDNL2) (rabbit polyclonal) | Proteintech | Cat# 27488-1-AP  RRID: AB_2880885 | WB (1:500) IF (1:100) |
| antibody | pAb-NSMCE4A (rabbit polyclonal) | Antikoerper-online.de | Cat# ABIN2843120 | WB (1:500) |
| antibody | mAb-PML G-8 (mouse monoclonal) | Santa Cruz | Cat# sc-377340 | WB (1:5000), IF (1:500) |
| antibody | pAb-SLF2 (rabbit polyclonal) | Abcam | Cat# ab122480  RRID: AB_11129755 | WB (1:500) IF (1:100) |
| antibody | pAb-SLF2 (rabbit polyclonal) | Merck | Cat# HPA038499  [RRID: AB_10672520](http://antibodyregistry.org/search.php?q=AB_10672520) | WB (1:500) |
| antibody | pAb-SLF2 (rabbit polyclonal) | Merck | Cat# HPA057568  [RRID: AB_2683471](http://antibodyregistry.org/search.php?q=AB_2683471) | WB (1:500) |
| antibody | pAb-SMC5 (rabbit polyclonal) | Proteintech | Cat# 14178-1-AP  RRID: AB_2192775 | WB (1:500) |
| antibody | pAb-SMC6 (rabbit polyclonal) | Abcam | Cat# ab155495 | WB (1:500) |
| antibody | mAb-SMC6 SMC6L1-M01-2E6 (mouse monoclonal) | AbceptA | Cat# AT3956a  RRID: AB_1554548 | IF (1:100) |
| antibody | pAb-STAT2 H-190 (rabbit polyclonal) | SantaCruz | Cat# sc-22816  RRID: AB_2198437 | IF (1:500) |
| antibody | mAb-UL32 (pp150) (mouse monoclonal) | [9] |  | IF (1:500) |
| antibody | mAb-UL35 (clone A.03) mouse monoclonal) | [5] |  | WB (1:5000) |
| antibody | mAb-UL69 69-66 (mouse monoclonal) | [10] |  | IF (1:2) |
| antibody | mAb-UL83 (pp65) 28-77 (mouse monoclonal) | [8] |  | WB (1:10) |
| antibody | pAb-Vpr (rabbit polyclonal) | Proteintech | Cat# 51143-1-AP  RRID: AB_10695191 | WB (1:500), IF (1:250) |
| antibody | pAb-pp71 SA2931 (rabbit polyclonal) | [11] |  | WB (1:1000) |
| antibody | pAb-pp71 SA2932 (rabbit polyclonal) | [11] |  | WB (1:5000) |
| recombinant DNA reagent (plasmid) | DH188_pCG HIV-1 NL4-3 vpr N-AU1 | Kindly provided by K. Sparrer (Ulm, Germany) |  |  |
| recombinant DNA reagent (plasmid) | GFP | [12] | pHM756 |  |
| Recombinant DNA reagent (plasmid) | pcDNA3-FLAG | [13] | pHM971 |  |
| recombinant DNA reagent (plasmid) | pCB6 UL69 | [12] | pHM160 |  |
| recombinant DNA reagent (plasmid) | pcDNA3 ppUL82-HA | this study | pHM4441 |  |
| recombinant DNA reagent (plasmid) | pcDNA3 UL35a-FLAG | this study | pHM2966 |  |
| recombinant DNA reagent (plasmid) | pcDNA3 UL35-FLAG | this study | pHM2965 |  |
| recombinant DNA reagent (plasmid) | pcDNA3 UL83-HA | this study | pHM4348 |  |
| recombinant DNA reagent (plasmid) | pcDNA3 vector containing the IE1 cDNA | [13] | pHM494 |  |
| recombinant DNA reagent (plasmid) | pCMVdeltaR8.9 | [14] |  |  |
| Recombinant DNA reagent (plasmid) | pW-YC1hygro | this study, [15] | pW-YC1blast | Blasticidin replaced with hygromycin cassette |
| Recombinant DNA reagent (plasmid) | pW-YC1hygro UL35-HA | this study |  |  |
| recombinant DNA reagent (plasmid) | pMD2.G | Addgene plasmid #12259 |  |  |
| Recombinant DNA reagent (plasmid) | psPAX2 | Addgene plasmid  #12260 |  |  |
| recombinant DNA reagent (plasmid) | pLP-VSVg | Invitrogen, Thermo Fisher Scientific, Waltham, USA |  |  |
| recombinant DNA reagent (plasmid) | pLVX Tight Puro | Clontech (Catalog No. 632162) |  |  |
| recombinant DNA reagent (plasmid) | pLVX-IRES-Neo | Takara Bio (632181) |  |  |
| recombinant DNA reagent (plasmid) | pLVX-Neo 1xmyc wt SLF2 siRNA-res (SLF2-myc) | [16]  kindly provided by Grant S. Stewart, Institute of Cancer and Genomic Sciences, University of Birmingham, Birmingham, UK |  |  |
| recombinant DNA reagent (plasmid) | pLVX-Tet-on Advanced | Clontech (Cat. No 632162) |  |  |
| recombinant DNA reagent (plasmid) | pLVX-Tight-Puro UL35a-FLAG | this study | pHM4322 |  |
| recombinant DNA reagent (plasmid) | pLVX-Tight-Puro UL35-FLAG | this study | pHM4335 |  |
| recombinant DNA reagent (plasmid) | pLV- EGFP/Puro | Vector Builder, Hennigsdorf, Germany | pF1277 |  |
| recombinant DNA reagent (plasmid) | pLV-SMC6-3xFLAG | Vector Builder, Hennigsdorf, Germany | pF1300 |  |
| chemical compound, drug | cycloheximide | Sigma | Cat# C-7698 | final concentration 150 µg/ml diluted in H_2_O. |
| chemical compound, drug | doxycycline-hyclate | Sigma-Aldrich | Cat# D5207 | diluted in H_2_O |
| chemical compound, drug | MG132 | Sigma-Aldrich | Cat# M7449-200UL | final concentration 10 µM, diluted in DMSO |
| chemical compound, drug | MLN4924 | Selleckchem | Cat# S7109 | final concentration 10 µM, diluted in DMSO |
| chemical compound, drug | PYR-41 | Selleckchem | Cat# S7129 | final concentration 5 µM, diluted in DMSO |

**References**

1.	Sinzger C, Hahn G, Digel M, Katona R, Sampaio KL, Messerle M, et al. Cloning and sequencing of a highly productive, endotheliotropic virus strain derived from human cytomegalovirus TB40/E. JGenVirol. 2008;89(Pt 2):359-68.

2.	Sampaio KL, Jahn G, Sinzger C. Applications for a dual fluorescent human cytomegalovirus in the analysis of viral entry. Methods in molecular biology (Clifton, NJ). 2013;1064:201-9. Epub 2013/09/03. doi: 10.1007/978-1-62703-601-6_14. PubMed PMID: 23996259.

3.	Wagenknecht N, Reuter N, Scherer M, Reichel A, Muller R, Stamminger T. Contribution of the Major ND10 Proteins PML, hDaxx and Sp100 to the Regulation of Human Cytomegalovirus Latency and Lytic Replication in the Monocytic Cell Line THP-1. Viruses. 2015;7(6):2884-907.

4.	Heusel AT, Rapp S, Stamminger T, Scherer M. IE1 of Human Cytomegalovirus Inhibits Necroptotic Cell Death via Direct and Indirect Modulation of the Necrosome Complex. Viruses. 2024;16(2). Epub 2024/02/24. doi: 10.3390/v16020290. PubMed PMID: 38400065; PubMed Central PMCID: PMCPMC10893529.

5.	Fabits M, Gonçalves Magalhães V, Chan B, Girault V, Elbasani E, Rossetti E, et al. The Cytomegalovirus Tegument Protein UL35 Antagonizes Pattern Recognition Receptor-Mediated Type I IFN Transcription. Microorganisms. 2020;8(6). Epub 2020/05/30. doi: 10.3390/microorganisms8060790. PubMed PMID: 32466380; PubMed Central PMCID: PMCPMC7356634.

6.	Plachter B, Nordin M, Wirgart BZ, Mach M, Stein H, Grillner L, et al. The DNA-binding protein P52 of human cytomegalovirus reacts with monoclonal antibody CCH2 and associates with the nuclear membrane at late times after infection. Virus Res. 1992;24(3):265-76.

7.	Andreoni M, Faircloth M, Vugler L, Britt WJ. A rapid microneutralization assay for the measurement of neutralizing antibody reactive with human cytomegalovirus. J VirolMethods. 1989;23(2):157-67.

8.	Waldo FB, Britt WJ, Tomana M, Julian BA, Mestecky J. Non-specific mesangial staining with antibodies against cytomegalovirus in immunoglobulin-A nephropathy. Lancet. 1989;1(8630):129-31.

9.	Sanchez V, Sztul E, Britt WJ. Human cytomegalovirus pp28 (UL99) localizes to a cytoplasmic compartment which overlaps the endoplasmic reticulum-golgi-intermediate compartment. JVirol. 2000;74(8):3842-51.

10.	Winkler M, Stamminger T. A specific subform of the human cytomegalovirus transactivator protein pUL69 is contained within the tegument of virus particles. J Virol. 1996;70(12):8984-7. Epub 1996/12/01. doi: 10.1128/jvi.70.12.8984-8987.1996. PubMed PMID: 8971028; PubMed Central PMCID: PMCPMC190996.

11.	Tavalai N, Kraiger M, Kaiser N, Stamminger T. Insertion of an EYFP-pp71 (UL82) coding sequence into the human cytomegalovirus genome results in a recombinant virus with enhanced viral growth. J Virol. 2008;82(21):10543-55. Epub 2008/08/22. doi: 10.1128/JVI.01006-08

12.	Winkler M, Rice SA, Stamminger T. UL69 of human cytomegalovirus, an open reading frame with homology to ICP27 of herpes simplex virus, encodes a transactivator of gene expression. JVirol. 1994;68(6):3943-54.

13.	Hofmann H, Floss S, Stamminger T. Covalent modification of the transactivator protein IE2-p86 of human cytomegalovirus by conjugation to the ubiquitin-homologous proteins SUMO-1 and hSMT3b. JVirol. 2000;74(6):2510-24.

14.	Zufferey R, Dull T, Mandel RJ, Bukovsky A, Quiroz D, Naldini L, et al. Self-inactivating lentivirus vector for safe and efficient in vivo gene delivery. J Virol. 1998;72(12):9873-80. Epub 1998/11/13. doi: 10.1128/jvi.72.12.9873-9880.1998. PubMed PMID: 9811723; PubMed Central PMCID: PMCPMC110499.

15.	Djakovic L, Hennig T, Reinisch K, Milić A, Whisnant AW, Wolf K, et al. The HSV-1 ICP22 protein selectively impairs histone repositioning upon Pol II transcription downstream of genes. Nature communications. 2023;14(1):4591. Epub 2023/08/01. doi: 10.1038/s41467-023-40217-w. PubMed PMID: 37524699; PubMed Central PMCID: PMCPMC10390501.

16.	Grange LJ, Reynolds JJ, Ullah F, Isidor B, Shearer RF, Latypova X, et al. Pathogenic variants in SLF2 and SMC5 cause segmented chromosomes and mosaic variegated hyperploidy. Nature communications. 2022;13(1):6664. Epub 2022/11/06. doi: 10.1038/s41467-022-34349-8. PubMed PMID: 36333305; PubMed Central PMCID: PMCPMC9636423.
