# Supplementary material for: Human cytomegalovirus antagonizes SMC5/6 driven genome silencing via UL35 instituted proteasomal degradation of SLF2
Source: PLoS Pathog. 2026 Oct 12;22(10):e1014709. doi: 10.1371/journal.ppat.1014709 (PMC13690197; doi:10.1371/journal.ppat.1014709)
Supplement: Supplementary file 3 [file ppat.1014709.s006.docx]

**S2 Table. Oligonucleotides**

| Oligonucleotides for real-time PCR (TaqMan) | | |
| --- | --- | --- |
| Name | Sequence | Note |
| Albumin-rev (21-61) | 5’-GCATGGAAGGTGAATGTTTCAG-3’ |  |
| Albumin-fw (21-65) | 5’-GTGAACAGGCGACCATGCT-3’ |  |
| CMV-fw (21-69) | 5’-AAGCGGCCTCTGATAACCAAG-3’ | Used also for SYBR-Green PCR |
| CMV-rev (21-70) | 5’-GAGCAGACTCTCAGAGGATCGG-3’ | Used also for SYBR-Green PCR |
| ALB FAM/TAMRA (21-64) | 5’-TCAGCTCTGGAAGTCGATGAAACATACGTTC-3’ |  |
| CMV MIE FAM/TAMRA (21-68) | 5’-CATGCAGATCTCCTCAATGCGGCG-3’ |  |
| Oligonucleotides for SYBR-Green PCR | | |
| IE2-fw (61-39) | 5´-TGACCGAGGATTGCAACGA-3´ |  |
| IE2-rev (61-40) | 5´-CGGCATGATTGACAGCCTG-3´ |  |
| huGAPDH_TM_fw (22-00) | 5´-GAAGGTGAAGGTCGGAGT-3´ |  |
| huGAPDH_tm_rev (22-01) | 5´-GAAGATGGTGATGGGATTTC-3´ |  |
| SLF2-fw (64-94) | 5´-CCAAGGCAGGAACAAAGGAAACAG-3´ | [1] |
| SLF2-rev (64-95) | 5´-CTGCTTAGAGCCTGCTGAAGTAGG-3´ | [1] |
| Oligonucleotides for cloning purposes | | |
| HA_pp71_EcoRI-fw (47–82) | 5´-GCATGAATTCATGTACCCATACGATGTTCCAGATTACGCTTCTCAGGCATCGTCCTCGCCCGG-3´ |  |
| pp71_XhoI-rev (47-83) | 5´-AGTACTCGAGCTAGATGCGGGG TCGACTGC-3´ |  |
| UL83_BamHI-fw (27-72) | 5´-CGATGGATCCAATGGAGTCGCGCGGTCG-3´ |  |
| UL83_(561)_HA_XhoI-rev (46-58) | 5´-TGACTCGAGTTAAGCGTAATCTGGAACATCGTATGGGTAACCTCGGTG CTTTTTGGGCG-3´ |  |
| UL35-ClaI-fw (26-95) | 5´-TTCAATCGATGCCCAAGGATCGCGAGCCCCATCG-3´ |  |
| UL35-XhoI-rev (26-96) | 5´-TTCACTCGAGTCAGAGATGCCGTAGGTTTTCGGC-3´ |  |
| UL35A-ClaI-fw (26-97) | 5´-TTCAATCGATATGATCGAGGGCGCCTCTCGG-3´ |  |
| Flag_UL35_NotI-fw (46–49) | 5´-CATAGCGGCCGCATGGACTACAAAGACGATGACGATAAAGCCCAAGGATCGCGAGCCCCATCG–3´ |  |
| UL35_MluI-rev (46-50) | 5´-CATAACGCGTTCAGAGATGCCGTAGGTTTTC-3´ |  |
| FlaG_UL35a_BamHI-fw (45–85) | 5´-CATAGGATCCATGGACTACAAAGACGATGACGATAAAATCGAGGGCGCCTCTCGGCAG-3´ |  |
| UL35_NotI-rev (45-84) | 5´-CATAGCGGCCGCTTAGAGATGCCGTAGATTTTC-3´ |  |
| BamHi-Hygro_For | 5’GCATGGATCCACCATGGATAGATCCGGAAAGC-3’ |  |
| XbaI-Hygro_Rev | 5‘-CCATTCTAGACATTGGACCAGGGTTTTCTTCAA CATCACCACAAGTGAGGAGAGAACCTCTACCTT CGCCGCTTCCTTCCTTTGCCCTCGGAC-3‘ |  |
| NheI_UL35HA_For | 5’-GCATGCTAGCAAGCTTATGGCCCAGGGATCGCGAG-3’ |  |
| MluI_UL35HA_Rev | 5’CCATACGCGTCTATGCGTAGTCTGGTCGTCGTACGGATATG-3’ |  |
| ON-TARGETplus siRNA Smart Pools (Horizon Discovery) | | |
| Target | Designation | Source |
| siC | ON-TARGETplus Non-targeting Control Pool | Horizon Discovery (D-001810-10-05) |
| siCUL4a | siGENOME human CUL4a siRNA SMARTpool | Horizon Discovery, Cat# M-012610-01-0005 |
| siDCAF1 | siGENOME human DCAF1 siRNA SMARTpool | Horizon Discovery, Cat# M-021119-01-0005 |
| siOGT | siGENOME human OGT siRNA SMARTpool | Horizon Discovery, Cat# M-019111-00-0005 |
| siSLF2 | ON-TARGETplus Human SLF2 siRNA (Human) | Horizon Discovery (L-016268-01-0005) |
| siSMC6 | ON-TARGETplus Human SMC6 siRNA-SMARTpool | Horizon Discovery, Cat# L-018408-01-0005 |

**References**

1.	Wu T, Li Y, Zhao Y, Bournique E, Ortega P, Nie M, et al. The SMC5/SMC6 complex is critical for resolving R-loop-induced transcription-replication conflicts. Nucleic Acids Res. 2026;54(2). Epub 2026/01/15. doi: 10.1093/nar/gkaf1537. PubMed PMID: 41533569; PubMed Central PMCID: PMCPMC12802942.
